# AP‐Lab: An AI‐Driven Autonomous Pilot‐Scale Platform Bridging Materials Discovery and Industrial Manufacturing

**DOI:** 10.1002/advs.74293

**Published:** 2026-02-12

**Authors:** Zhan‐Long Wang, Zhifen Ma, Wenxing Song, Guolai Jiang, Boshi Jiang, Mingyang Jiang, Weiliang Shu, Bing Wang, Zhiyuan Wan, Shengyong Geng, Zhen Zhao, Wenhua Zhou, Xue‐Feng Yu

**Affiliations:** ^1^ Center for Materials Artificial Intelligence Shenzhen Institutes of Advanced Technology Chinese Academy of Sciences Shenzhen Guangdong China; ^2^ Shenzhen Small and Medium‐sized Pilot Base for AI‐driven Material Creation and Manufacture Shenzhen Guangdong China; ^3^ Shenzhen Archean Biotechnology Co., Ltd Shenzhen Guangdong China; ^4^ Key Laboratory of Biomedical Imaging Science and System Chinese Academy of Sciences Shenzhen Guangdong China

**Keywords:** application‐oriented performance benchmark, autonomous pilot‐scale laboratory, closed‐loop optimization, industrial datasets, magnetic nanoparticles

## Abstract

Artificial intelligence (AI) has accelerated materials discovery, yet its translation to industrial manufacturing remains limited due to two critical gaps: the scarcity of proprietary industrial datasets and the absence of application‐oriented benchmarks. To address these challenges, we develop the AP‐Lab, an AI‐Driven Autonomous Pilot‐Scale Laboratory workstation designed to bridge research and manufacturing. Using magnetic nanoparticles (MNPs) for viral nucleic acids (NAs) extraction as a case study, the AP‐Lab integrates four agent‐controlled systems for user interaction, optimization scheme generation, autonomous synthesis and testing, and data management. By leveraging localized industrial datasets and adopting Polymerase Chain Reaction (PCR) cycle threshold (Ct) values as an application‐specific benchmark, the AP‐Lab achieves rapid optimization of MNPs‐based NAs extraction products at pilot‐scale corresponding to 50,000 tests per batch within three weeks, and enables scale‐up manufacturing of 1 million tests per batch in two months. Compared to conventional manual workflows, the AP‐Lab reduces development timelines from four to six months to three weeks while delivering performance superior to leading commercial products. This work demonstrates a scalable strategy for AI‐driven pilot‐scale production and offers a blueprint for accelerating industrial adoption of advanced materials.

## Graphical abstract

AP‐Lab bridges materials discovery and industrial manufacturing by coupling proprietary datasets with an application benchmark (PCR Ct). A closed‐loop optimization workflow integrates ML, LLM, and autonomous synthesis/testing to refine magnetic nanoparticles–based nucleic‐acid extraction systems. The resulting products outperform leading commercial kits and scale to 1 million‐test production per batch in under two months with high consistency.

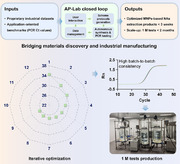

## 1 Introduction

Recently, artificial intelligence (AI) technologies, including machine learning (ML), large language models (LLMs), and autonomous robotics, significantly accelerate research across diverse domains [1, 2, 3, 4, 5, 6, 7, 8]. Particularly in materials science, the applications of AI has shown great potential in discovering new materials [9, 10], such as catalysts [11], colloidal nanocrystals [12], and battery materials [13]. For example, Szymanski et al. predicted over 2.2 million potential materials using the GNOME model and successfully synthesized 41 of them [14]. Cronin and co‐workers developed the Chemputer, a robotic platform that autonomously executes chemical synthesis extracted from the literature data, resulting in the discovery of multiple new materials [15]. The Jamison and Jensen teams created a system combining AI‐driven synthesis planning and a robotically controlled experimental platform for compound synthesis, and significantly accelerated the synthesis of complex organic molecules [16]. These remarkable AI‐driven progresses have been achieved in addressing challenges in fundamental science [17]. However, applications of these techniques in the advanced materials manufacturing industry, although urgently in high demand, remain largely unexplored [18, 19, 20].

This situation is fundamentally attributed to the lack of proprietary industrial datasets and the absence of application‐oriented benchmarks in AI‐driven materials research. First, despite the rapid development of AI models, particularly in ML algorithms, origin of data is a crucial factor in determining the outcomes of ML projects [21, 22, 23]. Unlike the aggregated scientific literature datasets that is publicly accessible, proprietary industrial datasets are seldom published due to concerns regarding competition and intellectual property. Such limited availability of reliable industrial datasets as initial inputs poses significant challenges for AI‐driven approaches from the outset. Second, current AI and automation‐driven approaches still mainly focus on solving scientific questions that are normally regarded as “low‐level details” in the view of manufacturing industries in materials [20, 24, 25]. Extrapolating overall performance in specific applications from these low‐level metrics is highly challenging. Thus, overlooking these issues has led to a situation where no existing autonomous platform can efficiently support pilot‐scale optimization and industry‐scale translation.

We hypothesize that integrating proprietary industrial datasets and application‐oriented benchmarks with AI‐driven iterative optimization can drastically accelerate pilot‐scale materials development. Therefore, we develop an AI‐Driven Autonomous Pilot‐Scale Laboratory (AP‐Lab) workstation for materials pilot‐scale manufacturing, with magnetic nanoparticles (MNPs), the core material products in the field of medical diagnostics, as a typical case study. Contrast with existing autonomous laboratories, such as A‐Lab, Chemputer, etc., the AP‐Lab focuses on pilot‐scale development of material products instead of laboratory‐scale. Additionally, the AP‐Lab is trained with industrial datasets rather than scientific literature datasets, and adopts Polymerase Chain Reaction (PCR) cycle threshold (Ct) values in viral nucleic acids (NAs) extraction as application‐oriented benchmarks rather than low‐level metrics. During the autonomous high‐throughput synthesis and testing on the AP‐Lab, ten categories of key parameters are selected, and ML‐based iterative optimization together with LLM‐driven reagent recommendations are employed. Through the proposed AP‐Lab, we achieve significant optimization in viral NAs extraction at the pilot production level corresponding to 50,000 tests per batch within three weeks, with the extraction efficiency outperforming the current leading commercial products. A scale‐up manufacturing line for MNPs and NAs extraction reagents mass production corresponding to 1 million tests per batch is subsequently established within two months according to key process nodes identified by the AP‐Lab, without observable performance changes as compared with pilot‐scale production. This work demonstrates a scalable strategy for AI‐driven pilot‐scale production by integrating industrial datasets and application‐oriented benchmarks, and may offer a blueprint for accelerating industrial adoption of other advanced micro‐ and nano‐materials.

## 2 Results and Discussion

### 2.1 The AP‐Lab Design and Workflow

**FIGURE 1:**
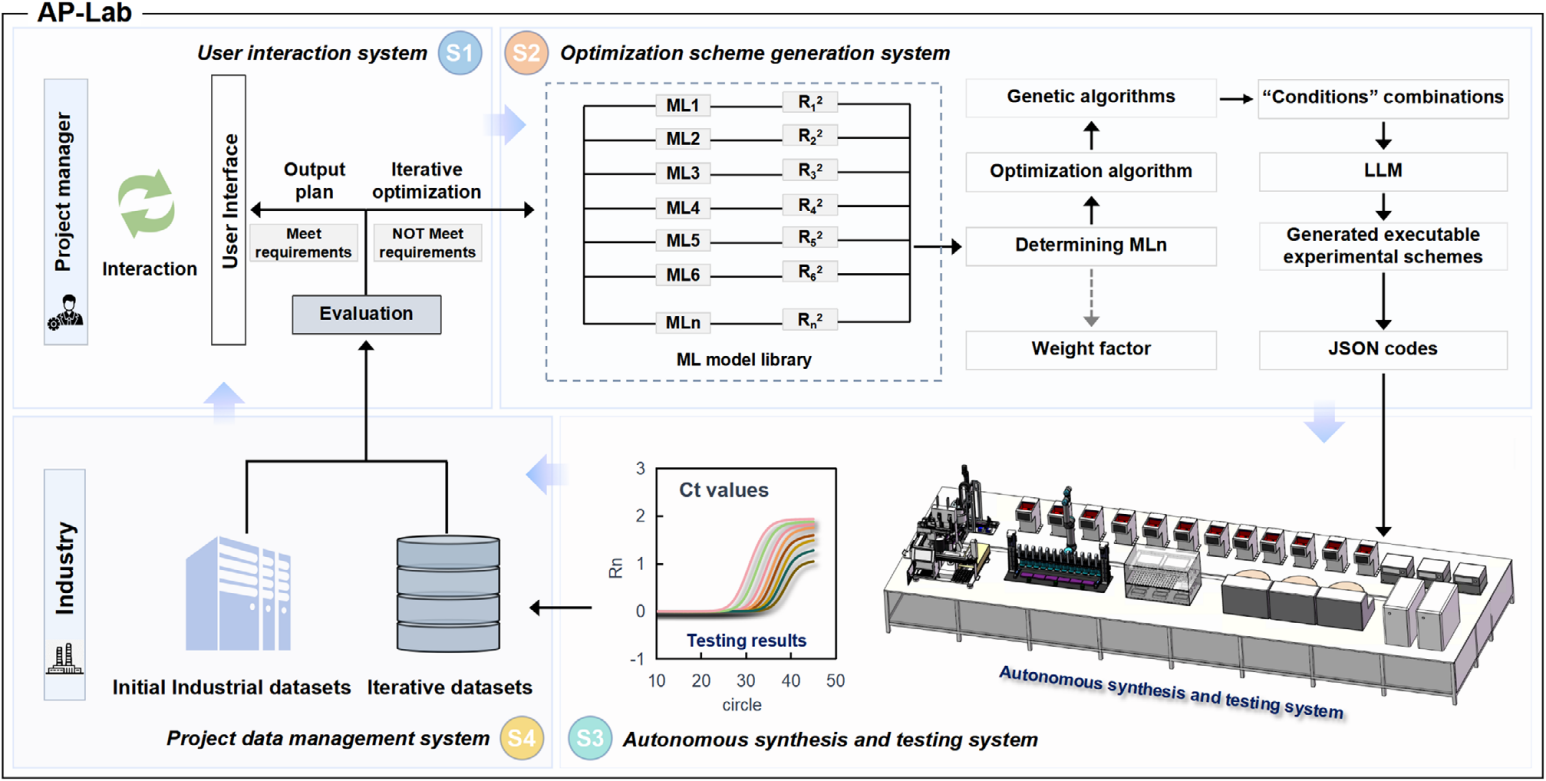
Workflow of the pilot‐scale product optimization process in the AP‐Lab workstation. The process starts with the project manager specifying initial requirements, including input subtypes and expected Ct values. The user interaction system (S1) retrieves and evaluates relevant industrial datasets from the project data management system (S4). If no suitable formulation data are found, the datasets are submitted to the optimization scheme generation system (S2), where multiple ML models are trained, and the best model is selected based on R^2^ values. Using a genetic algorithm, 48 sub‐grouped “*Conditions*” combinations are proposed. Then, the detailed executable schemes are generated and converted into JSON codes by the LLM. These schemes are executed by the autonomous synthesis and testing system (S3), which outputs Ct values to the project data management system (S4). The user interaction system again evaluates the results and determines whether further iterations are required.

To address the challenges in pilot‐scale optimization and production, we design and implement the AP‐Lab, an integrated workstation combining AI‐driven optimization algorithms and a high‐throughput autonomous experimentation platform. Figure 1 illustrates the conceptual framework and operational workflow of the established AP‐Lab, which includes four individual systems, that is, a user interaction system (S1), an optimization scheme generation system (S2), an autonomous synthesis and testing system (S3), and a project data management system (S4). In the workflow of the AP‐Lab, the datasets are categorized into ten sub‐grouped “*Conditions*” that significantly affect MNPs preparation and NAs extraction performances, as inputs, including *particle size*, *surfactant* (e.g., Tween 20), *salting out reagent* (e.g., sodium chloride), *buffer* (e.g., Tris‐HCl), *functional group* (e.g., carboxyl), and so on, which are suggested by skilled engineers (Table S1). Each “*Condition*” is then expanded into a group of sub‐conditions, according to the actual conditions involved in the tests. Since Ct values are the ultimate indicator reflecting the overall performance of the prepared reagents and MNPs, they are selected as the only application‐oriented benchmark outputs.

In a typical pilot‐scale product optimization process, a project manager first specifies initial requirements, including input “*Condition*” subtypes they wish to investigate and Ct values they expect in the user interface (Figure S1). Once the inputs are received, the user interaction system (S1), which enables the interaction between the AP‐Lab and project managers, analyzes the request, retrieves, and evaluates relevant initial industrial datasets from the project data management system (S4). If the datasets contain product formulation data that meet the requirements, the detailed formulation is directly displayed on the user interface. Otherwise, S1 submits the data to the optimization scheme generation system (S2) to generate an experimental plan based on the initial industrial datasets. Then, an ML model library (containing over 60 commonly used supervised regression and optimization algorithms) is employed to generate optimized formulations. Specifically, the data are trained using multiple supervised regression algorithms, and the best‐performing models are selected based on their R^2^ scores. Meanwhile, two settings, with and without LLM‐recommended parameter suggestions, are tested to identify the better‐performing strategy (Figures S2 and S3). For the MNPs‐based NAs extraction products, random forest consistently shows the strongest performance across iterative rounds, and incorporating the LLM‐recommended parameter sets further improves performance (Figure S4). Therefore, random forest is used as the primary regression model for subsequent training, and the LLM‐recommended parameter suggestions are incorporated into the optimization loop. Potential overfitting is monitored and mitigated by tracking the evolution of R^2^ and mean squared error (MSE) across iterations (Figure S5). The selected regression model is coupled with genetic algorithms to recommend up to 48 sub‐grouped “*Conditions*” combinations with calculated Ct values closest to the manager's requirements. Once experimental plans are generated, the LLM is then employed to generate detailed executable experimental schemes and convert these schemes into JSON codes. The autonomous synthesis and testing system (S3, Figure S6–S9) subsequently carries out experiments according to the JSON codes and ultimately gives Ct values for each scheme as outputs to the project data management system (S4).

After each round of iterative experiments, the user interaction system (S1) is invoked to evaluate the newly generated data. If the results meet the predefined requirements specified by the project manager, the corresponding formulations are displayed on the user interface. Otherwise, the AP‐Lab proceeds to the next iteration by jointly leveraging the initial industrial datasets and the newly acquired experimental results. Additionally, ML training of the data not only produces R^2^values but also yields weighting factors (as indicated by the gray dashed lines in S2, Figure 1), enabling a deeper understanding of how each condition influences MNPs preparation and reagent matching.

The AP‐Lab is built upon a closed‐loop system philosophy that integrates ML with autonomous experimental execution. Its architecture separates decision‐making from physical operation, enabling AI‐driven models to design experiments while modular robotic units carry out synthesis, processing, and testing. All data, including formulation conditions and workflow instructions, are represented in standardized machine executable formats, ensuring reproducibility across iterations and facilitating scalability from laboratory‐scale screening to pilot‐scale production. By incorporating industrial datasets, iterative high‐throughput datasets, and automated reasoning via LLMs and ML models, the AP‐Lab establishes an adaptive, self‐improving workflow capable of converging on optimal material products.

### 2.2 Performance and Reproducibility

**FIGURE 2:**
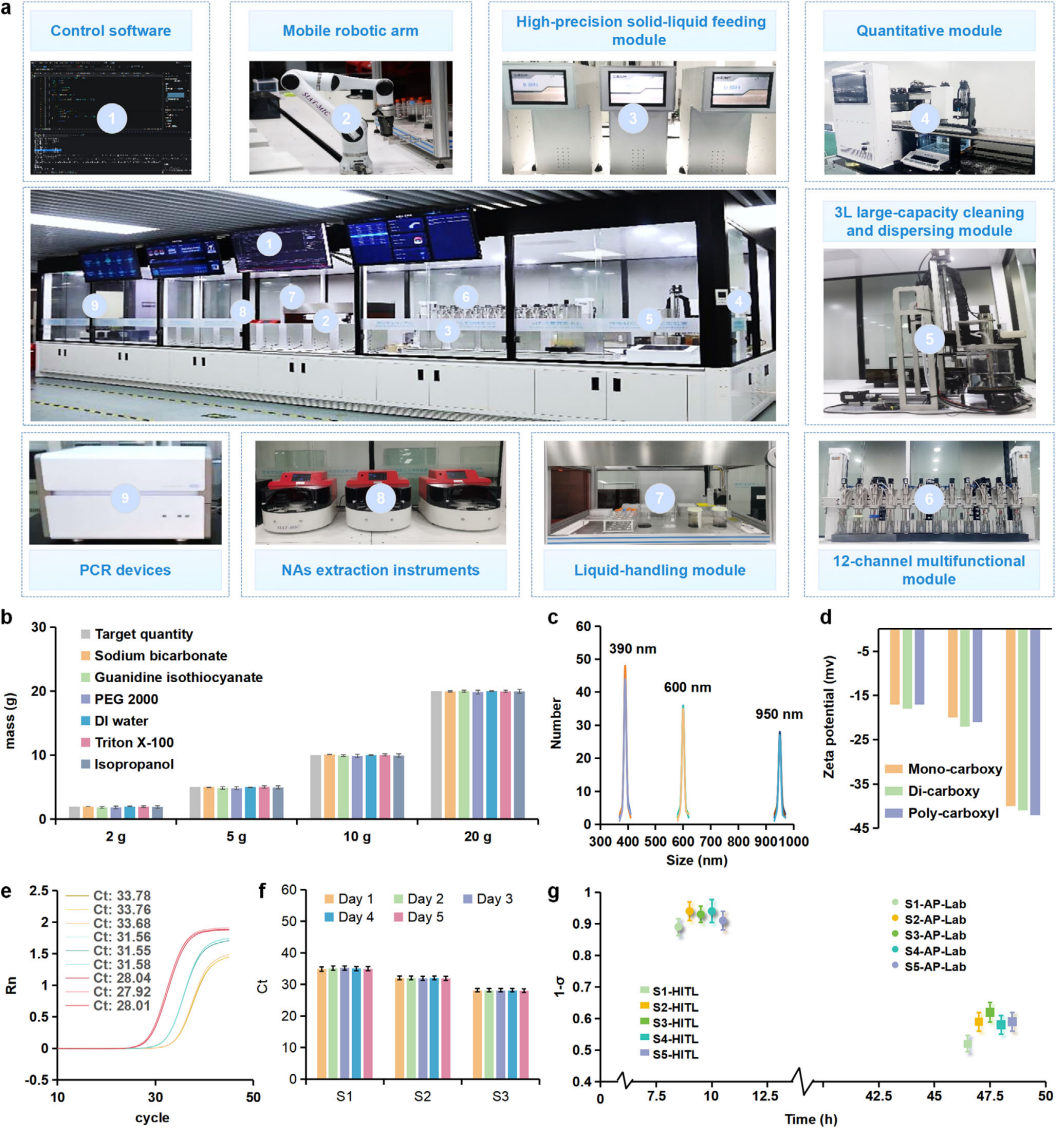
Structure and performance evaluation of the autonomous synthesis and testing system. (a) The system is designed as a precision‐controlled island‐structured platform equipped with custom‐designed modules for high‐precision solid‐liquid feeding, solution quantifying, large‐capacity cleaning and dispersing, MNPs modification, and liquid handling. (b) Consistency of solid and liquid reagent preparation using sodium bicarbonate, guanidine isothiocyanate, PEG2000, DI water, Triton X‐100, and isopropanol across different measurement ranges (2, 5, 10, and 20 g), demonstrating stable performance with error within 3% for most reagents. (c) Particle size distributions of silica‐coated MNPs prepared with different reaction times, showing highly consistent size distributions centered around 390, 600, and 950 nm across batches. (d) Zeta potential analysis of silica‐coated MNPs with carboxyl modifications at different densities, demonstrating consistent values of −17 ± 1 mV, −20 ± 1 mV, and −40 ± 1 mV for mono‐, di‐, and poly‐carboxyl groups, respectively. (e) Batch‐to‐batch consistency in NAs extraction as indicated by stable Ct values across tests with different pseudovirus concentrations (yellow: 10^3^ copies/mL, green: 10^4^ copies/mL, and red: 10^5^ copies/mL). (f) Temporal stability confirmed over five consecutive days using SARS‐CoV‐2 pseudovirus samples with a concentration of 10^5^ copies/mL using different extraction reagent systems. (g) Comparison of the AP‐Lab and HITL experiments, showing the AP‐Lab's superior efficiency (7–12 h for the AP‐Lab vs. 45–50 h for HITL) and improved reproducibility (consistency score ≥0.9 for the AP‐Lab vs. 0.5–0.7 for HITL).

The stability and consistency of the synthesis and testing processes are critical for materials fabrication and performance validation, because even minor deviations in reagent amount, composition, or concentration can lead to pronounced variations in surface chemistry and functional group density, ultimately compromising performances at application‐levels [26, 27]. Thus, to overcome the reproducibility challenges, the autonomous synthesis and testing system (S3) is rigorously engineered. It is constructed as a standalone, island‐structured platform to ensure operational independence and mechanical stability (Figure 2a; Figure S6), thereby enabling fully reproducible MNPs coating and modification, reagent preparation, NAs extraction, and PCR validation workflows. This system is composed of control software and a series of custom‐designed modules. A mobile robotic arm is mounted on the system, allowing reciprocal movements of various containers across the platform among different modules seamlessly. The solid‐liquid feeding module is designed to accurately dispense solid and liquid reagents with high precision. 12 such modules are deployed to enable preparation of diverse reagents for both MNPs modification and NAs extraction. The quantitative module is built to precisely quantify the concentration of MNPs, ensuring consistency among different steps (Figure S7). The 3L large‐capacity cleaning and dispersing module is responsible for ultrasonic mixing, mechanical stirring, and solution changing, which together facilitate the cleaning and dispersing operations of massively produced magnetic cores (Figure S8). The 12‐channel multifunctional module can achieve simultaneous or independent MNPs modification operation across 12 channels (500 mL container) with reagent heating reactions, cleaning, and separation functions (Figure S9). In addition to these key modules, the platform also integrates a custom‐purchased liquid‐handling module for liquid feeding in 96‐well deep‐well plates, and NAs extraction instruments coupled with PCR devices for efficient performance evaluation of MNPs and reagents in NAs extraction.

Three critical steps are rigorously measured for consistency evaluation: reagent dispensing accuracy, the particle size of MNPs after surface modification, and the density of surface functional groups. To assess dispensing accuracy, six representative reagents, including sodium bicarbonate (granular solid), guanidine isothiocyanate (crystalline powder), PEG2000 (waxy solid), DI water (low‐viscosity liquid), Triton X‐100 (high‐viscosity liquid), and isopropanol (volatile liquid), are examined. Four target quantities (2, 5, 10, and 20 g) are tested for all reagents (average values of five measurements). In Figure 2b, the results show that all reagents exhibit good consistency. For most reagents, the error is less than 3% with a precision higher than 100 mg (Tables S2 and S3). These tests demonstrate the stability of high‐precision reagent feeding on the autonomous system, ensuring the validity and reproducibility of experiments across batches. Figure 2c,d presents diameters and Zeta potentials of silica‐coated carboxy MNPs across three batches. The magnetic cores used in this process are well‐established 200 nm products from the company (Figure S10). As shown in Figure 2c, the MNPs size distributions are highly consistent among three independent batches after silica coating (tetraethyl orthosilicate), and predominantly centered around 390, 600, and 950 nm with different reaction times. The Zeta potential analysis in Figure 2d also shows the uniformity of surface functional group modifications using 390 nm MNPs as a model. The Zeta potentials in different density are: −17 ± 1 mV (mono‐carboxyl groups modified with succinic anhydride), −20 ± 1 mV (di‐carboxyl groups modified with 3‐(2‐Aminoethylamino) propyltrimethoxysilane), and −40 ± 1 mV (poly‐carboxyl groups modified with (3‐Glycidyloxypropyl) trimethoxysilane) (Table S4). These results show high consistency of both silica coating and functional group modifications performed by the system.

Batch‐to‐batch consistency of automated NAs extraction modules coupled with PCR verification is subsequently investigated using commercial MNPs‐based viral NAs extraction kits, SARS‐CoV‐2 pseudovirus samples, and three‐component PCR kits. As shown in Figure 2e and Table S5, Ct values are highly consistent across tests when NAs extraction is performed independently in triplicate toward samples containing pseudovirus with different concentrations (10^3^ copies/mL, 10^4^ copies/mL, and 10^5^ copies/mL). Additionally, temporal stability is also assessed by testing SARS‐CoV‐2 pseudovirus samples with a concentration of 10^5^ copies/mL using different extraction reagent systems (representing different Ct values) over five consecutive days, which also exhibits high reproducibility (Figure 2f; Table S6). These results together demonstrate the reliability of the system in automated NAs extraction and PCR verification.

To benchmark the overall performance of the autonomous system, Figure 2g compares its efficiency and consistency to Human‐in‐the‐Loop (HITL) experiments according to 1‐σ values, in which σ is the standard deviation of Ct values across batches. Five independent experimental schemes for silica coating and carboxyl modification of MNPs and their performances in NAs extraction are executed in parallel by the system and manual operations (Table S7). The autonomous system completes each scheme within 12 h, achieving a consistency score of ≥ 0.9 across all independent replicates. In contrast, HITL experiments require more than 45 h per scheme, with consistency scores ranging from 0.5 to 0.7 (Table S8). These results highlight the significant advantages of the AP‐Lab in terms of efficiency and reproducibility, demonstrating its superiority in optimizing pilot‐scale processes compared to manual approaches. Collectively, the results in Figure 2 underscore the exceptional stability, consistency, and efficiency of the autonomous system in reagent preparation, MNPs coating and modification, NAs extraction, and PCR validation. These unique characteristics as compared with traditional manual operation, may provide a robust solution for pilot‐scale MNPs‐based NAs extraction products development.

### 2.3 Optimization and Scale‐Up

Given the high reliability and efficiency of the autonomous system within the AP‐Lab, we subsequently conduct extensive iterative optimization of MNPs surface modification and reagents matching in NAs extraction and detection for three viruses (SARS‐CoV‐2, influenza virus A, and influenza virus B) at pilot‐scale. Optimization speed and outcomes using industrial datasets (Tables S9 and S10) versus publicly available scientific literature datasets are first compared to investigate the importance of localized industrial datasets in product development. At the beginning of the optimization iterations, the initial input instructions for the AP‐Lab are as follows: “*perform iterative optimization for viral NAs extraction MNPs, calculate individual weighting factors for each sub‐grouped “Condition”, and provide the optimal products and formulation ratios to achieve a PCR Ct value below 26.5 at a fixing viral NAs concentration of 10^5^ copies/mL*”. At this concentration, achieving a Ct value of approximately 26 corresponds to an extraction efficiency exceeding 99% (Figure S11).

**FIGURE 3:**
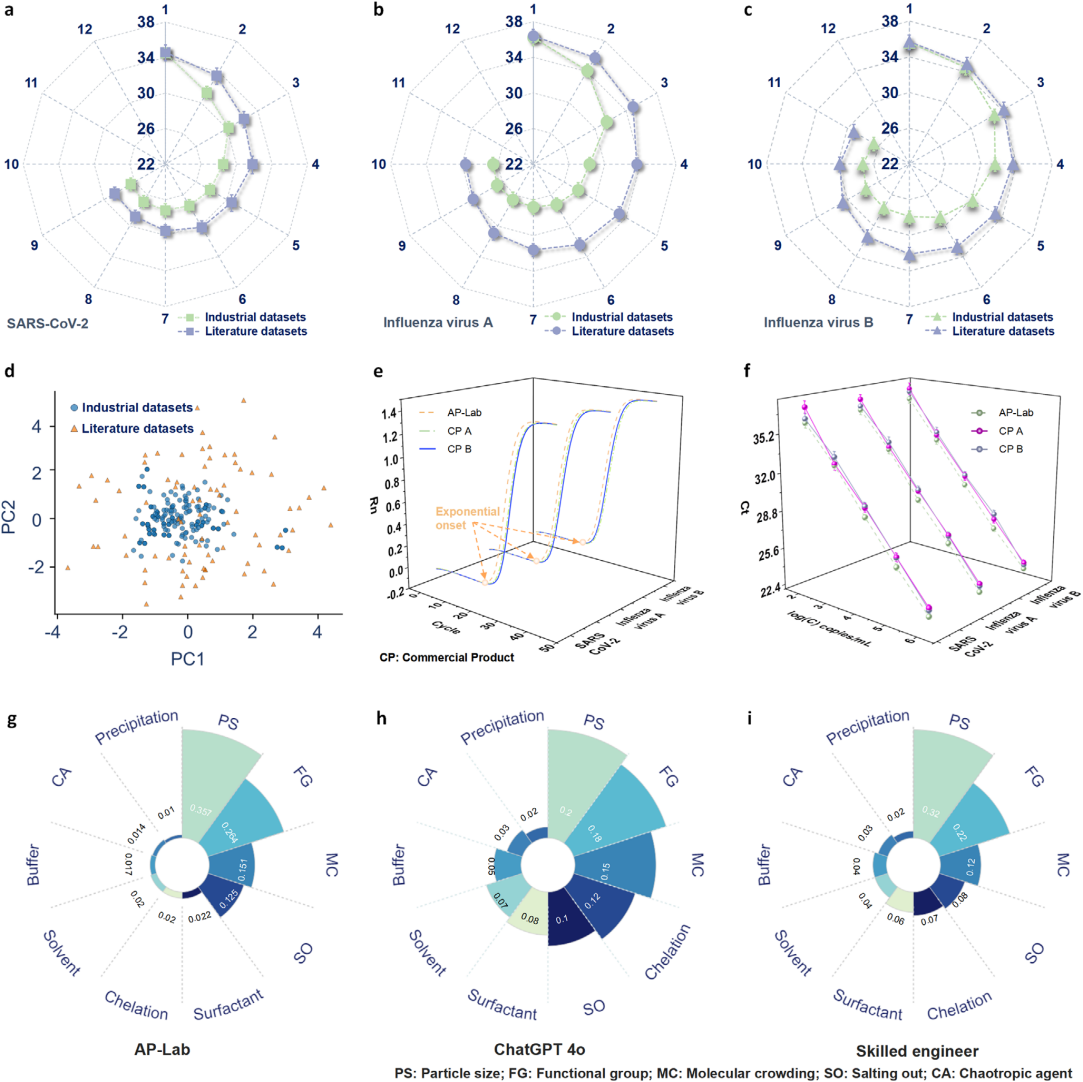
Optimization performance and comparative evaluation of the MNPs‐based NAs extraction products. (a–c) Iterative optimization results for the MNPs‐based NAs extraction products targeting SARS‐CoV‐2 in (a), influenza virus A in (b), and influenza virus B in (c). Using proprietary industrial datasets, the AP‐Lab reduces Ct values from 35.5 to 26.02 (SARS‐CoV‐2, 9 iterations in (a), 36.6 to 26.4 (influenza virus A, 10 iterations in (b), and 36.2 to 26.1 (influenza virus B, 11 iterations in (c) (green dots). In contrast, optimizations based on publicly available scientific literature datasets show significantly slower progress, with final Ct values of 28.4, 29.8, and 28.8, respectively (blue‐gray dots). (d) PCA of the 10D feature space shows that industrial datasets cluster in a compact “manufacturable” region, while scientific literature datasets are sparse and widely dispersed. (e) Representative PCR curves for three viruses comparing the AP‐Lab optimized MNPs‐based NAs extraction products with two leading commercial products, showing lower Ct values for the AP‐Lab, indicative of superior extraction performance. (f) Comparative performance testing of the AP‐Lab optimized MNPs‐based NAs extraction products against leading commercial products, showing superior linearity and lower LOD. (g–i) Analysis of parameter weight factors in the optimized SARS‐CoV‐2 MNPs‐based NAs extraction products. The AP‐Lab identifies particle size (0.357) and functional group (0.264) as key factors in (g), outperforming ChatGPT 4o, which produced more uniform weight distributions in (h), and skilled engineers, whose estimates lacked precision in (i). These differences arise because the AP‐Lab provides data‐driven importance weights, ChatGPT‐4o provides text‐knowledge‐based heuristic weights, and human engineers provide experience‐based weights.

The pilot‐scale optimization (300 mL for MNPs coating and modification corresponding to 50,000 tests, and 3 L for NAs extraction reagents preparation corresponding to 10,000 tests) results for MNPs and corresponding extraction reagents used in NAs extraction for the three viruses are presented in Figure 3a–c, illustrating the best Ct values in each round of the iterative optimization process. For SARS‐CoV‐2, the Ct value is improved from an initial 35.5 to a final 26.02 after 9 iterations based on industrial datasets (Figure 3a, green dots; Supplementary Materials M1 containing Tables S11–S30 and Figures S12–S20). Meanwhile, comparative tests are conducted using publicly available scientific literature datasets, where the Ct value is only improved from 35.5 to 28.4 over 9 iterations (blue‐gray dots). This comparison clearly demonstrates that the optimization iteration process based on scientific literature datasets is significantly slower than that using proprietary industry datasets. Similarly, for influenza virus A and B, the Ct value is improved from 36.6 to 26.4 (Figure 3b, green dots; 10 round iterations) and 36.2 to 26.1 (Figure 3c, green dots; 11 round iterations) based on industrial datasets, respectively. In contrast, when using scientific literature datasets, the Ct value is improved only from 36.6 to 29.8 for influenza virus A (Figure 3b, blue‐gray dots) and from 36.2 to 28.8 for influenza virus B (Figure 3c, blue‐gray dots). These phenomena further confirm the importance of industrial datasets in accelerating the speed and outcomes of product optimization. Principal component analysis (PCA) of the 10D feature space (Figure 3d; Figure S21) further reveals that industrial datasets are concentrated within a compact “manufacturable” region, whereas scientific literature datasets are more sparsely distributed over a broader parameter space. As a result, optimization trajectories initialized from industrial datasets converge toward low Ct regions in the feature space more rapidly, while literature‐based trajectories often take longer, less directed paths across parameter combinations that are theoretically interesting but practically suboptimal for robust pilot‐scale performance. Meanwhile, the optimization process based on the AP‐Lab takes only three weeks. In contrast, traditional manual methods for equivalent pilot‐scale development typically require four to six months due to labor‐intensive operations, lower throughput, inconsistent reproducibility, and inefficient parameter analysis in manual optimization. These results underscore the high efficiency and precision‐driven performance of the AP‐Lab in streamlining pilot‐scale MNPs‐based NAs extraction products development.

The optimized MNPs‐based NAs extraction products at pilot‐scale are further evaluated against the leading commercial products (Supplementary Materials M2 and M3) as shown in Figure 3e. The viral samples are prepared at a concentration of 10^5^ copies/mL, and comparative tests are conducted using the recommended methods according to the manufacturer's instructions (Supplementary Materials M4). Results demonstrate that for all three types of MNPs‐based NAs extraction products developed by the AP‐Lab, the exponential rise in the PCR amplification curves occurs earlier than for the current leading commercial products. Moreover, the AP‐Lab optimized products also exhibit superior linearity and lower LOD (Limit of Detection) across a range of viral concentrations for three viruses compared with the current leading commercial products (Figure 3f and Table 1 for SARS‐CoV‐2). Together, considering the development timeline, the AP‐Lab enables a rapid transition from laboratory formulations to pilot‐scale products with stable and superior performances.

**TABLE 1:** Comparison of LOD performance between optimized SARS‐CoV‐2 detection reagents and commercial products.

Sample	AP‐Lab	Commercial product A	Commercial product B
	Ct value	Ct value	Ct value
Negative Control	N/A	N/A	N/A
‌Positive Control (2×10^2^ copies/mL)	35.85	N/A	N/A
Positive Control (5×10^2^ copies/mL)	35.26	35.53	35.46
Positive Control (10^3^ copies/mL)	33.12	33.36	33.45
Positive Control (10^4^ copies/mL)	29.42	29.68	29.63
Positive Control (10^5^ copies/mL)	26.06	26.39	26.48

To investigate the parameter influences in the final optimized formulations and to further guide scale‐up production, the weighting factors of “*Conditions*” for SARS‐CoV‐2 MNPs‐based NAs extraction products are thoroughly analyzed, as depicted in Figure 3g. Particle size (weight factor 0.357) and functional group (weight factor 0.264) emerge as the most influential parameters for MNPs, and molecular crowding (weight factor 0.151) and salting out (weight factor 0.125) as the most influential parameters for extraction reagents. As compared against the weighting factors estimates provided by the high‐performing general LLM ChatGPT 4o, ChatGPT 4o correctly identifies particle size as the most important parameter and functional group as the second most important, but it fails to provide distinct differences between the influence magnitudes of these parameters, resulting in a more uniform distribution of weights (Figure 3h). This inevitably increases complexities in subsequent multi‐dimensional optimization processes. In Figure 3i, skilled engineers can provide weight distributions for various factors that are similar to those determined by the AP‐Lab after examining rounds of iterative results. However, the range and precision of these weight distributions differ significantly from those generated by the AP‐Lab. This difference among the three approaches arises because the AP‐Lab provides data‐driven importance weights, ChatGPT 4o represents text‐knowledge‐based heuristic weights, and engineers provide experience‐based weights. The AP‐Lab infers parameter importance directly from closed‐loop experimental data, rather than relying on purely text‐based priors or human heuristics. By continuously updating the weighting factors from iterative ML training, the AP‐Lab produces numerically sharper and more differentiated importance profiles than those obtained from ChatGPT‐4o or skilled engineers. This sharper discrimination allows the optimization loop to allocate experimental resources more efficiently to the most influential “*Conditions*” in subsequent iterations. These results demonstrate the unique capability of the AP‐Lab strategy in analyzing and optimizing multi‐dimensional parameters through iterative testing and ML‐driven evaluations, hence greatly accelerating the pilot‐scale development of material products with unparalleled efficiency and precision.

**FIGURE 4:**
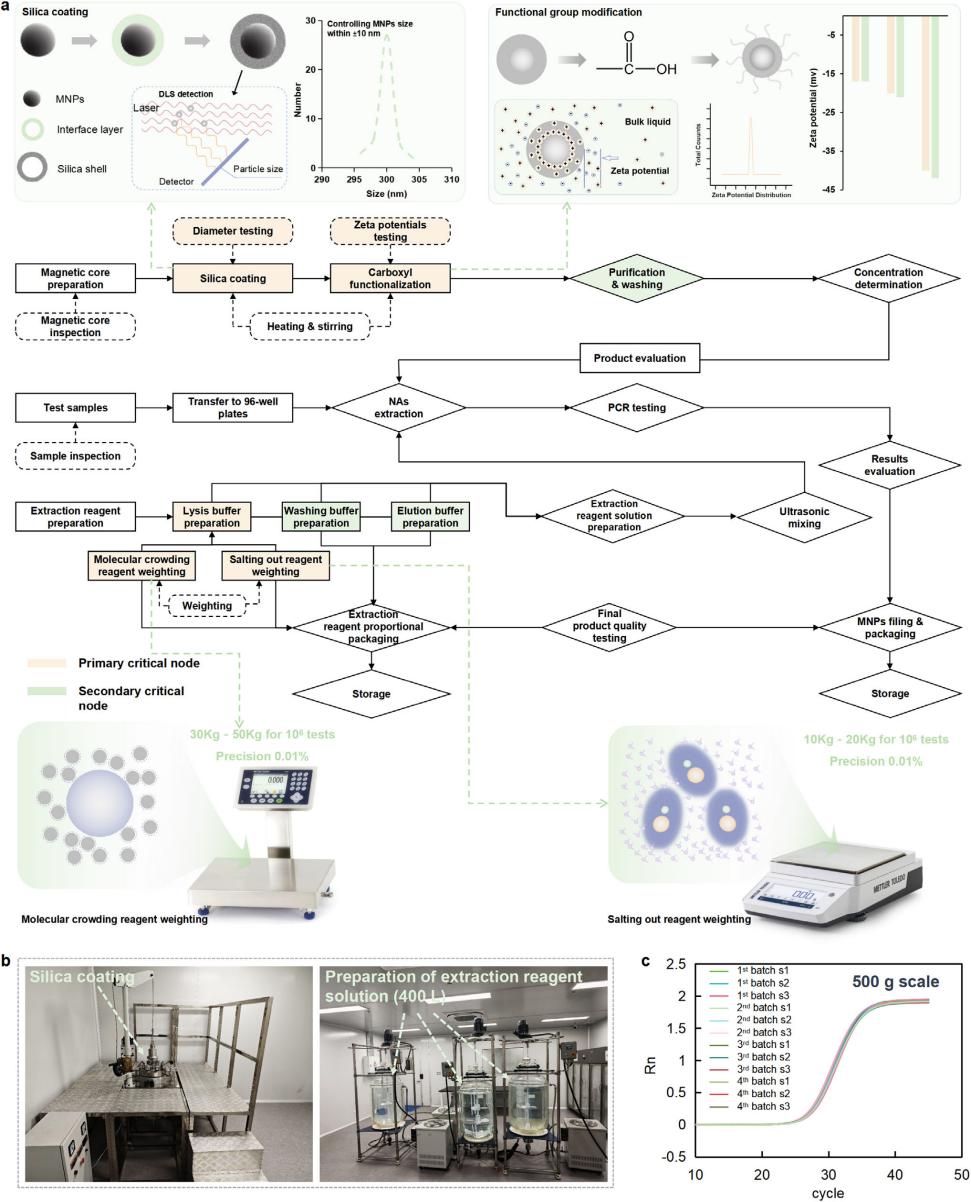
Scale‐up production and key process nodes for MNPs‐based NAs extraction products development using the AP‐Lab. (a) Workflow for the 500 g scale‐up carboxyl modified MNPs production and 400 L NAs extraction reagents preparation, highlighting primary critical nodes (orange) such as silica coating and carboxyl functionalization, and secondary critical nodes (green) such as purification & washing. These nodes are essential for ensuring process consistency and product reliability. The primary nodes are also schematically illustrated, including rigorously controlled MNPs size distributions (monitored by DLS), functionalization quantified via Zeta potential, and precise weighting of molecular crowding and salting out reagents. (b) Images of the scale‐up production equipment. (c) Batch‐to‐batch consistency testing for four production batches, each with three experimental groups, showing uniform PCR performance across all batches.

Scale‐up production while maintaining consistency and performance is one of the most important quality control factors in validating the effectiveness of pilot‐scale development. To evaluate the scalability of the AP‐Lab, a production line for 500 g per batch MNPs modification and 400 L reagents preparation corresponding to 1 million tests is implemented and tested according to the experimental protocols and key process nodes identified by the AP‐Lab. Particularly, key process nodes are categorized into primary critical nodes and secondary critical nodes, as defined by the AP‐Lab. Figure 4a illustrates the complete workflow for scale‐up production, highlighting these critical nodes and their roles in ensuring process reliability and product consistency. The scale‐up process begins with magnetic core preparation previously established. A magnetic core modification line is setup by following protocols and optimal parameters determined by the AP‐Lab. In this line, silica coating (diameters determined by Dynamic Light Scattering, DLS) and carboxyl functionalization (Zeta potentials determined by DLS) are primary critical nodes due to their significant impact on the product performance (highlighted in orange), while purification and washing are secondary critical nodes (highlighted in green). In the NAs extraction reagents preparation, molecular crowding and salting out reagents (determined by weighting) in lysis buffer are identified as primary critical nodes, also marked in orange in Figure 4a, while secondary important nodes such as washing buffer and elution buffer, are marked in green. For example, after silica coating, particle size distributions are rigorously monitored by DLS to ensure that the MNPs remain tightly confined around the intended target diameters. Subsequent functional group modification is quantified via Zeta potential, which serves as a sensitive proxy for surface functional group density and its batch‐to‐batch reproducibility. In addition, molecular crowding reagents and salting out reagents, identified as high‐impact formulation components, are subject to precise weighting control (0.01% precision), ensuring that their contributions to NAs extraction performance remain tightly constrained during scale‐up.

To validate the scale‐up production, the AP‐Lab provides detailed experimental protocols for each critical node, emphasizing parameter optimization and consistency. These protocols are executed using equipment designed for 500 g scale‐up carboxyl modified MNPs production and 160 L jacketed reactors for 400 L NAs extraction reagents preparation as shown in Figure 4b. The scale‐up production is evaluated through a series of batch‐to‐batch consistency tests. Four production batches are prepared, with each batch divided into three experimental groups for NAs extraction and PCR performance testing. The results, presented in Figure 4c, demonstrate remarkable consistency across batches. The PCR curves and Ct values of the MNPs‐based NAs extraction products at a scale of 1 million tests per batch are highly uniform, confirming that the scale‐up process preserves the performance characteristics achieved during the earlier pilot‐scale optimization. This achievement underscores the robustness of the AP‐Lab in accelerating the pilot‐scale development and transferring scale‐up production of high‐performance products, establishing a reliable pathway for transitioning laboratory‐scale innovations to industry‐scale applications.

In contrast to existing autonomous laboratories that mainly accelerate laboratory‐scale discovery, the AP‐Lab is purpose‐built for pilot‐scale, application‐oriented product development (Table 2). It integrates proprietary industrial datasets with closed‐loop optimization on a high‐throughput autonomous system, and is directly benchmarked by an application‐level figure of merit, the PCR Ct values for NAs extraction, which holistically capture optimal MNPs quality and reagent formulations. Because Ct is a functional metric that more faithfully reflects end‐use performance than individual physicochemical descriptors (e.g., particle size or Zeta potential), it provides a high‐fidelity learning signal when combined with realistic industrial datasets, allowing ML models to pinpoint truly impactful parameters and output recommendations that can be translated to production facilely.

**TABLE 2:** The comparison of typical autonomous laboratories and the AP‐Lab.

Platform /system	Primary domain and goal	Typical experimental scale	Main optimization benchmark	Data basis	Relevance and limitation for pilot‐scale product development	Representative refs
AP‐Lab	Pilot‐scale optimization of MNPs‐NAs extraction systems, transition from laboratory recipes to market‐ready products	10–500 g, ∼10^6^ tests per batch	Application‐oriented benchmark Ct (NAs extraction and PCR performance)	Industrial data and iterative high‐throughput data	Directly targets market‐ready performance and scale‐up	This work
A‐Lab	Discovery of new inorganic and solid‐state materials	mg‐g pellets	Phase formation, structural, and thermodynamic stability	Historical data from the literature	Powerful for discovery and synthesis; no explicit pilot‐scale translation	Szymanski et al., Nature 2023 [14]
Mobile Robotic Chemist	Photocatalysts discovery	mg and µmol	Photocatalytic activity under defined illumination	In‐laboratory experimental data acquired online	Efficient exploration; not coupled to manufacturing scale	Burger et al., Nature 2020 [38]
Self‐Driving Lab “Ada”	Thin film materials discovery	µL‐mL scale	Electronic, optical properties	Experimental thin film data	Maps laboratory‐scale Pareto fronts; lacks downstream product integration	MacLeod et al., Sci. Adv. 2020 [39]
Chemputer & Digital Platforms	General purpose automated organic synthesis and reaction optimization	mmol scale	Yield, purity, route generality	Encoded reaction procedures and in situ analytical data	Standardizes synthesis; limited link to end‐use assay performance	Steiner et al., Science 2019 [15]; Jiang et al., Sci. Adv. 2022 [40]
Artificial Chemist	Metal‐halide perovskite quantum dots, tune bandgap and optical quality in flow	µL‐mL per reaction, continuous microreactor	Photoluminescence quantum yield, emission peak energy​, emission linewidth​	Spectral data from autonomous flow experiments	Demonstrates closed‐loop ML, flow chemistry, and knowledge transfer	Epps et al., Adv. Mater. 2020 [41]
Autonomous Multiproperty Molecular Discovery Platform	Small molecule dyes, multi‐property molecular discovery across diverse scaffolds	µmol‐mmol scale	Three simultaneous properties: *λ*_max_, log*K*_ow_, and log*k*_deg_	Public molecular data, platform generated laboratory data	Integrates generative models, CASP, and automation in a general DMTA loop, but targets molecular discovery rather than pilot‐scale manufacturing	Koscher et al., Science 2023 [42]
Self‐Driving Adhesive Lab	Formulated materials optimization	g scale	Bond strength	Experimental data	Demonstrates formulation optimization; lacks pilot‐scale, industrial datasets coupling and mass‐production validation	Rooney et al., Digital Discovery 2022 [43]
Cloud Labs (MAOSIC‐Type Systems)	Autonomous materials synthesis via cloud collaboration	µL‐mL scale	Optical, activity signals	Scientific and exploratory data	Demonstrates powerful closed‐loop optimization, but remains laboratory‐scale and discovery‐oriented	Li et al., Nat. Commun. 2020 [44]
AMANDA Line One	Organic photovoltaic devices, high‐throughput co‐optimization of efficiency and photostability	mg scale	Device level power conversion efficiency and photostability	Closed, platform generated experimental data	Highly relevant for multi‐parameter device, process optimization, and stability studies, but remains laboratory‐scale	Du et al., Joule 2021 [45]

MNPs and NAs extraction reagents are central to modern molecular diagnostics. By leveraging industrial datasets and AP‐Lab's rapid closed‐loop optimization, the development cycle for MNPs‐based NAs extraction products can be substantially shortened. For more complex scenarios, factors such as sample type and matrix inhibitors may need to be included as additional optimization dimensions beyond the current ten “*Conditions*,” and the Ct objective can be extended from a single‐matrix target to robustness‐oriented benchmarks across multiple matrices [28]. More broadly, a similar framework can be generalized to other MNPs‐based diagnostic products [29, 30], for example, immunoassay kits [31, 32] or sequencing sample‐prep kits [33], by defining clinically meaningful performance metrics and optimizing the associated formulation and process variables. Additionally, the AP‐Lab is also applicable beyond MNPs. In principle, material products with well‐defined industrial metrics and application‐oriented benchmarks can benefit from similar frameworks, such as heterogeneous catalysts [34, 35], and advanced battery materials [36, 37].

In summary, we develop and demonstrate the AP‐Lab, an advanced AI‐driven autonomous workstation designed to optimize and standardize the synthesis of MNPs and NAs extraction reagents system at pilot‐scale. By integrating proprietary industrial datasets and using Ct values as the final application‐oriented benchmarks, pilot‐scale MNPs‐based NAs extraction products superior to commercial products are developed within three weeks, and a scale‐up manufacturing line for its mass production corresponding to 1 million tests per batch is further established within two months. Such progresses demonstrate the great potential of AI‐driven strategies in manufacturing industry, and also highlight the importance of integrating customized industrial datasets and selecting appropriate benchmarks in accelerating materials and products development. Additionally, due to its modularity and adaptivity, the established AP‐Lab workstation may also be expanded and adapted in pilot‐scale productions for other micro‐ and nano‐materials‐based products beyond MNPs as discussed above, thus providing a potential solution in filling the growing gap between perceived progress and real‐world impact in AI‐driven techniques in the advanced materials manufacturing industry.

## Author Contributions

X.‐F.Y. and W.Z. conceived the concept. Z.W. led the establishment of AP‐Lab, with contributions from G.J., B.J., and W.S. W.X.S. and Z.Z. provided industrial data and initial magnetic core products. Z.M. and M.J. performed iterative experiments and evaluations using AP‐Lab. Z.W., G.J., B.W., and Z.Y.W. developed the machine learning algorithms and data processing workflows. Z.W. collected the data and drafted the manuscript. W.Z. and X.‐F.Y. edited the manuscript. All authors discussed the results and commented on the manuscript. S.G., Z.Z., W.Z., and X.‐F.Y. supervised the project.

## Conflicts of Interest

The authors declare no conflicts of interest.

## Supporting information




**Supporting File**: advs74293‐sup‐0001‐SuppMat.docx.

## Data Availability

The data that support the findings of this study are available from the corresponding author upon reasonable request.
