# Supplementary material for: AP‐Lab: An AI‐Driven Autonomous Pilot‐Scale Platform Bridging Materials Discovery and Industrial Manufacturing
Source: Adv Sci (Weinh). 2026 Feb 12;13(22):e74293. doi: 10.1002/advs.74293 (PMC13088262; doi:10.1002/advs.74293)
Supplement: Supplementary file 1 — Supporting File: advs74293‐sup‐0001‐SuppMat.docx. [file ADVS-13-e74293-s001.docx]

***Supplementary Materials***

**AP-Lab: An AI-Driven Autonomous Pilot-Scale Platform Bridging Materials Discovery and Industrial Manufacturing**

Zhan-Long Wang^1,4,#^, Zhifen Ma^1,#^, Wenxing Song^2,#^, Guolai Jiang^1,4^, Boshi Jiang^1,4^, Mingyang Jiang^1^, Weiliang Shu^1,4^, Bing Wang^1^, Zhiyuan Wan^1^, Shengyong Geng^1,*^, Zhen Zhao^1,2,4,*^, Wenhua Zhou^1,2,3,4,*^, Xue-Feng Yu^1,3,4,*^

^1^ Center for Materials Artificial Intelligence, Shenzhen Institutes of Advanced Technology, Chinese Academy of Sciences, Shenzhen, Guangdong, 518055, China

^2^ Shenzhen Archean Biotechnology Co., Ltd, Shenzhen, Guangdong, 518107, China

^3^ Key Laboratory of Biomedical Imaging Science and System, Chinese Academy of Sciences, Shenzhen, Guangdong, 518055, China

^4^ Shenzhen Small and Medium-sized Pilot Base for AI-driven Material Creation and Manufacture, Shenzhen, Guangdong, 518055, China

^#^ These authors contributed equally: Zhan-Long Wang, Zhifen Ma and Wenxing Song

^*^Authors to whom correspondence should be addressed: [sy.geng@siat.ac.cn](mailto:sy.geng@siat.ac.cn), [zhen.zhao1@siat.ac.cn](mailto:zhen.zhao1@siat.ac.cn), [wh.zhou@siat.ac.cn](mailto:wh.zhou@siat.ac.cn), [xf.yu@siat.ac.cn](mailto:xf.yu@siat.ac.cn)

**Contents**

[1. Supplementary Materials Table S1: Data preprocessing and feature structuring for machine learning (ML) 4](#_Toc218244748)

[2. Supplementary Materials Figure S1: The user interface (UI) 7](#_Toc218244749)

[3. Supplementary Materials Figure S2 and S3: ML-based closed-loop optimization workflow in the AP-Lab 9](#_Toc218244750)

[4. Supplementary Materials Figure S4: Performance comparison of ML models and LLM-assisted optimization. 12](#_Toc218244751)

[5. Supplementary Materials Figure S5: Effect of historical data usage on model accuracy and overfitting. 13](#_Toc218244752)

[6. Supplementary Materials Figure S6: Detailed structures of artificial island of autonomous synthesis and testing system 14](#_Toc218244753)

[7. Supplementary Materials Figure S7: The quantitative module of autonomous synthesis and testing system 15](#_Toc218244754)

[8. Supplementary Materials Figure S8: 3L large-capacity cleaning and dispersing module 16](#_Toc218244755)

[9. Supplementary Materials Figure S9: The 12-channel multifunctional module 17](#_Toc218244756)

[10. Supplementary Materials Table S2: Detailed data of solid reagents feeding 18](#_Toc218244757)

[11. Supplementary Materials Table S3: Detailed data of liquid reagents feeding 19](#_Toc218244758)

[12. Supplementary Materials Figure S10: The Scanning Electron Microscope (SEM) images of magnetic cores and silica-coated MNPs 20](#_Toc218244759)

[13. Supplementary Materials Table S4: The Zeta potentials of silica-coated MNPs. 21](#_Toc218244760)

[14. Supplementary Materials Table S5: PCR batch-to-batch consistency testing results 22](#_Toc218244761)

[15. Supplementary Materials Table S6: PCR time-scale consistency testing results 23](#_Toc218244762)

[16. Supplementary Materials Table S7: Five-batch experimental testing schemes and PCR testing results 24](#_Toc218244763)

[17. Supplementary Materials Table S8: The comparison of PCR experimental results between the AP-Lab and HITL 25](#_Toc218244764)

[18. Supplementary Materials Table S9 and S10: Representative industrial datasets 27](#_Toc218244765)

[19. Supplementary Materials Figure S11: The relationship between virus concentrations and Ct values 35](#_Toc218244766)

[20. Supplementary Materials M1: Detailed optimization schemes and results of each iteration for SARS-CoV-2 detection 36](#_Toc218244767)

[21. Supplementary Materials Figure S21: Quantifying coverage and sparsity of industrial vs literature datasets. 72](#_Toc218244768)

[22. Supplementary Materials M2: The detailed information of commercial products A (DAAN), NAs extraction and purification kit instruction manual 73](#_Toc218244769)

[23. Supplementary Materials M3: The detailed information of commercial products B (Thermo Fisher), NAs extraction and purification reagent manual 80](#_Toc218244770)

[24. Supplementary Materials M4: Testing methods 82](#_Toc218244771)

1. Supplementary Materials Table S1: Data preprocessing and feature structuring for machine learning (ML)

The datasets are initially classified into “***conditions*”** based on the functional roles. For reagent-related conditions, each “***condition*”** is further divided into “***sub-conditions*”** according to the specific types of reagents used. The sources of reagent “***sub-conditions*”** come from two categories:

1. Reagents used in experimental protocols from initial **industrial datasets**;
2. **Reagent recommendations generated by large language models (LLM)**.

**Table S1** presents the initial data from industrial datasets, primarily based on the reagent types used in the pilot-scale experiments. For each reagent-related condition, we reserve **eight parameter slots** for commonly used reagent types, and for the LLM-recommended reagents, placeholder slots are left for future integration.

For non-reagent conditions:

1. **Particle size** is treated as a **numerical feature**, and its sub-condition remains as particle size.
2. **Functional groups** are categorized as mono-carboxyl (represented as **1**), di-carboxyl (**2**), and poly-carboxyl (**3**).

A total of **ten** “***conditions*” types** is defined, encompassing **66** “***sub-conditions*”** in total. For reagent-related “***conditions*”**, the attribute of each “***sub-condition*”** is the **mass** (e.g., 0.5 g). The eight “***sub-conditions*”** reserved under each reagent condition are selected to cover the most commonly used reagents in practice. Rare or extremely uncommon reagents are not considered in the datasets. Reagents recommended by the LLM are also limited to commonly used ones. During ML model training, a **66-dimensional parameter vector** is used as the input feature set. For reagent types not currently available or included, the corresponding parameter values are set to **zero**.

Table S1. Data preprocessing and feature structuring for ML

| Conditions | Sub-conditions | Properties | Example values |
| --- | --- | --- | --- |
| particle size | Particle size | Size (nm) | 600 nm |
| Surfactant | SDS (Sodium Dodecyl Sulfate) | Mass (g) | 0.2 g |
|  | Triton X-100 | Mass (g) | 0.2 g |
|  | Tween 20 | Mass (g) | 0.2 g |
|  | …… |  |  |
| Salting out reagent | NaCl | Mass (g) | 0.5 g |
|  | (NH₄)₂SO₄ | Mass (g) | 0.5 g |
|  | K₂SO₄ | Mass (g) | 0.5 g |
|  | …… |  |  |
| Buffer | Tris-HCl | Mass (g) | 0.5 g |
|  | PBS | Mass (g) | 0.5 g |
|  | HEPES | Mass (g) | 0.5 g |
|  | …… |  |  |
| Functional group | Functional group | Density (arbitrary) | 1, 2, 3 |
| Chelation reagent | EDTA | Mass (g) | 0.3 g |
|  | EGTA | Mass (g) | 0.3 g |
|  | DTPA | Mass (g) | 0.3 g |
|  | …… |  |  |
| Molecular crowding reagent | PEG-2000 | Mass (g) | 0.4 g |
|  | Ficoll | Mass (g) | 0.4 g |
|  | Dextran | Mass (g) | 0.4 g |
|  | …… |  |  |
| Precipitation reagent | Isopropanol | Mass (g) | 0.2 g |
|  | Ethanol | Mass (g) | 0.2 g |
|  | Ammonium Acetate | Mass (g) | 0.2 g |
|  | …… |  |  |
| Chaotropic reagent | Guanidine Hydrochloride | Mass (g) | 0.3 g |
|  | Guanidine Thiocyanate | Mass (g) | 0.3 g |
|  | Potassium thiocyanate | Mass (g) | 0.3 g |
|  | ....... |  |  |
| Organic solvent | Phenol | Mass (g) | 0.2 g |
|  | Chloroform | Mass (g) | 0.2 g |
|  | DI Water | Mass (g) | 0.2 g |
|  | ...... |  |  |

1. Supplementary Materials Figure S1: The user interface (UI)

Figure S1 presents the UI of the AP-Lab workbench, designed to facilitate efficient management and control of the pilot-scale platform for materials development projects. The UI is organized into a navigation panel on the left and a central display area for project-specific information and operations. The left sidebar contains key navigation options, including “Home”, “Project”, “Device Cluster”, “Metadata”, and “User Settings”. These options allow users to access various sections of the AP-Lab, such as project management, equipment settings, data handling, and user configuration, providing a streamlined experience for managing multiple aspects of the system. At the top, a navigation path indicates the hierarchical structure within the workbench, showing that the user is accessing this project through the product model, cluster management, and metadata settings. Several tabs are displayed across the top of this section, including “Project Basic Information”, “Project Device Cluster Configuration”, “Project Element Configuration”, “Process Monitoring”, “Operation Log”, “Iteration Data”, and “Plan Output”. These tabs allow users to view and manage detailed information about the project, configure equipment and elements, monitor workflow, access logs, review data iterations, and output project plans. The main workspace in the central panel is divided into two sections: "Plan output" and "User input." The "Plan output" section provides a space for displaying generated project plans, while the "User input" section at the bottom allows users to input specific parameters or commands. This layout supports an interactive workflow, where users can input requirements and receive automated plans and recommendations from the AP-Lab workbench.


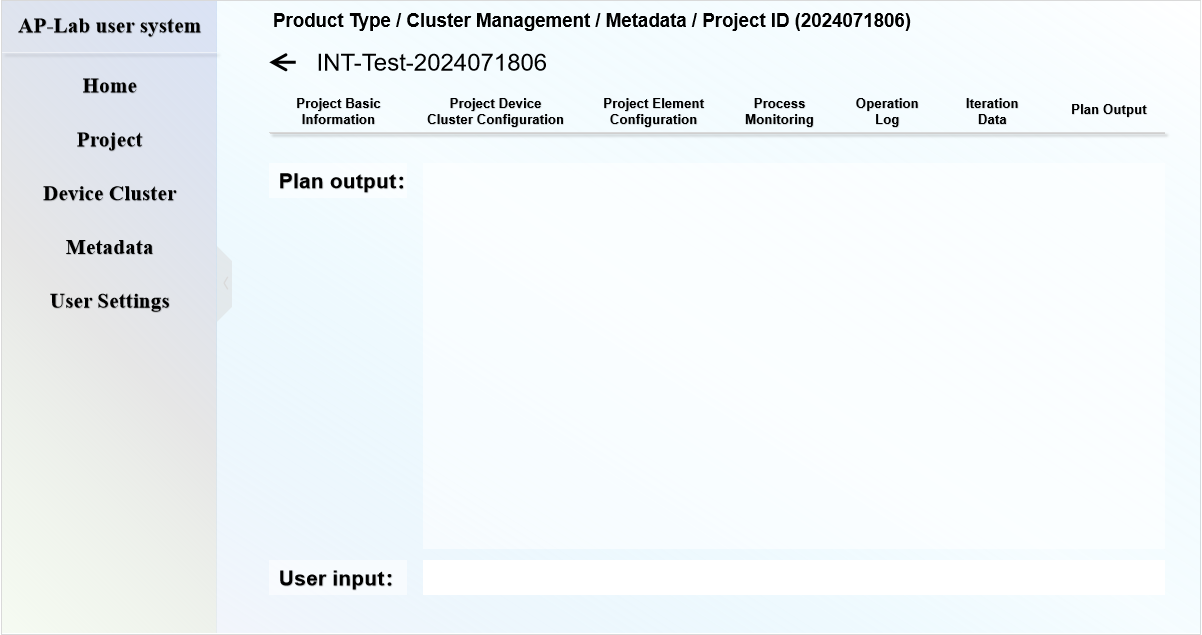


Figure S1. The UI of the AP-Lab for managing pilot-scale materials development projects. The interface includes a navigation panel on the left with options for accessing project management, equipment cluster settings, metadata, and user configurations. The central display area is focused on a specific project, with tabs for project information, equipment configuration, workflow monitoring, logs, iteration data, and plan output. The main workspace is divided into "Plan output" and "User input" sections, facilitating interactive datasets input and output within the AP-Lab.

1. Supplementary Materials Figure S2 and S3: ML-based closed-loop optimization workflow in the AP-Lab

We evaluate the integrated performance of ML algorithms and LLMs (Llama 3.1-8B) in accelerating the iterative optimization of pilot-scale magnetic nanoparticles (MNPs)-based nucleic acids (NAs) extraction systems. A weighted objective function approach is adopted for its intuitive implementation and practical interpretability. Figure S2 illustrates the core workflow of the optimization scheme generation system. The process begins with initial industrial datasets, followed by determining the optimal ML model and corresponding weight factors. These weight factors (WFₓ) reflect the contribution of each experimental parameter to the overall performance. Along with R² values and optimization algorithms, they guide the parameter selection process. Based on these, the system recommends 48 sets of sub-grouped “*Conditions*” combinations.

Once the sub-grouped “*Conditions*” combinations are selected, two scheme-generation strategies are evaluated side-by-side over the first few rounds to determine the better-performing approach. In one strategy, the ML module directly generated the executable reagent formulations, including the specific reagent parameters and corresponding execution schemes (Figure S2). In the other strategy, an LLM is used to translate the ML-selected parameter sets into detailed execution schemes (Figure S3). Before the full optimization campaign, a few preliminary rounds are conducted to benchmark the two strategies and identify the best-performing one.

These schemes are then executed autonomously on the autonomous synthesis and testing system, with real-time performance testing to evaluate experimental outputs. The system then either outputs a finalized optimal plan or proceeds to the next round of optimization based on updated data. This structured loop of model-driven parameter selection, LLM-assisted scheme generation, and automated execution forms a closed optimization cycle that efficiently refines formulations. The integration of LLM enables context-aware experiment generation, while the ML component ensures data-driven prioritization and evaluation. This combined framework enhances the speed and robustness of optimization, providing a scalable and intelligent solution for pilot-scale reagent development.


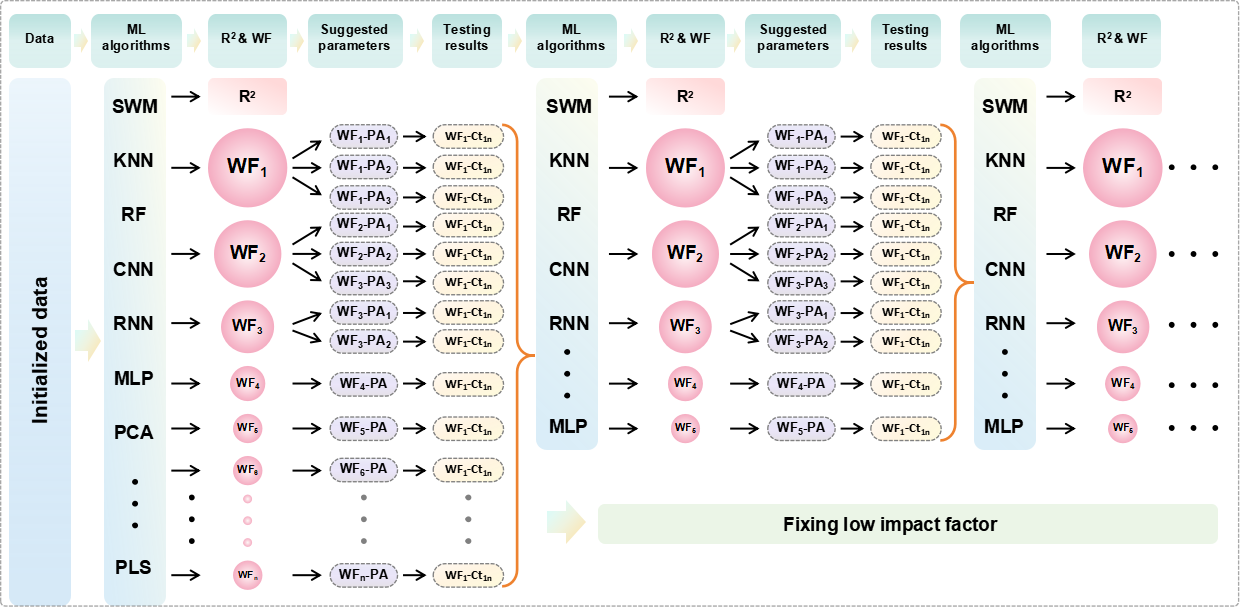


Figure S2. Schematic of the ML-driven optimization loop in the AP-Lab. Industrial datasets are first organized into ten condition subtypes and fed into a model library, where multiple regression models are trained and evaluated. The best-performing model (based on R²) yields weighting factors and suggests new parameter combinations. These suggested conditions are executed on the autonomous platform, producing testing results that are fed back into the ML library for iterative retraining and refinement.


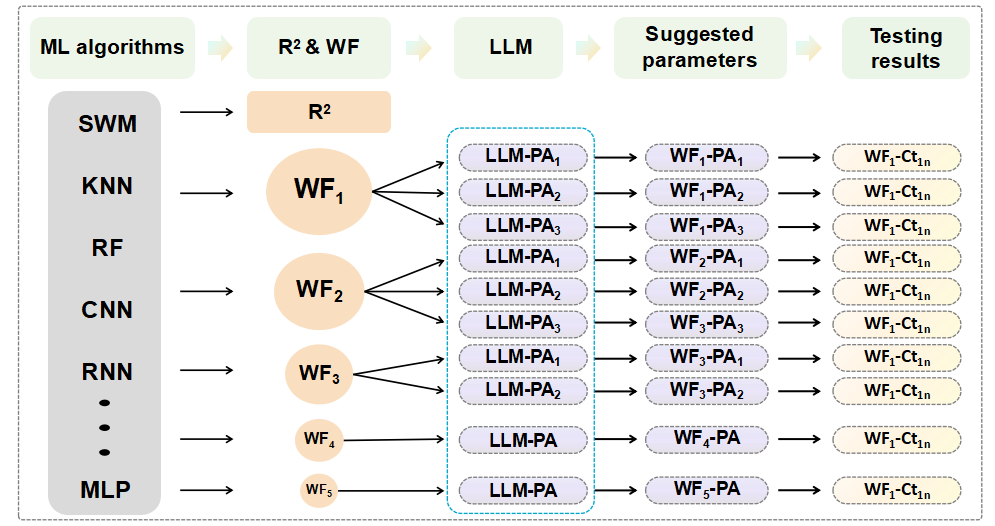


Figure S3. Schematic of the optimization loop when a LLM (Llama 3.1-8B) is integrated into the parameter recommendation stage. After ML models are trained and weighting factors are obtained, the LLM proposes concrete reagent types and composition ranges for high-impact parameters. These LLM-refined experimental schemes are encoded and executed on the autonomous platform. The resulting testing data are then returned to the ML-LLM hybrid loop for further training and iterative improvement.

1. Supplementary Materials Figure S4: Performance comparison of ML models and LLM-assisted optimization.


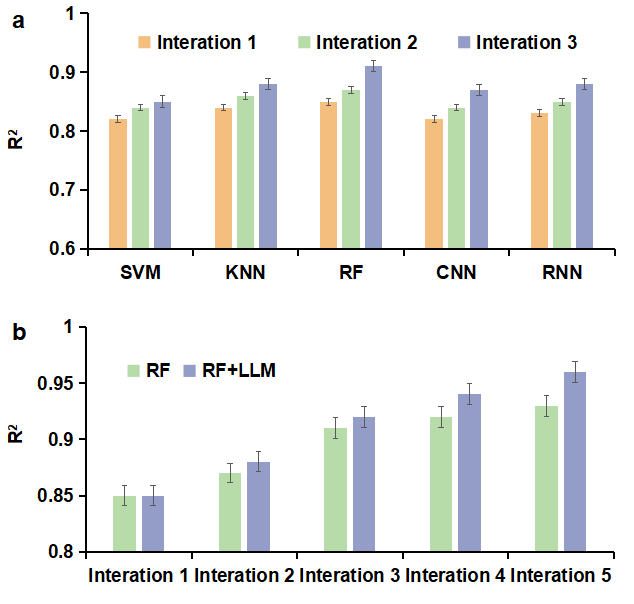


Figure S4. **a**, R² evolution over three optimization rounds for five best-performing models for MNPs-based NAs extraction systems, Support Vector Machine (SVM), k-Nearest Neighbors (KNN), Random Forest (RF), Convolutional Neural Network (CNN), and Recurrent Neural Network (RNN), trained on ten-dimensional feature-Ct pairs. RF consistently achieves the highest R² across iterations, and is therefore selected as the primary regression model for the MNPs-based NAs extraction systems. **b**, Comparison of RF performance with and without LLM assistance over five optimization rounds. The LLM-augmented workflow yields systematically higher R^2^ and faster convergence towards high-fidelity Ct prediction and formulation optimization.

1. Supplementary Materials Figure S5: Effect of historical data usage on model accuracy and overfitting.


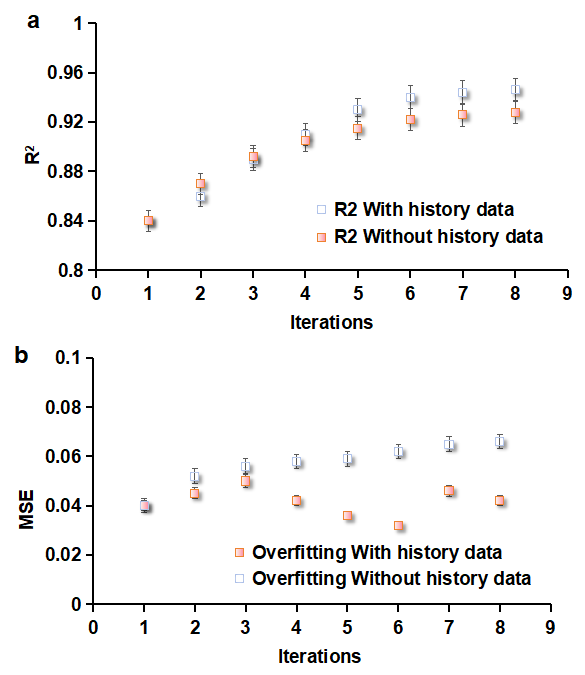


Figure S5. **a**, R^2^ evolution for RF models trained either with full historical data (all past iterations accumulated) or with only the latest iteration. Incorporating historical data leads to higher final R^2^ and faster improvement. **b**, Mean squared error (MSE) comparison illustrates reduced overfitting when historical data are retained, indicating that leveraging the complete iterative datasets improves generalization and stabilizes the optimization process in the AP-Lab workflow.

1. Supplementary Materials Figure S6: Detailed structures of artificial island of autonomous synthesis and testing system


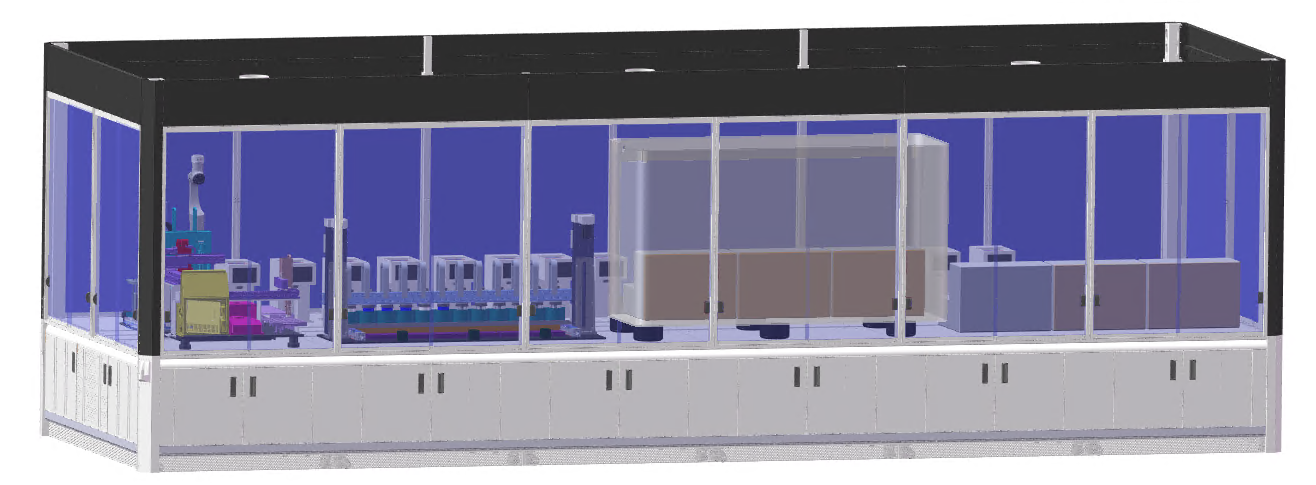


Figure S6. The structural diagram of the artificial island of autonomous synthesis and testing system.

1. Supplementary Materials Figure S7: The quantitative module of autonomous synthesis and testing system


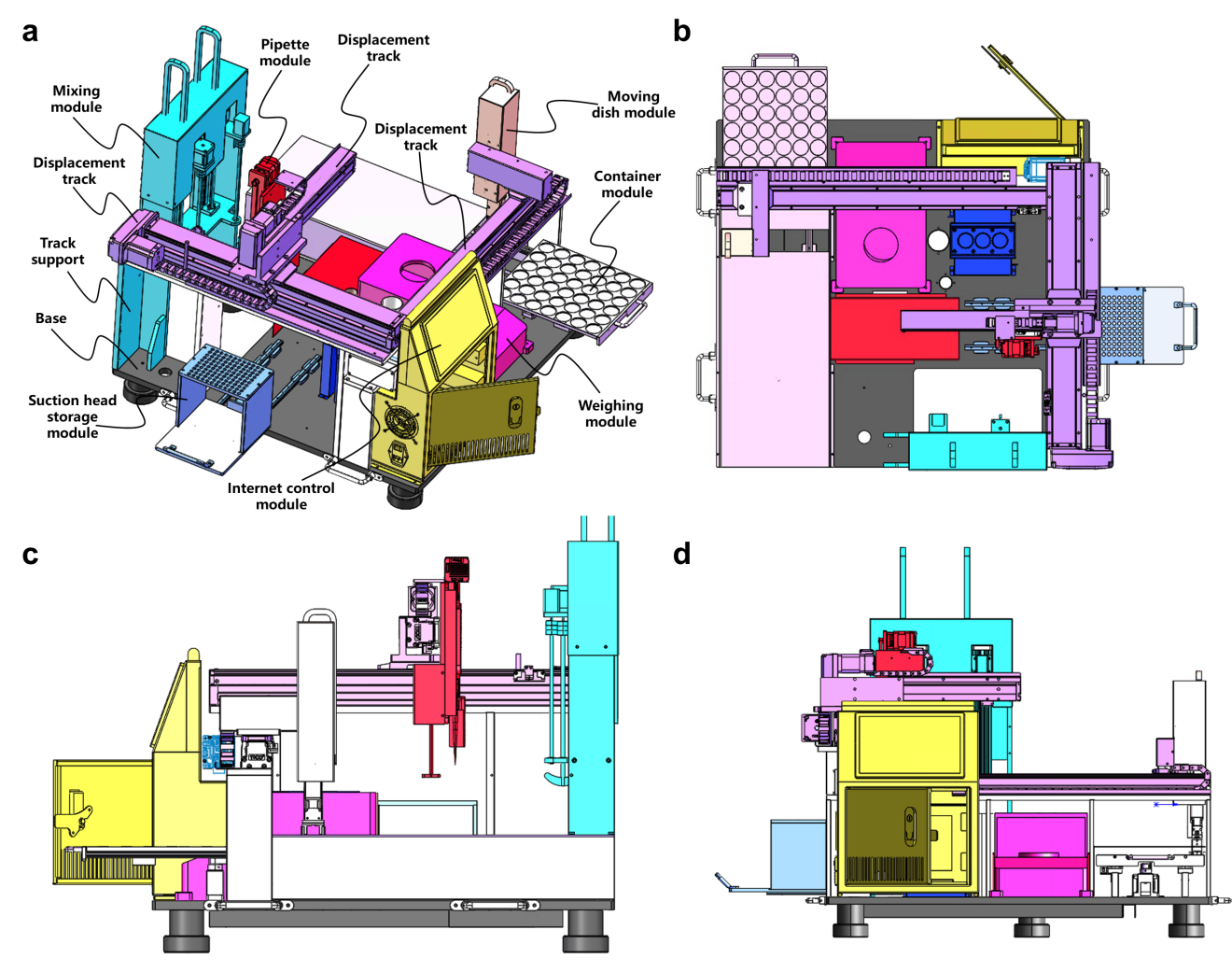


Figure S7. The quantitative module of autonomous synthesis and testing system. **a**, Schematic diagram of three-dimensional structure of the equipment. **b**, Top view of the structure. **c**, Side view of the structure. **d**, Front view of the structure.

1. Supplementary Materials Figure S8: 3L large-capacity cleaning and dispersing module


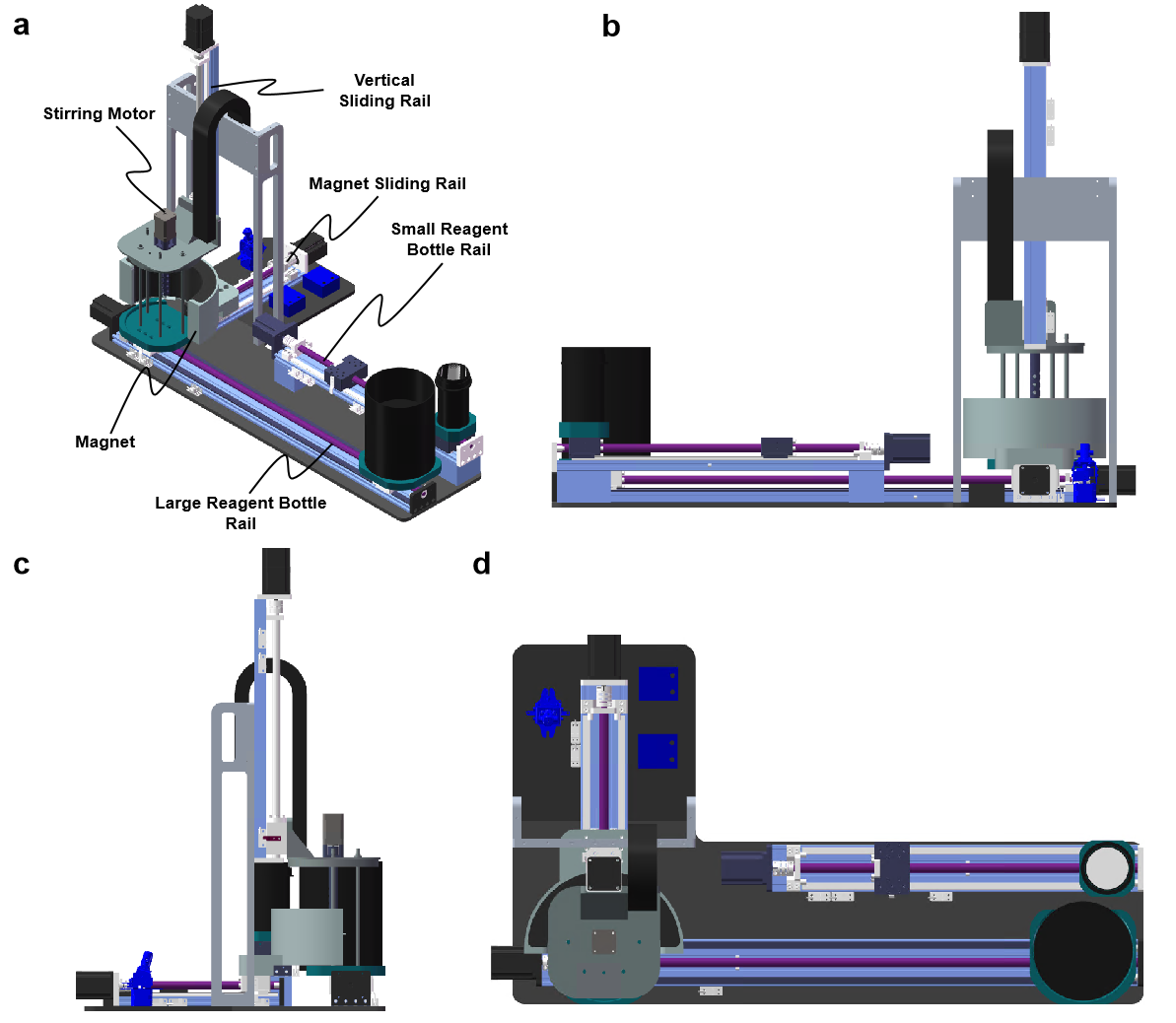


Figure S8. The structure diagram of the 3L large-capacity cleaning and dispersing module. **a**, Schematic diagram of three-dimensional structure of the equipment. **b**, Front view of the structure. **c**, Side view of the structure. **d**, Top view of the structure.

1. Supplementary Materials Figure S9: The 12-channel multifunctional module


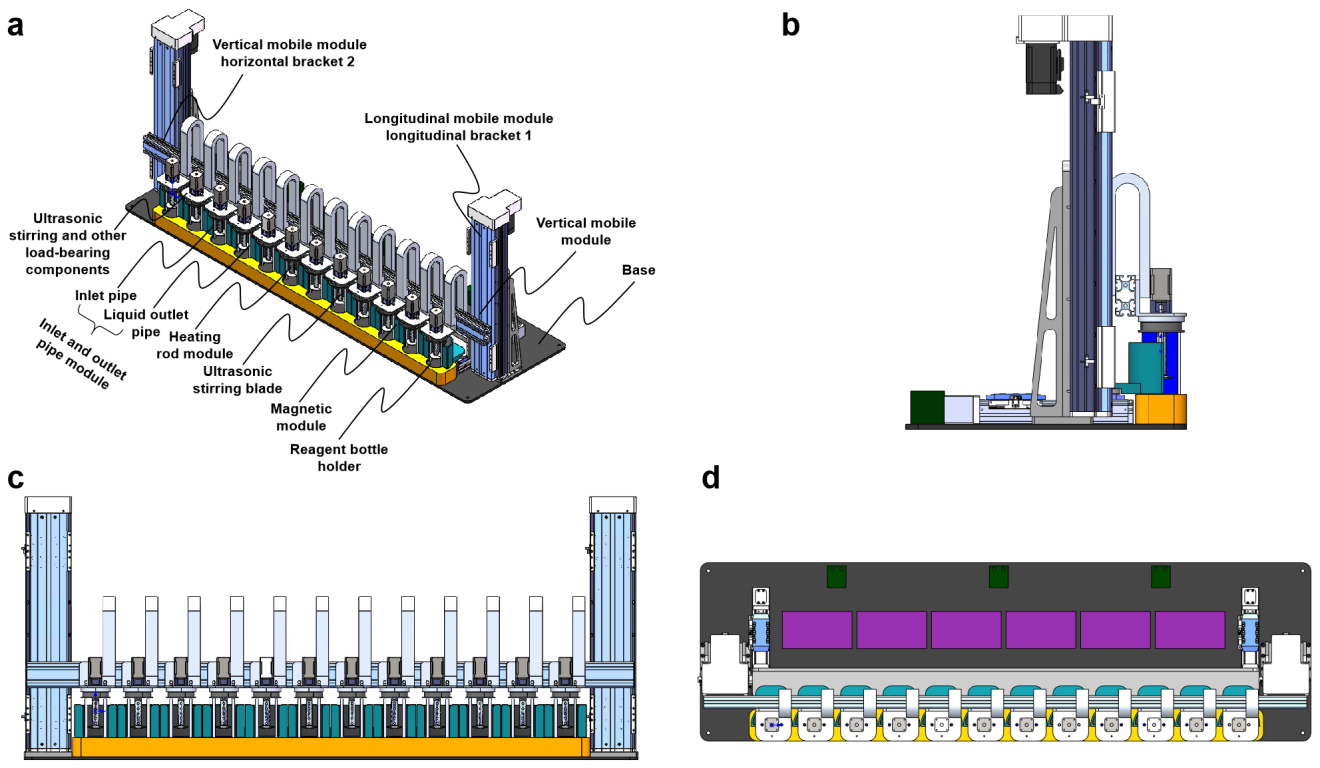


Figure S9. The structure diagram of the 12-channel multifunctional module. **a**, Schematic diagram of three-dimensional structure of the equipment. **b**, Side view of the structure. **c**, Front view of the structure. **d**, Top view of the structure.

1. Supplementary Materials Table S2: Detailed data of solid reagents feeding

Table S2. Detailed data of solid reagents feeding (average value of five measurements)

| Reagents | Properties | Target quantity | | | |
| --- | --- | --- | --- | --- | --- |
|  |  | 2 g | 5 g | 10 g | 20 g |
| Sodium bicarbonate | Powder | 1.982±0.024 | 4.995±0.018 | 10.13±0.016 | 19.974±0.029 |
| Guanidine isothiocyanate | Crystalline solid | 1.964±0.052 | 4.916±0.085 | 9.954±0.066 | 20.015±0.035 |
| PEG 2000 | Waxy solid | 1.907±0.095 | 4.937±0.082 | 9.915±0.094 | 19.916±0.092 |

11. Supplementary Materials Table S3: Detailed data of liquid reagents feeding

Table S3. Detailed data of liquid reagents feeding (average value of five measurements)

| Reagents | Target quantity | | | |
| --- | --- | --- | --- | --- |
|  | 2 g | 5 g | 10 g | 20 g |
| DI water | 2.000±0.004 | 4.996±0.008 | 10.001±0.007 | 20.002±0.011 |
| Triton | 1.982±0.026 | 5.024±0.036 | 10.054±0.062 | 19.966±0.065 |
| Isopropanol | 1.963±0.038 | 4.983±0.026 | 9.965±0.052 | 19.973±0.048 |

12. Supplementary Materials Figure S10: The Scanning Electron Microscope (SEM) images of magnetic cores and silica-coated MNPs


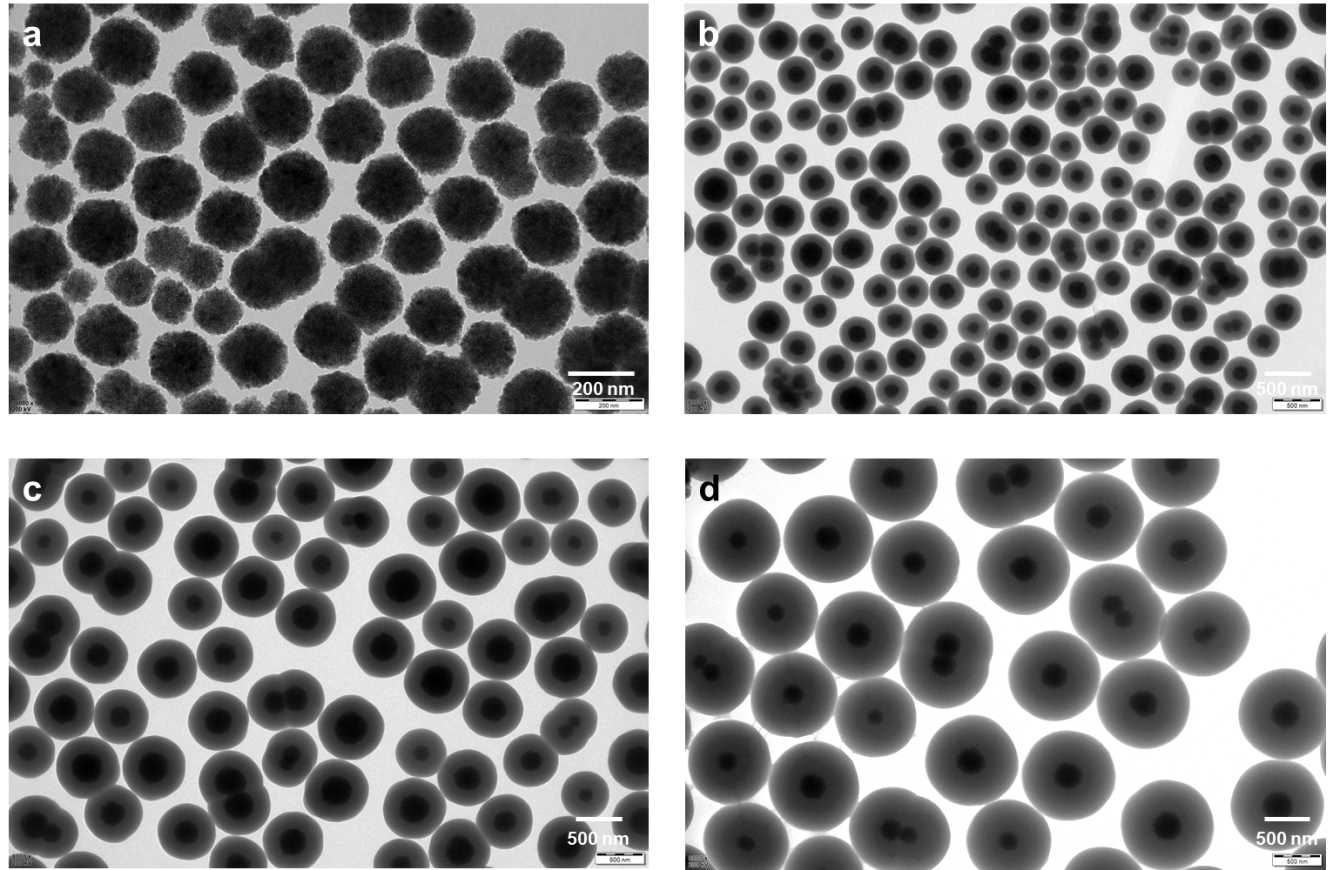


Figure S10. The SEM images of magnetic cores and silica-coated MNPs. **a**, The SEM image of 200 nm magnetic cores. **b**, The SEM image of 390 nm silica-coated MNPs. **c**, The SEM image of 600 nm silica-coated MNPs. **d**, The SEM image of 950 nm silica-coated MNPs.

13. Supplementary Materials Table S4: The Zeta potentials of silica-coated MNPs

Table S4. The Zeta potentials of silica-coated MNPs

| Magnetic core size  (before silica coating, nm) | Sample 1 (mv) | Sample 2 (mv) | Sample 3 (mv) |
| --- | --- | --- | --- |
| 100 | -17 | -18 | -17 |
| 200 | -20 | -22 | -21 |
| 300 | -40 | -41 | -42 |

14. Supplementary Materials Table S5: PCR batch-to-batch consistency testing results

Table S5. PCR batch-to-batch consistency testing results by testing SARS-CoV-2 pseudovirus samples with different concentrations

| Batch | Sample 1 | | Sample 2 | | Sample 3 | |
| --- | --- | --- | --- | --- | --- | --- |
|  | Ct | Avg Ct | Ct | Avg Ct | Ct | Avg Ct |
| 1 (10^3^ copies/mL SARS-CoV-2 pseudovirus) | 33.70 | 33.78±0.09 | 33.82 | 33.76±0.08 | 33.74 | 33.68±0.05 |
|  | 33.88 |  | 33.79 |  | 33.58 |  |
|  | 33.76 |  | 33.67 |  | 33.72 |  |
| 2 (10^4^ copies/mL SARS-CoV-2 pseudovirus) | 31.64 | 31.56±0.09 | 31.39 | 31.55±0.22 | 31.52 | 31.58±0.11 |
|  | 31.58 |  | 31.46 |  | 31.43 |  |
|  | 31.46 |  | 31.81 |  | 31.79 |  |
| 3 (10^5^ copies/mL SARS-CoV-2 pseudovirus) | 28.12 | 28.04±0.11 | 28.02 | 27.92±0.09 | 28.04 | 28.01±0.02 |
|  | 28.08 |  | 27.90 |  | 27.96 |  |
|  | 27.92 |  | 27.84 |  | 28.03 |  |

15. Supplementary Materials Table S6: PCR time-scale consistency testing results

Table S6. PCR time-scale consistency testing results by testing SARS-CoV-2 pseudovirus samples with a concentration of 10^5^ copies/mL and different extraction reagents systems

| agent ID | Lysis Buffer | MNPs | Initial Data (Ct) | Stability Test Day 1 | | Stability Test Day 2 | | Stability Test Day 3 | | Stability Test Day 4 | | Stability Test Day 5 | |
| --- | --- | --- | --- | --- | --- | --- | --- | --- | --- | --- | --- | --- | --- |
|  |  |  |  | Ct | Avg Ct | Ct | Avg Ct | Ct | Avg Ct | Ct | Avg Ct | Ct | Avg Ct |
| 1 | L1-11 | Multi-carboxyl-600nm | 35.15 | 34.91 | 34.94±0.03 | 35.11 | 35.10±0.02 | 35.17 | 35.19±0.03 | 35.14 | 35.06±0.11 | 35.08 | 35.03±0.07 |
|  |  |  |  | 34.95 |  | 35.11 |  | 35.22 |  | 34.95 |  | 34.96 |  |
|  |  |  |  | 34.96 |  | 35.08 |  | 35.18 |  | 35.08 |  | 35.05 |  |
| 2 | L4-1 | Single-carboxyl-1000nm | 30.06 | 32.18 | 32.07±0.11 | 32.12 | 32.08±0.04 | 31.94 | 31.94±0.02 | 32.08 | 32.06±0.02 | 31.93 | 31.93±0.03 |
|  |  |  |  | 31.98 |  | 32.02 |  | 31.95 |  | 32.06 |  | 31.96 |  |
|  |  |  |  | 32.05 |  | 32.10 |  | 31.92 |  | 32.04 |  | 31.90 |  |
| 3 | L1-1 | Single-carboxyl-1000nm | 28.07 | 28.03 | 28.11±0.21 | 28.24 | 28.08±0.16 | 28.15 | 28.04±0.19 | 28.20 | 28.09±0.14 | 28.08 | 28.01±0.07 |
|  |  |  |  | 28.32 |  | 28.01 |  | 28.12 |  | 28.12 |  | 27.97 |  |
|  |  |  |  | 27.98 |  | 27.99 |  | 27.85 |  | 27.95 |  | 27.98 |  |

16. Supplementary Materials Table S7: Five-batch experimental testing schemes and PCR testing results

Table S7. Five-batch experimental testing schemes and PCR testing results

| C2H6N4S (g) | Ammonium sulphate (g) | PEG 2000 (g) | Triton (g) | Isopropanol  (g) | DI water (wt%) | 0.5 M EDTA pH 8.0 (wt%) | 1 M Tris pH 8.0 (wt%) | Particle diameter (nm) | Carboxyl | Ct |
| --- | --- | --- | --- | --- | --- | --- | --- | --- | --- | --- |
| 15 | 0.75 | 5 | 7.5 | 15 | 0.4204 | 0.0420 | 0.0180 | 600 | 1 | 33.43 |
| 15 | 1.5 | 10 | 15 | 30 | 0.3139 | 0.0313 | 0.0134 | 600 | 1 | 32.24 |
| 15 | 3 | 20 | 30 | 60 | 0.2083 | 0.0208 | 0.0089 | 1000 | 3 | 34.21 |
| 15 | 3 | 20 | 30 | 60 | 0.2083 | 0.0208 | 0.0089 | 1000 | 3 | 37.35 |
| 60 | 3 | 5 | 15 | 30 | 0.2287 | 0.0228 | 0.0098 | 1000 | 3 | 35.21 |

C2H6N4S: Guanidine isothiocyanate; EDTA: Ethylenediaminetetraacetic acids; Carboxyl: 1 (Mono-carboxyl); 2 (Di-carboxylate); 3 (Poly-carboxyl);

1. Supplementary Materials Table S8: The comparison of PCR experimental results between the AP-Lab and HITL

Table S8. The comparison of PCR experimental results between the AP-Lab and HITL

| Scheme | AP-Lab | | | HITL | | |
| --- | --- | --- | --- | --- | --- | --- |
|  | Ct | Avg Ct | 1-σ | Ct | Avg Ct | 1-σ |
| 1 | 33.65 | 33.52±0.10 | 0.90 | 34.39 | 33.89±0.48 | 0.52 |
|  | 33.60 |  |  | 34.34 |  |  |
|  | 33.43 |  |  | 33.34 |  |  |
|  | 33.42 |  |  | 33.49 |  |  |
|  | 33.49 |  |  | 33.91 |  |  |
| 2 | 32.48 | 32.41±0.07 | 0.94 | 33.82 | 33.02±0.72 | 0.59 |
|  | 32.47 |  |  | 33.64 |  |  |
|  | 32.33 |  |  | 32.40 |  |  |
|  | 32.34 |  |  | 32.22 |  |  |
|  | 32.43 |  |  | 33.01 |  |  |
| 3 | 34.18 | 34.08±0.07 | 0.93 | 35.22 | 34.82±0.35 | 0.62 |
|  | 34.14 |  |  | 35.12 |  |  |
|  | 34.02 |  |  | 34.52 |  |  |
|  | 34.03 |  |  | 34.42 |  |  |
|  | 34.03 |  |  | 34.83 |  |  |
| 4 | 37.33 | 37.32±0.07 | 0.94 | 38.10 | 37.60±0.46 | 0.58 |
|  | 37.27 |  |  | 38.01 |  |  |
|  | 37.39 |  |  | 37.22 |  |  |
|  | 37.23 |  |  | 37.08 |  |  |
|  | 37.38 |  |  | 37.61 |  |  |
| 5 | 35.14 | 35.01±0.10 | 0.91 | 36.22 | 35.42±0.72 | 0.59 |
|  | 34.91 |  |  | 36.04 |  |  |
|  | 35.09 |  |  | 34.80 |  |  |
|  | 34.94 |  |  | 34.62 |  |  |
|  | 34.97 |  |  | 35.41 |  |  |

1. Supplementary Materials Table S9 and S10: Representative industrial datasets

Table S9. Representative initial datasets from industry (I)

| *chaotropic reagent* | *salting out reagent* | *molecular crowding reagent* | *surfactant* | *precipitation reagent* | *organic solvent* | *chelation reagent* | *buffer* | *particle size* | *functional group* |  |
| --- | --- | --- | --- | --- | --- | --- | --- | --- | --- | --- |
| C2H6N4S (g) | Ammonium sulphate (g) | PEG 2000 (g) | Triton (g) | Isopropanol  (g) | DI water (wt%) | 0.5 M EDTA pH 8.0 (wt%) | 1 M Tris pH 8.0 (wt%) | Particle diameter (nm) | Carboxyl | Ct |
| 15 | 0.75 | 5 | 7.5 | 15 | 0.4204 | 0.0420 | 0.0180 | 600 | 1 | 33.43 |
| 15 | 0.75 | 5 | 7.5 | 15 | 0.4204 | 0.0420 | 0.0180 | 600 | 1 | 32.76 |
| 15 | 0.75 | 5 | 7.5 | 15 | 0.4204 | 0.0420 | 0.0180 | 600 | 1 | 32.82 |
| 15 | 0.75 | 5 | 7.5 | 15 | 0.4204 | 0.0420 | 0.0180 | 600 | 3 | 33.27 |
| 15 | 0.75 | 5 | 7.5 | 15 | 0.4204 | 0.0420 | 0.0180 | 600 | 3 | 32.30 |
| 15 | 0.75 | 5 | 7.5 | 15 | 0.4204 | 0.0420 | 0.0180 | 600 | 3 | 32.08 |
| 15 | 0.75 | 5 | 7.5 | 15 | 0.4204 | 0.0420 | 0.0180 | 1000 | 3 | 33.16 |
| 15 | 0.75 | 5 | 7.5 | 15 | 0.4204 | 0.0420 | 0.0180 | 1000 | 3 | 32.45 |
| 15 | 0.75 | 5 | 7.5 | 15 | 0.4204 | 0.0420 | 0.0180 | 1000 | 3 | 33.26 |
| 15 | 1.5 | 10 | 15 | 30 | 0.3139 | 0.0313 | 0.0134 | 600 | 1 | 32.24 |
| 15 | 1.5 | 10 | 15 | 30 | 0.3139 | 0.0313 | 0.0134 | 600 | 1 | 31.86 |
| 15 | 1.5 | 10 | 15 | 30 | 0.3139 | 0.0313 | 0.0134 | 600 | 1 | 31.00 |
| 15 | 1.5 | 10 | 15 | 30 | 0.3139 | 0.0313 | 0.0134 | 600 | 3 | 32.01 |
| 15 | 1.5 | 10 | 15 | 30 | 0.3139 | 0.0313 | 0.0134 | 600 | 3 | 31.44 |
| 15 | 1.5 | 10 | 15 | 30 | 0.3139 | 0.0313 | 0.0134 | 600 | 3 | 31.14 |
| 15 | 1.5 | 10 | 15 | 30 | 0.3139 | 0.0313 | 0.0134 | 1000 | 3 | 33.11 |
| 15 | 1.5 | 10 | 15 | 30 | 0.3139 | 0.0313 | 0.0134 | 1000 | 3 | 32.39 |
| 15 | 1.5 | 10 | 15 | 30 | 0.3139 | 0.0313 | 0.0134 | 1000 | 3 | 33.11 |
| 15 | 3 | 20 | 30 | 60 | 0.2083 | 0.0208 | 0.0089 | 600 | 1 | 31.82 |
| 15 | 3 | 20 | 30 | 60 | 0.2083 | 0.0208 | 0.0089 | 600 | 1 | 31.63 |
| 15 | 3 | 20 | 30 | 60 | 0.2083 | 0.0208 | 0.0089 | 600 | 1 | 31.54 |
| 15 | 3 | 20 | 30 | 60 | 0.2083 | 0.0208 | 0.0089 | 600 | 3 | 34.51 |
| 15 | 3 | 20 | 30 | 60 | 0.2083 | 0.0208 | 0.0089 | 600 | 3 | 32.54 |
| 15 | 3 | 20 | 30 | 60 | 0.2083 | 0.0208 | 0.0089 | 600 | 3 | 32.40 |
| 15 | 3 | 20 | 30 | 60 | 0.2083 | 0.0208 | 0.0089 | 1000 | 3 | 37.35 |
| 15 | 3 | 20 | 30 | 60 | 0.2083 | 0.0208 | 0.0089 | 1000 | 3 | 33.08 |
| 15 | 3 | 20 | 30 | 60 | 0.2083 | 0.0208 | 0.0089 | 1000 | 3 | 34.21 |
| 15 | 1.5 | 10 | 15 | 30 | 0.3139 | 0.0313 | 0.0134 | 600 | 1 | 31.91 |
| 15 | 1.5 | 10 | 15 | 30 | 0.3139 | 0.0313 | 0.0134 | 600 | 1 | 31.84 |
| 15 | 1.5 | 10 | 15 | 30 | 0.3139 | 0.0313 | 0.0134 | 600 | 1 | 32.07 |
| 15 | 1.5 | 10 | 15 | 30 | 0.3139 | 0.0313 | 0.0134 | 600 | 3 | 30.79 |
| 15 | 1.5 | 10 | 15 | 30 | 0.3139 | 0.0313 | 0.0134 | 600 | 3 | 33.08 |
| 15 | 1.5 | 10 | 15 | 30 | 0.3139 | 0.0313 | 0.0134 | 600 | 3 | 31.91 |
| 15 | 1.5 | 10 | 15 | 30 | 0.3139 | 0.0313 | 0.0134 | 1000 | 3 | 34.35 |
| 15 | 1.5 | 10 | 15 | 30 | 0.3139 | 0.0313 | 0.0134 | 1000 | 3 | 32.59 |
| 15 | 1.5 | 10 | 15 | 30 | 0.3139 | 0.0313 | 0.0134 | 1000 | 3 | 32.27 |
| 30 | 0.75 | 10 | 30 | 30 | 0.2486 | 0.0248 | 0.0106 | 600 | 1 | 32.41 |
| 30 | 0.75 | 10 | 30 | 30 | 0.2486 | 0.0248 | 0.0106 | 600 | 1 | 31.50 |
| 30 | 0.75 | 10 | 30 | 30 | 0.2486 | 0.0248 | 0.0106 | 600 | 1 | 31.37 |
| 30 | 0.75 | 10 | 30 | 30 | 0.2486 | 0.0248 | 0.0106 | 600 | 3 | 32.44 |
| 30 | 0.75 | 10 | 30 | 30 | 0.2486 | 0.0248 | 0.0106 | 600 | 3 | 31.91 |
| 30 | 0.75 | 10 | 30 | 30 | 0.2486 | 0.0248 | 0.0106 | 600 | 3 | 32.20 |
| 30 | 0.75 | 10 | 30 | 30 | 0.2486 | 0.0248 | 0.0106 | 1000 | 3 | 31.95 |
| 30 | 0.75 | 10 | 30 | 30 | 0.2486 | 0.0248 | 0.0106 | 1000 | 3 | 32.69 |
| 30 | 0.75 | 10 | 30 | 30 | 0.2486 | 0.0248 | 0.0106 | 1000 | 3 | 32.30 |
| 30 | 1.5 | 5 | 15 | 60 | 0.2310 | 0.0231 | 0.0099 | 600 | 1 | 31.33 |
| 30 | 1.5 | 5 | 15 | 60 | 0.2310 | 0.0231 | 0.0099 | 600 | 1 | 31.40 |
| 30 | 1.5 | 5 | 15 | 60 | 0.2310 | 0.0231 | 0.0099 | 600 | 1 | 31.80 |
| 30 | 1.5 | 5 | 15 | 60 | 0.2310 | 0.0231 | 0.0099 | 600 | 3 | 32.60 |
| 30 | 1.5 | 5 | 15 | 60 | 0.2310 | 0.0231 | 0.0099 | 600 | 3 | 31.12 |
| 30 | 1.5 | 5 | 15 | 60 | 0.2310 | 0.0231 | 0.0099 | 600 | 3 | 31.87 |
| 30 | 1.5 | 5 | 15 | 60 | 0.2310 | 0.0231 | 0.0099 | 1000 | 3 | 33.11 |
| 30 | 1.5 | 5 | 15 | 60 | 0.2310 | 0.0231 | 0.0099 | 1000 | 3 | 34.06 |
| 30 | 1.5 | 5 | 15 | 60 | 0.2310 | 0.0231 | 0.0099 | 1000 | 3 | 34.14 |
| 30 | 3 | 10 | 7.5 | 30 | 0.2904 | 0.0290 | 0.0124 | 600 | 1 | 32.08 |
| 30 | 3 | 10 | 7.5 | 30 | 0.2904 | 0.0290 | 0.0124 | 600 | 1 | 32.48 |
| 30 | 3 | 10 | 7.5 | 30 | 0.2904 | 0.0290 | 0.0124 | 600 | 1 | 31.36 |
| 30 | 3 | 10 | 7.5 | 30 | 0.2904 | 0.0290 | 0.0124 | 600 | 3 | 32.34 |
| 30 | 3 | 10 | 7.5 | 30 | 0.2904 | 0.0290 | 0.0124 | 600 | 3 | 32.94 |
| 30 | 3 | 10 | 7.5 | 30 | 0.2904 | 0.0290 | 0.0124 | 600 | 3 | 31.95 |
| 30 | 3 | 10 | 7.5 | 30 | 0.2904 | 0.0290 | 0.0124 | 1000 | 3 | 31.62 |
| 30 | 3 | 10 | 7.5 | 30 | 0.2904 | 0.0290 | 0.0124 | 1000 | 3 | 32.42 |
| 30 | 3 | 10 | 7.5 | 30 | 0.2904 | 0.0290 | 0.0124 | 1000 | 3 | 31.66 |
| 30 | 1.5 | 20 | 15 | 15 | 0.2880 | 0.0288 | 0.0123 | 600 | 1 | 32.13 |
| 30 | 1.5 | 20 | 15 | 15 | 0.2880 | 0.0288 | 0.0123 | 600 | 1 | 32.37 |
| 30 | 1.5 | 20 | 15 | 15 | 0.2880 | 0.0288 | 0.0123 | 600 | 1 | 32.17 |
| 30 | 1.5 | 20 | 15 | 15 | 0.2880 | 0.0288 | 0.0123 | 600 | 3 | 33.83 |
| 30 | 1.5 | 20 | 15 | 15 | 0.2880 | 0.0288 | 0.0123 | 600 | 3 | 34.54 |
| 30 | 1.5 | 20 | 15 | 15 | 0.2880 | 0.0288 | 0.0123 | 600 | 3 | 32.56 |
| 30 | 1.5 | 20 | 15 | 15 | 0.2880 | 0.0288 | 0.0123 | 1000 | 3 | 33.16 |
| 30 | 1.5 | 20 | 15 | 15 | 0.2880 | 0.0288 | 0.0123 | 1000 | 3 | 34.17 |
| 30 | 1.5 | 20 | 15 | 15 | 0.2880 | 0.0288 | 0.0123 | 1000 | 3 | 33.56 |
| 60 | 0.75 | 20 | 15 | 30 | 0.2111 | 0.0211 | 0.0090 | 600 | 1 | 33.18 |
| 60 | 0.75 | 20 | 15 | 30 | 0.2111 | 0.0211 | 0.0090 | 600 | 1 | 32.14 |
| 60 | 0.75 | 20 | 15 | 30 | 0.2111 | 0.0211 | 0.0090 | 600 | 1 | 31.56 |
| 60 | 0.75 | 20 | 15 | 30 | 0.2111 | 0.0211 | 0.0090 | 600 | 3 | 32.08 |
| 60 | 0.75 | 20 | 15 | 30 | 0.2111 | 0.0211 | 0.0090 | 600 | 3 | 32.35 |
| 60 | 0.75 | 20 | 15 | 30 | 0.2111 | 0.0211 | 0.0090 | 600 | 3 | 31.54 |
| 60 | 0.75 | 20 | 15 | 30 | 0.2111 | 0.0211 | 0.0090 | 1000 | 3 | 32.02 |
| 60 | 0.75 | 20 | 15 | 30 | 0.2111 | 0.0211 | 0.0090 | 1000 | 3 | 32.04 |
| 60 | 0.75 | 20 | 15 | 30 | 0.2111 | 0.0211 | 0.0090 | 1000 | 3 | 32.65 |
| 60 | 1.5 | 10 | 30 | 15 | 0.2236 | 0.0223 | 0.0095 | 600 | 1 | 31.59 |
| 60 | 1.5 | 10 | 30 | 15 | 0.2236 | 0.0223 | 0.0095 | 600 | 1 | 31.35 |
| 60 | 1.5 | 10 | 30 | 15 | 0.2236 | 0.0223 | 0.0095 | 600 | 1 | 31.55 |
| 60 | 1.5 | 10 | 30 | 15 | 0.2236 | 0.0223 | 0.0095 | 600 | 3 | 32.82 |
| 60 | 1.5 | 10 | 30 | 15 | 0.2236 | 0.0223 | 0.0095 | 600 | 3 | 31.89 |
| 60 | 1.5 | 10 | 30 | 15 | 0.2236 | 0.0223 | 0.0095 | 600 | 3 | 33.61 |
| 60 | 1.5 | 10 | 30 | 15 | 0.2236 | 0.0223 | 0.0095 | 1000 | 3 | 32.85 |
| 60 | 1.5 | 10 | 30 | 15 | 0.2236 | 0.0223 | 0.0095 | 1000 | 3 | 31.71 |
| 60 | 1.5 | 10 | 30 | 15 | 0.2236 | 0.0223 | 0.0095 | 1000 | 3 | 32.85 |
| 60 | 3 | 5 | 15 | 30 | 0.228 | 0.0228 | 0.0098 | 600 | 1 | 33.44 |
| 60 | 3 | 5 | 15 | 30 | 0.228 | 0.0228 | 0.0098 | 600 | 1 | 33.98 |
| 60 | 3 | 5 | 15 | 30 | 0.228 | 0.0228 | 0.0098 | 600 | 1 | 33.59 |
| 60 | 3 | 5 | 15 | 30 | 0.228 | 0.0228 | 0.0098 | 600 | 3 | 34.92 |
| 60 | 3 | 5 | 15 | 30 | 0.228 | 0.0228 | 0.0098 | 600 | 3 | 34.81 |
| 60 | 3 | 5 | 15 | 30 | 0.228 | 0.0228 | 0.0098 | 600 | 3 | 35.72 |
| 60 | 3 | 5 | 15 | 30 | 0.228 | 0.0228 | 0.0098 | 1000 | 1 | 30.59 |
| 60 | 3 | 5 | 15 | 30 | 0.228 | 0.0228 | 0.0098 | 1000 | 1 | 30.29 |
| 60 | 3 | 5 | 15 | 30 | 0.228 | 0.0228 | 0.0098 | 1000 | 1 | 30.11 |
| 60 | 3 | 5 | 15 | 30 | 0.2287 | 0.0228 | 0.0098 | 1000 | 3 | 35.21 |
| 60 | 3 | 5 | 15 | 30 | 0.2287 | 0.0228 | 0.0098 | 1000 | 3 | 34.37 |
| 60 | 3 | 5 | 15 | 30 | 0.2287 | 0.0228 | 0.0098 | 1000 | 3 | 35.36 |
| 60 | 1.5 | 10 | 7.5 | 60 | 0.1955 | 0.0228 | 0.0098 | 600 | 1 | 31.41 |
| 60 | 1.5 | 10 | 7.5 | 60 | 0.1955 | 0.0195 | 0.0083 | 600 | 1 | 31.27 |
| 60 | 1.5 | 10 | 7.5 | 60 | 0.1955 | 0.0195 | 0.0083 | 600 | 1 | 31.8 |
| 60 | 1.5 | 10 | 7.5 | 60 | 0.1955 | 0.0195 | 0.0083 | 600 | 3 | 33.76 |
| 60 | 1.5 | 10 | 7.5 | 60 | 0.1955 | 0.0195 | 0.0083 | 600 | 3 | 32.33 |
| 60 | 1.5 | 10 | 7.5 | 60 | 0.1955 | 0.0195 | 0.0083 | 600 | 3 | 32.65 |
| 60 | 1.5 | 10 | 7.5 | 60 | 0.1955 | 0.0195 | 0.0083 | 1000 | 3 | 32.45 |
| 60 | 1.5 | 10 | 7.5 | 60 | 0.1955 | 0.0195 | 0.0083 | 1000 | 3 | 32.34 |
| 60 | 1.5 | 10 | 7.5 | 60 | 0.1955 | 0.0195 | 0.0083 | 1000 | 3 | 32.09 |

Table S10. Representative initial datasets from industry (II)

| *chaotropic reagent* | *salting out reagent* | *molecular crowding reagent* | *surfactant* | *precipitation reagent* | *organic solvent* | *chelation reagent* | *buffer* | *particle size* | *functional group* |  |
| --- | --- | --- | --- | --- | --- | --- | --- | --- | --- | --- |
| Guanidine hydrochloride (g) | NaCl (g) | PEG 6000 (g) | SDS (g) | Ethanol  (g) | DI water (wt%) | 0.5 M EDTA pH 8.0 (wt%) | 1 M Tris pH 8.0 (wt%) | Particle diameter (nm) | Carboxyl | Ct |
| 30 | 0.75 | 10 | 15 | 60 | 0.2247 | 0.0224 | 0.0096 | 600 | 1 | 31.39 |
| 30 | 0.75 | 10 | 15 | 60 | 0.2247 | 0.0224 | 0.0096 | 600 | 1 | 31.39 |
| 30 | 0.75 | 10 | 15 | 60 | 0.2247 | 0.0224 | 0.0096 | 600 | 1 | 31.23 |
| 30 | 0.75 | 10 | 15 | 60 | 0.2247 | 0.0224 | 0.0096 | 600 | 3 | 31.33 |
| 30 | 0.75 | 10 | 15 | 60 | 0.2247 | 0.0224 | 0.0096 | 600 | 3 | 31.38 |
| 30 | 0.75 | 10 | 15 | 60 | 0.2247 | 0.0224 | 0.0096 | 600 | 3 | 32.24 |
| 30 | 0.75 | 10 | 15 | 60 | 0.2247 | 0.0224 | 0.0096 | 1000 | 3 | 32.53 |
| 30 | 0.75 | 10 | 15 | 60 | 0.2247 | 0.0224 | 0.0096 | 1000 | 3 | 31.29 |
| 30 | 0.75 | 10 | 15 | 60 | 0.2247 | 0.0224 | 0.0096 | 1000 | 3 | 31.15 |
| 30 | 1.5 | 20 | 7.5 | 30 | 0.2713 | 0.0271 | 0.0116 | 600 | 1 | 31.17 |
| 30 | 1.5 | 20 | 7.5 | 30 | 0.2713 | 0.0271 | 0.0116 | 600 | 1 | 31.75 |
| 30 | 1.5 | 20 | 7.5 | 30 | 0.2713 | 0.0271 | 0.0116 | 600 | 1 | 31.28 |
| 30 | 1.5 | 20 | 7.5 | 30 | 0.2713 | 0.0271 | 0.0116 | 600 | 3 | 33.47 |
| 30 | 1.5 | 20 | 7.5 | 30 | 0.2713 | 0.0271 | 0.0116 | 600 | 3 | 32.4 |
| 30 | 1.5 | 20 | 7.5 | 30 | 0.2713 | 0.0271 | 0.0116 | 600 | 3 | 33.68 |
| 30 | 1.5 | 20 | 7.5 | 30 | 0.2713 | 0.0271 | 0.0116 | 1000 | 3 | 33.36 |
| 30 | 1.5 | 20 | 7.5 | 30 | 0.2713 | 0.0271 | 0.0116 | 1000 | 3 | 32.25 |
| 30 | 1.5 | 20 | 7.5 | 30 | 0.2713 | 0.0271 | 0.0116 | 1000 | 3 | 32.99 |
| 30 | 3 | 10 | 15 | 15 | 0.3097 | 0.0309 | 0.0132 | 600 | 1 | 32.84 |
| 30 | 3 | 10 | 15 | 15 | 0.3097 | 0.0309 | 0.0132 | 600 | 1 | 32.65 |
| 30 | 3 | 10 | 15 | 15 | 0.3097 | 0.0309 | 0.0132 | 600 | 1 | 31.69 |
| 30 | 3 | 10 | 15 | 15 | 0.3097 | 0.0309 | 0.0132 | 600 | 3 | 33.13 |
| 30 | 3 | 10 | 15 | 15 | 0.3097 | 0.0309 | 0.0132 | 600 | 3 | 33.62 |
| 30 | 3 | 10 | 15 | 15 | 0.3097 | 0.0309 | 0.0132 | 600 | 3 | 31.99 |
| 30 | 3 | 10 | 15 | 15 | 0.3097 | 0.0309 | 0.0132 | 1000 | 3 | 33.51 |
| 30 | 3 | 10 | 15 | 15 | 0.3097 | 0.0309 | 0.0132 | 1000 | 3 | 32.38 |
| 30 | 3 | 10 | 15 | 15 | 0.3097 | 0.0309 | 0.0132 | 1000 | 3 | 31.92 |
| 30 | 1.5 | 5 | 30 | 30 | 0.2564 | 0.0256 | 0.0109 | 600 | 1 | 32.02 |
| 30 | 1.5 | 5 | 30 | 30 | 0.2564 | 0.0256 | 0.0109 | 600 | 1 | 32.29 |
| 30 | 1.5 | 5 | 30 | 30 | 0.2564 | 0.0256 | 0.0109 | 600 | 1 | 31.58 |
| 30 | 1.5 | 5 | 30 | 30 | 0.2564 | 0.0256 | 0.0109 | 600 | 3 | 33.12 |
| 30 | 1.5 | 5 | 30 | 30 | 0.2564 | 0.0256 | 0.0109 | 600 | 3 | 33.78 |
| 30 | 1.5 | 5 | 30 | 30 | 0.2564 | 0.0256 | 0.0109 | 600 | 3 | 33.43 |
| 30 | 1.5 | 5 | 30 | 30 | 0.2564 | 0.0256 | 0.0109 | 1000 | 3 | 31.54 |
| 30 | 1.5 | 5 | 30 | 30 | 0.2564 | 0.0256 | 0.0109 | 1000 | 3 | 30.96 |
| 30 | 1.5 | 5 | 30 | 30 | 0.2564 | 0.0256 | 0.0109 | 1000 | 3 | 31.37 |
| Carboxyl: 1 (Mono-carboxyl); 2 (Di-carboxylate); 3 (Poly-carboxyl) | | | | | | | | | | |

1. Supplementary Materials Figure S11: The relationship between virus concentrations and Ct values


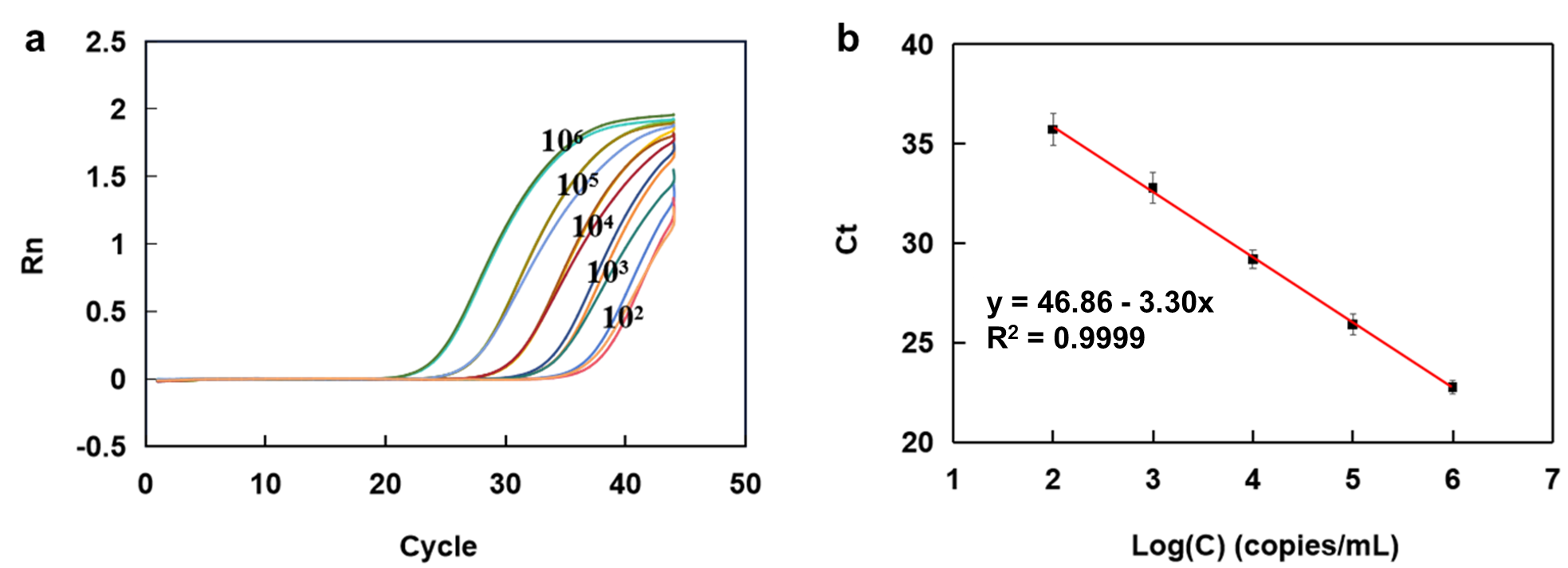


Figure S11. At the concentration of 10^5^ copies/mL, achieving a Ct value of approximately 26 corresponds to an extraction efficiency exceeding 99%. **a**, The PCR curves of different NAs concentrations. **b**, The LOD (Limit of Detection) of NAs extraction.

1. Supplementary Materials M1: Detailed optimization schemes and results of each iteration for SARS-CoV-2 detection

22.1. First-round recommended schemes and autonomous PCR testing results

Table S11. Optimization schemes and PCR testing results from the first iteration (I)

| No. | C_2_H_6_N_4_S (g) | Ammonium sulphate (g) | PEG 2000 (g) | Triton (g) | Isopropanol  (g) | DI water (wt%) | 0.5 M EDTA pH 8.0 (wt%) | 1 M Tris pH 8.0 (wt%) | Particle diameter (nm) | Carboxyl | Predicted Ct | Tested Ct |
| --- | --- | --- | --- | --- | --- | --- | --- | --- | --- | --- | --- | --- |
| 1 | 215.118 | 9.948 | 23.031 | 93.592 | 97.488 | 0.1955 | 0.0266 | 0.0093 | 600 | 2 | 34.320 | 35.98 |
| 2 | 182.455 | 7.054 | 20.796 | 114.798 | 161.392 | 0.2822 | 0.0199 | 0.0106 | 700 | 2 | 33.161 | 34.58 |
| 3 | 203.378 | 4.651 | 66.782 | 83.716 | 140.252 | 0.2186 | 0.0299 | 0.0116 | 700 | 2 | 33.134 | 35.15 |
| 4 | 70.154 | 9.498 | 76.313 | 30.075 | 238.598 | 0.3344 | 0.0333 | 0.0084 | 600 | 2 | 33.053 | 35.03 |
| 5 | 131.975 | 3.425 | 78.425 | 50.959 | 76.309 | 0.3346 | 0.0282 | 0.0179 | 800 | 2 | 32.973 | 36.25 |
| 6 | 211.611 | 7.048 | 43.709 | 113.399 | 190.909 | 0.2695 | 0.0324 | 0.0134 | 1000 | 2 | 32.934 | 36.06 |
| 7 | 131.079 | 5.641 | 20.845 | 47.896 | 188.042 | 0.3732 | 0.0332 | 0.0173 | 900 | 2 | 32.877 | 36.55 |
| 8 | 240.007 | 12.009 | 80.006 | 120.003 | 60.006 | 0.3711 | 0.0425 | 0.018 | 700 | 1 | 32.672 | 34.72 |
| 9 | 195.458 | 3.009 | 20.004 | 120.002 | 78.773 | 0.3232 | 0.0208 | 0.0143 | 600 | 1 | 32.513 | 35.48 |
| 10 | 234.908 | 10.645 | 63.304 | 51.239 | 106.092 | 0.2046 | 0.0355 | 0.0094 | 800 | 1 | 32.465 | 34.59 |
| 11 | 240.008 | 3.688 | 20.007 | 120.003 | 169.415 | 0.3337 | 0.0196 | 0.0156 | 600 | 1 | 32.418 | 35.02 |
| 12 | 239.208 | 11.777 | 56.443 | 31.962 | 69.086 | 0.1955 | 0.0342 | 0.0119 | 700 | 1 | 32.408 | 35.67 |
| 13 | 64.384 | 8.756 | 46.344 | 120.007 | 88.563 | 0.4204 | 0.0426 | 0.0169 | 700 | 1 | 32.356 | 34.58 |
| 14 | 233.767 | 8.453 | 20.142 | 120.003 | 229.513 | 0.4081 | 0.0416 | 0.0113 | 600 | 1 | 32.306 | 34.97 |
| 15 | 240.005 | 4.738 | 20.002 | 120.001 | 240.003 | 0.4204 | 0.042 | 0.0156 | 700 | 1 | 32.298 | 36.56 |
| 16 | 196.032 | 11.616 | 74.177 | 120.002 | 195.796 | 0.2454 | 0.0396 | 0.0186 | 700 | 1 | 32.265 | 35.76 |
| 17 | 234.038 | 3.007 | 78.272 | 120.007 | 122.099 | 0.3951 | 0.0425 | 0.0102 | 700 | 1 | 32.209 | 35.02 |
| 18 | 137.768 | 10.812 | 80.001 | 30.007 | 145.322 | 0.4204 | 0.0426 | 0.0187 | 700 | 1 | 32.145 | 35.86 |
| 19 | 240.004 | 3.003 | 20.005 | 120.008 | 240.006 | 0.1955 | 0.0196 | 0.0176 | 600 | 1 | 32.107 | 36.37 |
| 20 | 240.005 | 3.005 | 20.005 | 120.009 | 240.004 | 0.1955 | 0.0196 | 0.0186 | 600 | 1 | 32.107 | 34.53 |
| 21 | 133.227 | 11.908 | 80.007 | 30.008 | 117.232 | 0.2337 | 0.0411 | 0.0084 | 600 | 1 | 32.104 | 36.36 |
| 22 | 60.005 | 9.064 | 50.314 | 111.947 | 106.104 | 0.2516 | 0.0231 | 0.0107 | 600 | 1 | 32.095 | 36.11 |
| 23 | 200.747 | 8.794 | 20.003 | 40.819 | 199.674 | 0.2347 | 0.0258 | 0.0156 | 600 | 1 | 32.077 | 35.34 |
| 24 | 60.007 | 11.213 | 77.911 | 30.007 | 124.978 | 0.1955 | 0.0427 | 0.0187 | 700 | 1 | 32.071 | 34.87 |

Table S12. Optimization schemes and PCR testing results from the first iteration (II)

| No. | Guanidine isothiocyanate (g) | Potassium chloride (g) | PEG 2000 (g) | SDS (g) | Ethanol (g) | Phenol (wt%) | EGTA (wt%) | MOPS (wt%) | Particle diameter (nm) | Carboxyl | Predicted Ct | Tested Ct |
| --- | --- | --- | --- | --- | --- | --- | --- | --- | --- | --- | --- | --- |
| 25 | 78.798 | 9.497 | 80.005 | 49.292 | 183.601 | 0.3956 | 0.0425 | 0.0184 | 700 | 1 | 32.065 | 36.74 |
| 26 | 208.025 | 7.637 | 77.561 | 81.402 | 240.009 | 0.2916 | 0.0424 | 0.0159 | 700 | 1 | 32.059 | 35.29 |
| 27 | 60.001 | 3.267 | 80.002 | 30.001 | 169.712 | 0.4204 | 0.0426 | 0.0185 | 600 | 1 | 32.043 | 34.73 |
| 28 | 240.002 | 8.304 | 80.003 | 80.034 | 186.252 | 0.4204 | 0.0269 | 0.0185 | 700 | 1 | 32.033 | 34.74 |
| 29 | 240.008 | 7.528 | 52.043 | 35.641 | 240.005 | 0.4204 | 0.0426 | 0.0171 | 600 | 1 | 32.021 | 36.45 |
| 30 | 206.442 | 3.672 | 78.948 | 120.009 | 229.604 | 0.3748 | 0.0253 | 0.0185 | 700 | 1 | 32.013 | 35.94 |
| 31 | 188.432 | 8.186 | 62.934 | 116.757 | 173.209 | 0.2524 | 0.0271 | 0.0099 | 600 | 1 | 31.991 | 36.36 |
| 32 | 117.977 | 10.745 | 80.004 | 100.606 | 240.002 | 0.1955 | 0.0425 | 0.0148 | 700 | 1 | 31.975 | 36.18 |
| 33 | 240.008 | 4.182 | 70.794 | 120.005 | 196.436 | 0.3056 | 0.0426 | 0.0147 | 700 | 1 | 31.946 | 35.74 |
| 34 | 96.359 | 11.108 | 80.004 | 82.106 | 195.304 | 0.1955 | 0.0425 | 0.0153 | 700 | 1 | 31.939 | 36.74 |
| 35 | 240.002 | 8.757 | 66.904 | 120.003 | 157.921 | 0.2368 | 0.0224 | 0.0085 | 600 | 1 | 31.932 | 35.38 |
| 36 | 102.831 | 5.938 | 42.793 | 74.334 | 221.166 | 0.3796 | 0.0424 | 0.0185 | 600 | 1 | 31.934 | 35.78 |

Table S13. Optimization schemes and PCR testing results from the first iteration (III)

| No. | Guanidine isothiocyanate (g) | Potassium chloride (g) | PEG 8000 (g) | Triton X-100 (g) | Ethanol (g) | Methanol (wt%) | DTPA (wt%) | Tris-HCl (wt%) | Particle diameter (nm) | Carboxyl | Predicted Ct | Tested Ct |
| --- | --- | --- | --- | --- | --- | --- | --- | --- | --- | --- | --- | --- |
| 37 | 194.518 | 7.857 | 55.205 | 116.873 | 169.266 | 0.2576 | 0.0262 | 0.0145 | 600 | 1 | 31.891 | 36.41 |
| 38 | 124.286 | 7.044 | 71.129 | 89.204 | 176.479 | 0.2627 | 0.0283 | 0.0136 | 600 | 1 | 31.886 | 35.93 |
| 39 | 240.002 | 3.008 | 45.508 | 37.255 | 199.252 | 0.3108 | 0.0425 | 0.0167 | 600 | 1 | 31.877 | 36.48 |
| 40 | 60.009 | 12.007 | 80.003 | 120.001 | 240.006 | 0.1955 | 0.0285 | 0.0084 | 600 | 1 | 31.874 | 35.84 |
| 41 | 165.198 | 8.406 | 69.448 | 120.007 | 179.538 | 0.2482 | 0.0291 | 0.0104 | 600 | 1 | 31.844 | 36.13 |
| 42 | 87.757 | 3.501 | 21.388 | 34.549 | 225.781 | 0.3346 | 0.0292 | 0.0095 | 700 | 1 | 31.773 | 34.62 |
| 43 | 174.328 | 8.822 | 49.428 | 69.743 | 221.032 | 0.2491 | 0.0417 | 0.0155 | 600 | 1 | 31.735 | 35.04 |
| 44 | 137.678 | 5.197 | 67.096 | 120.002 | 240.006 | 0.2288 | 0.0271 | 0.0146 | 700 | 1 | 31.718 | 35.18 |
| 45 | 60.009 | 3.406 | 78.829 | 62.532 | 240.004 | 0.1955 | 0.0403 | 0.0139 | 700 | 1 | 31.694 | 34.77 |
| 46 | 60.006 | 5.239 | 46.544 | 30.003 | 240.003 | 0.1955 | 0.0374 | 0.0087 | 700 | 1 | 31.655 | 35.05 |
| 47 | 60.003 | 7.374 | 24.148 | 57.206 | 195.452 | 0.4204 | 0.0246 | 0.0186 | 1000 | 1 | 29.527 | 34.75 |
| 48 | 213.007 | 7.045 | 25.725 | 63.374 | 180.392 | 0.3453 | 0.0329 | 0.0115 | 800 | 1 | 29.327 | 35.15 |


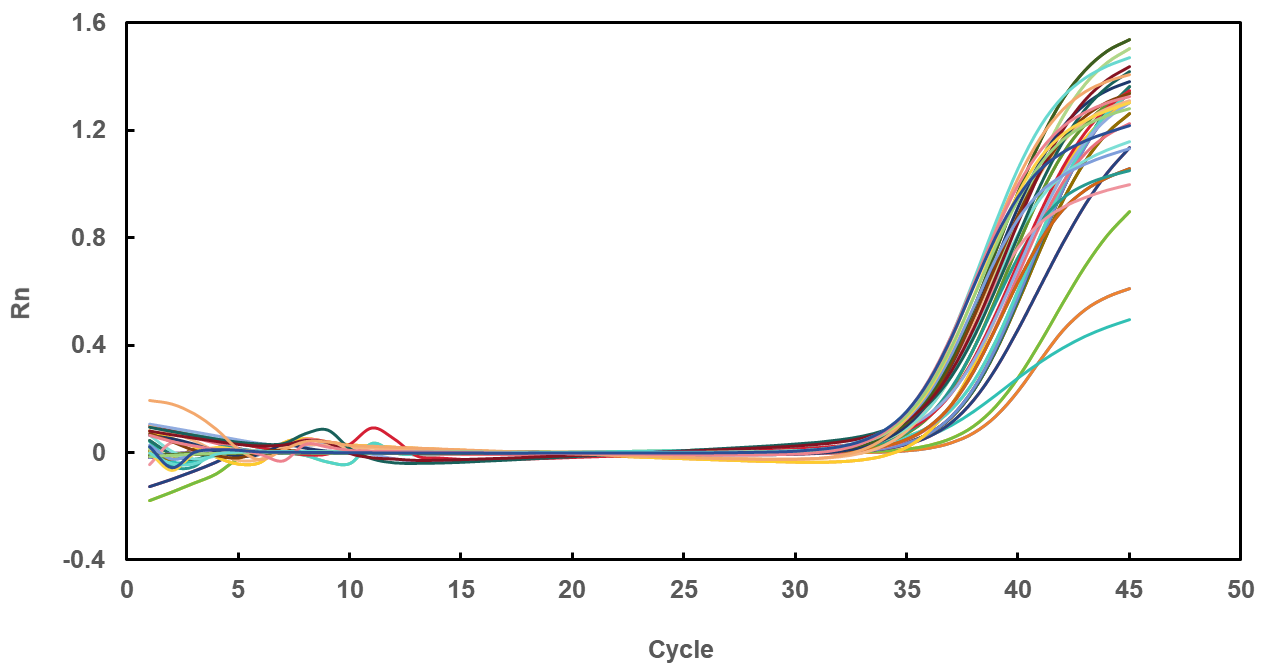


Figure S12. PCR curves from the first round of testing.

22.2. Second-round recommended schemes and autonomous PCR testing results

Table S14. Optimization schemes and PCR testing results from the second iteration (I)

| No. | Guanidine isothiocyanate (g) | Sodium acetate (g) | PEG 8000 (g) | SDS (g) | Isopropanol (g) | Phenol (wt%) | EDTA (wt%) | Tris-HCl (wt%) | Particle diameter (nm) | Carboxyl | Predicted Ct | Tested Ct |
| --- | --- | --- | --- | --- | --- | --- | --- | --- | --- | --- | --- | --- |
| 1 | 60.007 | 5.239 | 46.544 | 30.005 | 240.002 | 0.1955 | 0.0374 | 0.0087 | 700 | 1 | 31.655 | 32.58 |
| 2 | 60.005 | 3.406 | 78.829 | 62.532 | 240.002 | 0.1955 | 0.0403 | 0.0139 | 700 | 1 | 31.694 | 32.07 |
| 3 | 137.678 | 5.197 | 67.096 | 120.005 | 240.004 | 0.2288 | 0.0271 | 0.0146 | 700 | 1 | 31.718 | 33.77 |
| 4 | 174.328 | 8.822 | 49.428 | 69.743 | 221.032 | 0.2491 | 0.0417 | 0.0156 | 600 | 1 | 31.735 | 31.68 |
| 5 | 87.757 | 3.501 | 21.388 | 34.549 | 225.781 | 0.3346 | 0.0292 | 0.0096 | 700 | 1 | 31.773 | 33.54 |
| 6 | 165.198 | 8.406 | 69.448 | 120.002 | 179.538 | 0.2482 | 0.0291 | 0.0104 | 600 | 1 | 31.844 | 31.52 |
| 7 | 60.003 | 12.004 | 80.006 | 120.006 | 240.006 | 0.1955 | 0.0285 | 0.0084 | 600 | 1 | 31.874 | 34.077 |
| 8 | 240.001 | 3.008 | 45.508 | 37.255 | 199.251 | 0.3108 | 0.0426 | 0.0167 | 600 | 1 | 31.877 | 32.78 |
| 9 | 124.286 | 7.044 | 71.129 | 89.204 | 176.479 | 0.2627 | 0.0283 | 0.0136 | 600 | 1 | 31.886 | 33.08 |
| 10 | 194.518 | 7.857 | 55.205 | 116.873 | 169.266 | 0.2576 | 0.0262 | 0.0165 | 600 | 1 | 31.891 | 31.41 |
| 11 | 102.831 | 5.938 | 42.793 | 74.334 | 221.166 | 0.3796 | 0.0424 | 0.0186 | 600 | 1 | 31.934 | 32.16 |
| 12 | 240.008 | 8.757 | 66.904 | 120.002 | 157.921 | 0.2368 | 0.0224 | 0.0085 | 600 | 1 | 31.932 | 33.25 |
| 13 | 96.359 | 11.105 | 80.006 | 82.106 | 195.304 | 0.1955 | 0.0425 | 0.0153 | 700 | 1 | 31.939 | 33.59 |
| 14 | 240.008 | 4.186 | 70.792 | 120.006 | 196.436 | 0.3056 | 0.0425 | 0.0147 | 700 | 1 | 31.946 | 32.68 |
| 15 | 117.977 | 10.745 | 80.004 | 100.606 | 240.003 | 0.1955 | 0.0425 | 0.0148 | 700 | 1 | 31.975 | 34.76 |
| 16 | 188.432 | 8.186 | 62.934 | 116.757 | 173.209 | 0.2524 | 0.0271 | 0.0099 | 600 | 1 | 31.991 | 32.63 |
| 17 | 206.442 | 3.672 | 78.948 | 120.005 | 229.604 | 0.3748 | 0.0253 | 0.0186 | 700 | 1 | 32.013 | 32.23 |
| 18 | 240.007 | 7.528 | 52.043 | 35.641 | 240.005 | 0.4204 | 0.0425 | 0.0171 | 600 | 1 | 32.021 | 33.57 |
| 19 | 240.005 | 8.304 | 80.003 | 80.034 | 186.251 | 0.4204 | 0.0269 | 0.0185 | 700 | 1 | 32.033 | 32.04 |
| 20 | 60.001 | 3.267 | 80.002 | 30.002 | 169.712 | 0.4204 | 0.0426 | 0.0187 | 600 | 1 | 32.043 | 33.85 |
| 21 | 208.025 | 7.637 | 77.561 | 81.402 | 240.008 | 0.2916 | 0.0426 | 0.0159 | 700 | 1 | 32.059 | 32.44 |
| 22 | 78.798 | 9.497 | 80.009 | 49.292 | 183.601 | 0.3956 | 0.0426 | 0.0187 | 700 | 1 | 32.065 | 32.03 |
| 23 | 60.002 | 11.213 | 77.911 | 30.002 | 124.978 | 0.1955 | 0.0425 | 0.0187 | 700 | 1 | 32.071 | 32.44 |
| 24 | 200.747 | 8.794 | 20.008 | 40.818 | 199.674 | 0.2346 | 0.0258 | 0.0155 | 600 | 1 | 32.077 | 34.58 |
| 25 | 60.003 | 9.064 | 50.317 | 111.947 | 106.104 | 0.2516 | 0.0231 | 0.0107 | 600 | 1 | 32.095 | 31.32 |
| 26 | 133.221 | 11.908 | 80.006 | 30.008 | 117.232 | 0.2337 | 0.0411 | 0.0084 | 600 | 1 | 32.104 | 33.54 |
| 27 | 240.001 | 3.004 | 20.002 | 120.002 | 240.008 | 0.1955 | 0.0196 | 0.0176 | 600 | 1 | 32.107 | 31.94 |
| 28 | 240.006 | 3.002 | 20.007 | 120.007 | 240.006 | 0.1955 | 0.0196 | 0.0187 | 600 | 1 | 32.107 | 33.26 |
| 29 | 137.768 | 10.812 | 80.007 | 30.006 | 145.322 | 0.4204 | 0.0427 | 0.0187 | 700 | 1 | 32.145 | 34.57 |
| 30 | 234.038 | 3.009 | 78.272 | 120.006 | 122.099 | 0.3951 | 0.0427 | 0.0102 | 700 | 1 | 32.209 | 34.64 |
| 31 | 196.034 | 11.616 | 74.176 | 120.006 | 195.796 | 0.2454 | 0.0397 | 0.0187 | 700 | 1 | 32.265 | 32.23 |
| 32 | 240.004 | 4.738 | 20.006 | 120.007 | 240.002 | 0.4204 | 0.0426 | 0.0156 | 700 | 1 | 32.298 | 31.46 |

Table S15. Optimization schemes and PCR testing results from the second iteration (II)

| No. | Guanidine isothiocyanate (g) | Ammonium sulphate (g) | PEG 2000 (g) | Triton X-100 (g) | Acetone (g) | Methanol (wt%) | DTPA (wt%) | Tris-HCl (wt%) | Particle diameter (nm) | Carboxyl | Predicted Ct | Tested Ct |
| --- | --- | --- | --- | --- | --- | --- | --- | --- | --- | --- | --- | --- |
| 33 | 233.767 | 8.453 | 20.142 | 120.003 | 229.513 | 0.4081 | 0.0416 | 0.0113 | 600 | 1 | 32.306 | 32.78 |
| 34 | 64.384 | 8.756 | 46.344 | 120.007 | 88.563 | 0.4204 | 0.0425 | 0.0169 | 700 | 1 | 32.356 | 31.87 |
| 35 | 239.208 | 11.777 | 56.443 | 31.962 | 69.088 | 0.1955 | 0.0342 | 0.0119 | 700 | 1 | 32.408 | 32.68 |
| 36 | 240.007 | 3.688 | 20.003 | 120.004 | 169.415 | 0.3337 | 0.0196 | 0.0178 | 600 | 1 | 32.418 | 32.14 |
| 37 | 234.908 | 10.644 | 63.304 | 51.239 | 106.092 | 0.2046 | 0.0355 | 0.0094 | 800 | 1 | 32.465 | 31.62 |
| 38 | 195.458 | 3.007 | 20.008 | 120.005 | 78.778 | 0.3232 | 0.0208 | 0.0143 | 600 | 1 | 32.513 | 32.24 |
| 39 | 240.003 | 12.003 | 80.004 | 120.002 | 60.001 | 0.3711 | 0.0425 | 0.0187 | 700 | 1 | 32.672 | 34.02 |
| 40 | 131.079 | 5.641 | 20.845 | 47.896 | 188.042 | 0.3732 | 0.0332 | 0.0173 | 900 | 2 | 32.877 | 33.35 |

Table S16. Optimization schemes and PCR testing results from the second iteration (III)

| No. | Guanidine isothiocyanate (g) | Sodium acetate (g) | PEG 8000 (g) | Triton X-100 (g) | Isopropanol (g) | Chloroform (wt%) | EGTA (wt%) | HEPES (wt%) | Particle diameter (nm) | Carboxyl | Predicted Ct | Tested Ct |
| --- | --- | --- | --- | --- | --- | --- | --- | --- | --- | --- | --- | --- |
| 41 | 211.611 | 7.048 | 43.709 | 113.399 | 190.909 | 0.2697 | 0.0324 | 0.0134 | 1000 | 2 | 32.934 | 33.43 |
| 42 | 131.975 | 3.423 | 78.425 | 50.954 | 76.309 | 0.3346 | 0.0282 | 0.0179 | 800 | 2 | 32.973 | 33.52 |
| 43 | 70.154 | 9.498 | 76.313 | 30.075 | 238.598 | 0.3344 | 0.0333 | 0.0084 | 600 | 2 | 33.053 | 34.44 |
| 44 | 60.002 | 7.374 | 24.148 | 57.206 | 195.452 | 0.4204 | 0.0246 | 0.0185 | 1000 | 1 | 29.527 | 34.25 |
| 45 | 203.378 | 4.651 | 66.782 | 83.716 | 140.256 | 0.2187 | 0.0299 | 0.0116 | 700 | 2 | 33.134 | 32.22 |
| 46 | 182.455 | 7.054 | 20.796 | 114.798 | 161.392 | 0.2822 | 0.0199 | 0.0106 | 700 | 2 | 33.161 | 32.91 |
| 47 | 213.007 | 7.045 | 25.725 | 63.374 | 180.392 | 0.3453 | 0.0329 | 0.0116 | 800 | 1 | 29.327 | 34.79 |
| 48 | 215.118 | 9.948 | 23.031 | 93.592 | 97.488 | 0.1955 | 0.0266 | 0.0093 | 600 | 2 | 34.320 | 34.37 |


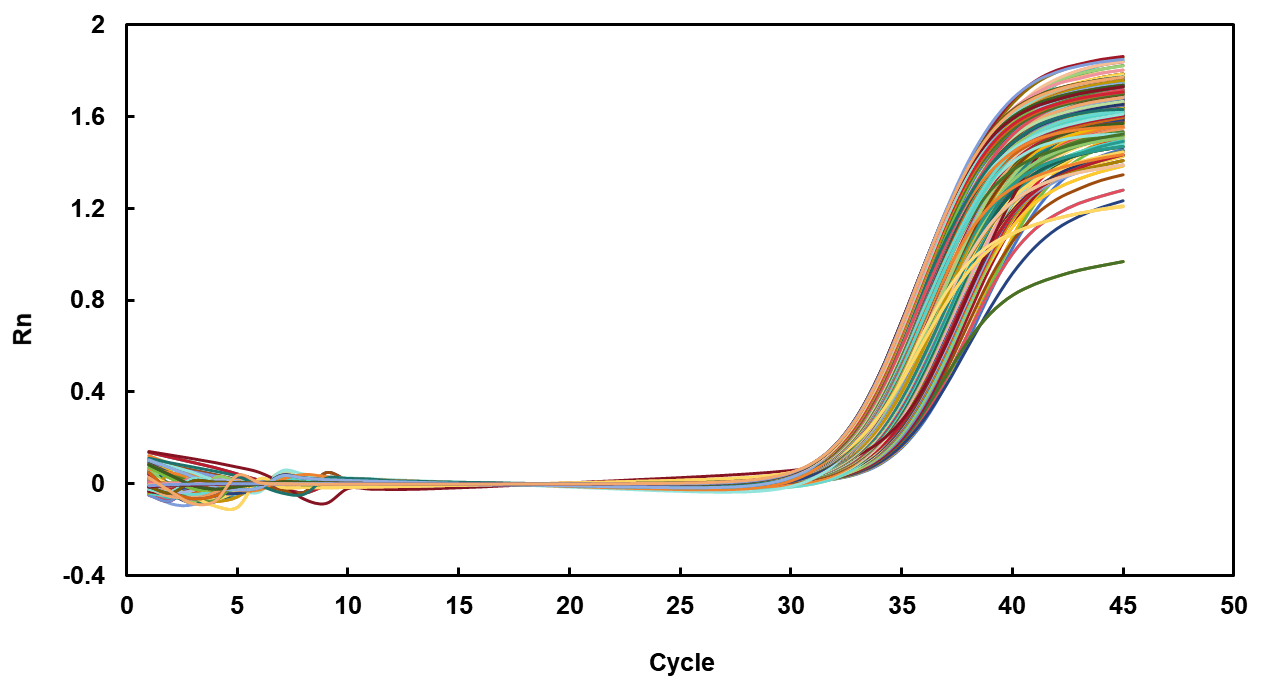


Figure S13. PCR curves from the second round of testing.

22.3. Third-round recommended schemes and autonomous PCR testing results

Table S17. Optimization schemes and PCR testing results from the third iteration (I)

| No. | Guanidine isothiocyanate (g) | Ammonium sulphate (g) | PEG 2000 (g) | Tween-20 (g) | Ethanol (g) | Phenol (wt%) | DTPA (wt%) | HEPES (wt%) | Particle diameter (nm) | Carboxyl | Predicted Ct | Tested Ct |
| --- | --- | --- | --- | --- | --- | --- | --- | --- | --- | --- | --- | --- |
| 1 | 60.008 | 7.374 | 24.148 | 57.206 | 195.452 | 0.4204 | 0.0246 | 0.0185 | 1000 | 1 | 29.527 | 31.27 |
| 2 | 213.007 | 7.045 | 25.725 | 63.374 | 180.392 | 0.3453 | 0.0329 | 0.0117 | 800 | 1 | 29.327 | 32.25 |
| 3 | 60.001 | 5.239 | 46.544 | 30.003 | 240.004 | 0.1955 | 0.0374 | 0.0087 | 700 | 1 | 31.655 | 31.19 |
| 4 | 60.001 | 3.406 | 78.829 | 62.532 | 240.007 | 0.1955 | 0.0403 | 0.0139 | 700 | 1 | 31.694 | 31.72 |
| 5 | 137.678 | 5.197 | 67.096 | 120.005 | 240.006 | 0.2288 | 0.0271 | 0.0146 | 700 | 1 | 31.718 | 30.98 |
| 6 | 174.324 | 8.822 | 49.428 | 69.743 | 221.032 | 0.2491 | 0.0417 | 0.0157 | 600 | 1 | 31.735 | 32.28 |
| 7 | 87.757 | 3.501 | 21.388 | 34.549 | 225.781 | 0.3346 | 0.0292 | 0.0097 | 700 | 1 | 31.773 | 31.33 |
| 8 | 165.198 | 8.406 | 69.448 | 120.008 | 179.538 | 0.2482 | 0.0291 | 0.0104 | 600 | 1 | 31.844 | 30.56 |
| 9 | 60.006 | 12.002 | 80.006 | 120.003 | 240.004 | 0.1955 | 0.0287 | 0.0084 | 600 | 1 | 31.874 | 31.36 |
| 10 | 240.001 | 3.001 | 45.508 | 37.255 | 199.257 | 0.3108 | 0.0428 | 0.0167 | 600 | 1 | 31.877 | 31.08 |
| 11 | 124.286 | 7.044 | 71.129 | 89.204 | 176.479 | 0.2627 | 0.0283 | 0.0136 | 600 | 1 | 31.886 | 31.68 |
| 12 | 194.518 | 7.857 | 55.205 | 116.873 | 169.266 | 0.2576 | 0.0262 | 0.0167 | 600 | 1 | 31.891 | 31.39 |
| 13 | 102.831 | 5.938 | 42.793 | 74.334 | 221.166 | 0.3796 | 0.0425 | 0.0187 | 600 | 1 | 31.934 | 30.75 |
| 14 | 240.009 | 8.757 | 66.904 | 120.007 | 157.921 | 0.2368 | 0.0224 | 0.0085 | 600 | 1 | 31.932 | 32.14 |
| 15 | 96.359 | 11.106 | 80.001 | 82.106 | 195.304 | 0.1955 | 0.0427 | 0.0153 | 700 | 1 | 31.939 | 31.79 |
| 16 | 240.008 | 4.182 | 70.799 | 120.006 | 196.436 | 0.3056 | 0.0428 | 0.0147 | 700 | 1 | 31.946 | 32.32 |
| 17 | 117.977 | 10.745 | 80.001 | 100.606 | 240.002 | 0.1955 | 0.0428 | 0.0148 | 700 | 1 | 31.975 | 30.33 |
| 18 | 188.432 | 8.186 | 62.934 | 116.757 | 173.209 | 0.2524 | 0.0271 | 0.0099 | 600 | 1 | 31.991 | 30.35 |
| 19 | 206.442 | 3.672 | 78.948 | 120.005 | 229.604 | 0.3748 | 0.0253 | 0.0185 | 700 | 1 | 32.013 | 30.21 |
| 20 | 240.001 | 7.528 | 52.043 | 35.641 | 240.005 | 0.4204 | 0.0427 | 0.0171 | 600 | 1 | 32.021 | 30.73 |
| 21 | 240.006 | 8.304 | 80.007 | 80.034 | 186.259 | 0.4204 | 0.0269 | 0.0185 | 700 | 1 | 32.033 | 30.34 |
| 22 | 60.002 | 3.267 | 80.006 | 30.001 | 169.712 | 0.4204 | 0.0427 | 0.0185 | 600 | 1 | 32.043 | 31.02 |
| 23 | 208.025 | 7.637 | 77.561 | 81.402 | 240.004 | 0.2916 | 0.0427 | 0.0159 | 700 | 1 | 32.059 | 31.45 |
| 24 | 78.798 | 9.497 | 80.009 | 49.292 | 183.601 | 0.3975 | 0.0427 | 0.0185 | 700 | 1 | 32.065 | 31.47 |
| 25 | 60.006 | 11.213 | 77.911 | 30.004 | 124.978 | 0.1955 | 0.0427 | 0.0185 | 700 | 1 | 32.071 | 31.77 |
| 26 | 200.747 | 8.794 | 20.009 | 40.815 | 199.674 | 0.2347 | 0.0258 | 0.0157 | 600 | 1 | 32.077 | 30.93 |
| 27 | 60.007 | 9.064 | 50.318 | 111.947 | 106.104 | 0.2516 | 0.0231 | 0.0107 | 600 | 1 | 32.095 | 30.37 |
| 28 | 133.228 | 11.908 | 80.002 | 30.009 | 117.232 | 0.2337 | 0.0411 | 0.0084 | 600 | 1 | 32.104 | 30.48 |
| 29 | 240.008 | 3.004 | 20.001 | 120.006 | 240.001 | 0.1955 | 0.0196 | 0.0176 | 600 | 1 | 32.107 | 32.24 |
| 30 | 240.009 | 3.002 | 20.005 | 120.007 | 240.002 | 0.1955 | 0.0196 | 0.0185 | 600 | 1 | 32.107 | 30.27 |
| 31 | 137.768 | 10.812 | 80.002 | 30.009 | 145.322 | 0.4204 | 0.0427 | 0.0185 | 700 | 1 | 32.145 | 32.35 |
| 32 | 234.038 | 3.004 | 78.272 | 120.005 | 122.099 | 0.3951 | 0.0427 | 0.0102 | 700 | 1 | 32.209 | 31.49 |

Table S18. Optimization schemes and PCR testing results from the third iteration (II)

| No. | Guanidine isothiocyanate (g) | Sodium acetate (g) | Ficoll 400 (g) | SDS (g) | Isopropanol (g) | Phenol (wt%) | DTPA (wt%) | MOPS (wt%) | Particle diameter (nm) | Carboxyl | Predicted Ct | Tested Ct |
| --- | --- | --- | --- | --- | --- | --- | --- | --- | --- | --- | --- | --- |
| 33 | 196.037 | 11.616 | 74.172 | 120.002 | 195.796 | 0.2454 | 0.0397 | 0.0185 | 700 | 1 | 32.265 | 31.65 |
| 34 | 240.003 | 4.738 | 20.007 | 120.007 | 240.005 | 0.4204 | 0.0427 | 0.0156 | 700 | 1 | 32.298 | 30.22 |
| 35 | 233.767 | 8.453 | 20.142 | 120.007 | 229.513 | 0.4081 | 0.0416 | 0.0113 | 600 | 1 | 32.306 | 30.81 |
| 36 | 64.384 | 8.756 | 46.344 | 120.001 | 88.563 | 0.4204 | 0.0427 | 0.0169 | 700 | 1 | 32.356 | 31.53 |
| 37 | 239.208 | 11.777 | 56.443 | 31.962 | 69.085 | 0.1955 | 0.0342 | 0.0119 | 700 | 1 | 32.408 | 31.86 |
| 38 | 240.009 | 3.688 | 20.007 | 120.008 | 169.415 | 0.3337 | 0.0196 | 0.0156 | 600 | 1 | 32.418 | 31.24 |
| 39 | 234.908 | 10.645 | 63.304 | 51.239 | 106.092 | 0.2046 | 0.0355 | 0.0094 | 800 | 1 | 32.465 | 32.18 |
| 40 | 195.458 | 3.007 | 20.008 | 120.001 | 78.776 | 0.3232 | 0.0208 | 0.0143 | 600 | 1 | 32.513 | 30.25 |
| 41 | 240.006 | 12.007 | 80.003 | 120.002 | 60.005 | 0.3711 | 0.0425 | 0.0186 | 700 | 1 | 32.672 | 31.65 |
| 42 | 131.079 | 5.641 | 20.845 | 47.896 | 188.042 | 0.3732 | 0.0332 | 0.0173 | 900 | 2 | 32.877 | 31.37 |
| 43 | 60.005 | 11.408 | 80.003 | 69.629 | 240.001 | 0.1955 | 0.0425 | 0.0125 | 900 | 1 | 27.511 | 30.41 |
| 44 | 211.611 | 7.048 | 43.709 | 113.399 | 190.909 | 0.2697 | 0.0324 | 0.0134 | 1000 | 2 | 32.934 | 30.73 |
| 45 | 131.975 | 3.424 | 78.425 | 50.954 | 76.309 | 0.3346 | 0.0282 | 0.0179 | 800 | 2 | 32.973 | 31.14 |
| 46 | 70.154 | 9.498 | 76.313 | 30.075 | 238.598 | 0.3344 | 0.0333 | 0.0084 | 600 | 2 | 33.053 | 31.81 |
| 47 | 203.378 | 4.651 | 66.782 | 83.716 | 140.253 | 0.2187 | 0.0299 | 0.0116 | 700 | 2 | 33.134 | 31.76 |
| 48 | 182.455 | 7.054 | 20.796 | 114.798 | 161.392 | 0.2822 | 0.0199 | 0.0106 | 700 | 2 | 33.161 | 31.42 |


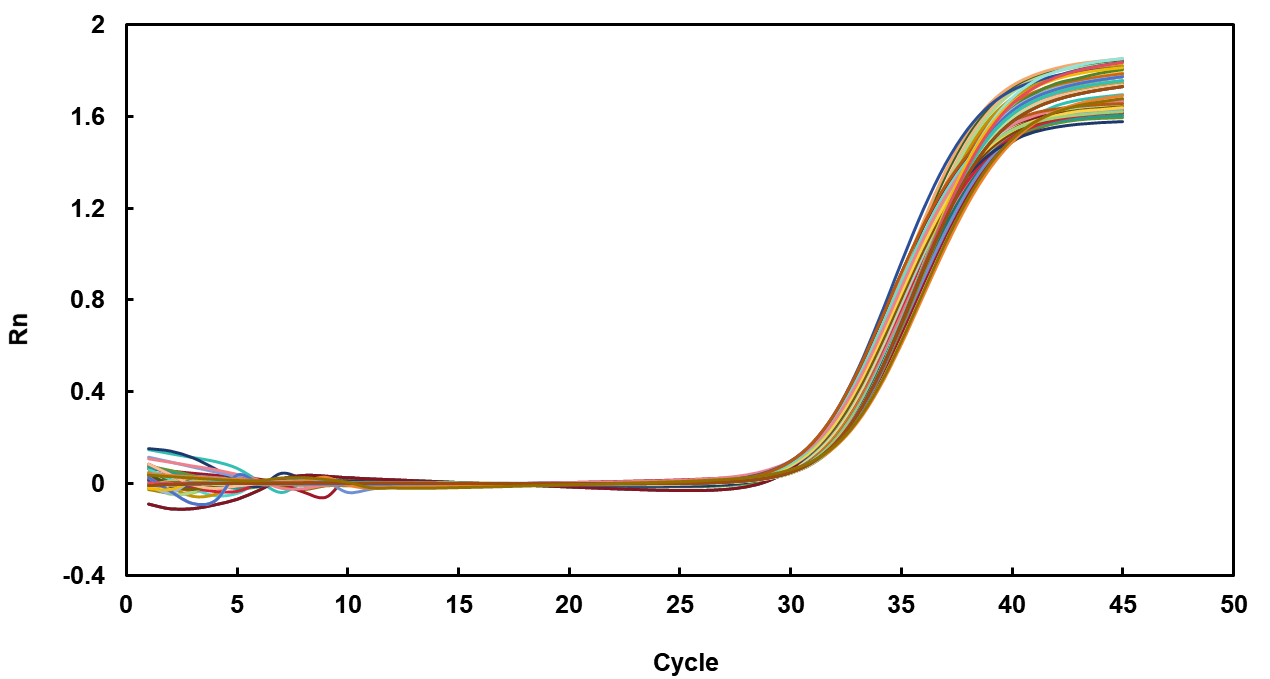


Figure S14. PCR curves from the third round of testing.

22.4. Fourth-round recommended schemes and autonomous PCR testing results

Table S19. Optimization schemes and PCR testing results from the fourth iteration (I)

| No. | Guanidine isothiocyanate (g) | Sodium acetate (g) | PEG 2000 (g) | Triton X-100 (g) | Ethanol (g) | Phenol (wt%) | DTPA (wt%) | HEPES (wt%) | Particle diameter (nm) | Carboxyl | Predicted Ct | Tested Ct |
| --- | --- | --- | --- | --- | --- | --- | --- | --- | --- | --- | --- | --- |
| 1 | 213.007 | 7.045 | 25.725 | 63.374 | 180.392 | 0.3453 | 0.0329 | 0.0115 | 800 | 1 | 29.327 | 31.05 |
| 2 | 60.007 | 11.408 | 80.002 | 69.629 | 240.002 | 0.1955 | 0.0427 | 0.0125 | 900 | 1 | 27.511 | 30.18 |
| 3 | 60.007 | 7.374 | 24.148 | 57.206 | 195.452 | 0.4204 | 0.0246 | 0.0187 | 1000 | 1 | 29.527 | 30.23 |
| 4 | 169.799 | 10.499 | 30.402 | 65.196 | 92.802 | 0.3654 | 0.0291 | 0.0104 | 800 | 1 | 26.904 | 28.71 |
| 5 | 60.007 | 5.239 | 46.544 | 30.007 | 240.009 | 0.1955 | 0.0374 | 0.0087 | 700 | 1 | 31.655 | 30.67 |
| 6 | 60.006 | 3.406 | 78.829 | 62.532 | 240.003 | 0.1955 | 0.0403 | 0.0139 | 700 | 1 | 31.694 | 30.02 |
| 7 | 137.678 | 5.197 | 67.096 | 120.008 | 240.001 | 0.2288 | 0.0271 | 0.0146 | 700 | 1 | 31.718 | 31.29 |
| 8 | 174.326 | 8.822 | 49.428 | 69.743 | 221.032 | 0.2491 | 0.0417 | 0.0157 | 600 | 1 | 31.735 | 29.19 |
| 9 | 87.757 | 3.501 | 21.388 | 34.549 | 225.781 | 0.3346 | 0.0292 | 0.0097 | 700 | 1 | 31.773 | 29.42 |
| 10 | 165.198 | 8.406 | 69.448 | 120.004 | 179.538 | 0.2482 | 0.0291 | 0.0104 | 600 | 1 | 31.844 | 31.4 |
| 11 | 60.002 | 12.004 | 80.008 | 120.008 | 240.004 | 0.1955 | 0.0287 | 0.0084 | 600 | 1 | 31.874 | 29.27 |
| 12 | 240.005 | 3.001 | 45.508 | 37.255 | 199.258 | 0.3108 | 0.0428 | 0.0167 | 600 | 1 | 31.877 | 30.07 |
| 13 | 124.286 | 7.044 | 71.129 | 89.204 | 176.479 | 0.2627 | 0.0283 | 0.0136 | 600 | 1 | 31.886 | 30.19 |
| 14 | 194.518 | 7.857 | 55.205 | 116.873 | 169.266 | 0.2576 | 0.0262 | 0.0156 | 600 | 1 | 31.891 | 29.09 |
| 15 | 102.831 | 5.938 | 42.793 | 74.334 | 221.166 | 0.3796 | 0.0427 | 0.0187 | 600 | 1 | 31.935 | 28.73 |
| 16 | 240.006 | 8.757 | 66.904 | 120.008 | 157.921 | 0.2368 | 0.0224 | 0.0085 | 600 | 1 | 31.932 | 29.65 |
| 17 | 96.359 | 11.106 | 80.004 | 82.106 | 195.304 | 0.1955 | 0.0427 | 0.0153 | 700 | 1 | 31.939 | 31.05 |
| 18 | 240.009 | 4.182 | 70.792 | 120.003 | 196.436 | 0.3056 | 0.0427 | 0.0147 | 700 | 1 | 31.946 | 30.58 |
| 19 | 117.977 | 10.745 | 80.001 | 100.606 | 240.003 | 0.1955 | 0.0425 | 0.0148 | 700 | 1 | 31.975 | 30.45 |
| 20 | 188.432 | 8.186 | 62.934 | 116.757 | 173.209 | 0.2524 | 0.0271 | 0.0099 | 600 | 1 | 31.991 | 29.37 |
| 21 | 206.442 | 3.672 | 78.948 | 120.006 | 229.604 | 0.3748 | 0.0253 | 0.0186 | 700 | 1 | 32.013 | 31.31 |
| 22 | 240.001 | 7.528 | 52.043 | 35.641 | 240.001 | 0.4204 | 0.0426 | 0.0171 | 600 | 1 | 32.021 | 29.36 |
| 23 | 240.003 | 8.304 | 80.002 | 80.034 | 186.257 | 0.4204 | 0.0269 | 0.0186 | 700 | 1 | 32.033 | 31.21 |
| 24 | 60.004 | 3.267 | 80.005 | 30.009 | 169.712 | 0.4204 | 0.0427 | 0.0184 | 600 | 1 | 32.043 | 30.46 |
| 25 | 208.025 | 7.637 | 77.561 | 81.402 | 240.006 | 0.2916 | 0.0427 | 0.0159 | 700 | 1 | 32.059 | 31.39 |
| 26 | 78.798 | 9.497 | 80.003 | 49.292 | 183.601 | 0.3957 | 0.0427 | 0.0184 | 700 | 1 | 32.065 | 28.71 |
| 27 | 60.009 | 11.213 | 77.911 | 30.003 | 124.978 | 0.1955 | 0.0427 | 0.0184 | 700 | 1 | 32.071 | 30.22 |
| 28 | 200.747 | 8.794 | 20.001 | 40.814 | 199.674 | 0.2347 | 0.0258 | 0.0155 | 600 | 1 | 32.077 | 30.3 |
| 29 | 60.002 | 9.064 | 50.315 | 111.947 | 106.104 | 0.2516 | 0.0231 | 0.0107 | 600 | 1 | 32.095 | 29.57 |
| 30 | 133.226 | 11.908 | 80.004 | 30.007 | 117.232 | 0.2337 | 0.0411 | 0.0084 | 600 | 1 | 32.104 | 28.96 |
| 31 | 240.001 | 3.006 | 20.001 | 120.006 | 240.004 | 0.1955 | 0.0196 | 0.0184 | 600 | 1 | 32.107 | 29.72 |
| 32 | 240.007 | 3.003 | 20.006 | 120.007 | 240.001 | 0.1955 | 0.0196 | 0.0176 | 600 | 1 | 32.107 | 29.04 |
| 33 | 137.768 | 10.812 | 80.001 | 30.007 | 145.322 | 0.4204 | 0.0427 | 0.0184 | 700 | 1 | 32.145 | 31.08 |
| 34 | 234.038 | 3.006 | 78.272 | 120.005 | 122.099 | 0.3951 | 0.0427 | 0.0102 | 700 | 1 | 32.209 | 28.85 |
| 35 | 196.032 | 11.616 | 74.178 | 120.002 | 195.796 | 0.2454 | 0.0397 | 0.0184 | 700 | 1 | 32.265 | 30.97 |
| 36 | 240.004 | 4.738 | 20.008 | 120.007 | 240.004 | 0.4204 | 0.0426 | 0.0156 | 700 | 1 | 32.298 | 28.54 |

Table S20. Optimization schemes and PCR testing results from the fourth iteration (II)

| No. | Guanidine isothiocyanate (g) | Sodium acetate (g) | PEG 2000 (g) | SDS (g) | Isopropanol (g) | Methanol (wt%) | EDTA (wt%) | Tris-HCl (wt%) | Particle diameter (nm) | Carboxyl | Predicted Ct | Tested Ct |
| --- | --- | --- | --- | --- | --- | --- | --- | --- | --- | --- | --- | --- |
| 37 | 233.767 | 8.453 | 20.142 | 120.009 | 229.513 | 0.4081 | 0.0416 | 0.0113 | 600 | 1 | 32.306 | 30.19 |
| 38 | 64.384 | 8.756 | 46.344 | 120.001 | 88.563 | 0.4204 | 0.0426 | 0.0169 | 700 | 1 | 32.356 | 29.18 |
| 39 | 239.208 | 11.777 | 56.443 | 31.962 | 69.082 | 0.1955 | 0.0342 | 0.0119 | 700 | 1 | 32.408 | 29.96 |
| 40 | 240.009 | 3.688 | 20.004 | 120.007 | 169.415 | 0.3337 | 0.0196 | 0.0156 | 600 | 1 | 32.418 | 28.67 |
| 41 | 234.908 | 10.644 | 63.304 | 51.239 | 106.092 | 0.2046 | 0.0355 | 0.0094 | 800 | 1 | 32.465 | 30.92 |
| 42 | 195.458 | 3.006 | 20.001 | 120.008 | 78.773 | 0.3232 | 0.0208 | 0.0143 | 600 | 1 | 32.513 | 29.83 |
| 43 | 240.004 | 12.008 | 80.002 | 120.002 | 60.003 | 0.3711 | 0.0426 | 0.0186 | 700 | 1 | 32.672 | 28.95 |
| 44 | 131.079 | 5.641 | 20.845 | 47.896 | 188.042 | 0.3732 | 0.0332 | 0.0173 | 800 | 2 | 32.877 | 30.68 |
| 45 | 211.611 | 7.048 | 43.709 | 113.399 | 190.909 | 0.2696 | 0.0324 | 0.0134 | 1000 | 2 | 32.934 | 31.21 |
| 46 | 131.975 | 3.426 | 78.425 | 50.957 | 76.309 | 0.3346 | 0.0282 | 0.0179 | 800 | 2 | 32.973 | 29.12 |
| 47 | 70.154 | 9.498 | 76.313 | 30.077 | 238.598 | 0.3344 | 0.0333 | 0.0084 | 600 | 2 | 33.053 | 29.21 |
| 48 | 203.378 | 4.651 | 66.782 | 83.716 | 140.253 | 0.2186 | 0.0299 | 0.0116 | 600 | 2 | 33.134 | 28.96 |


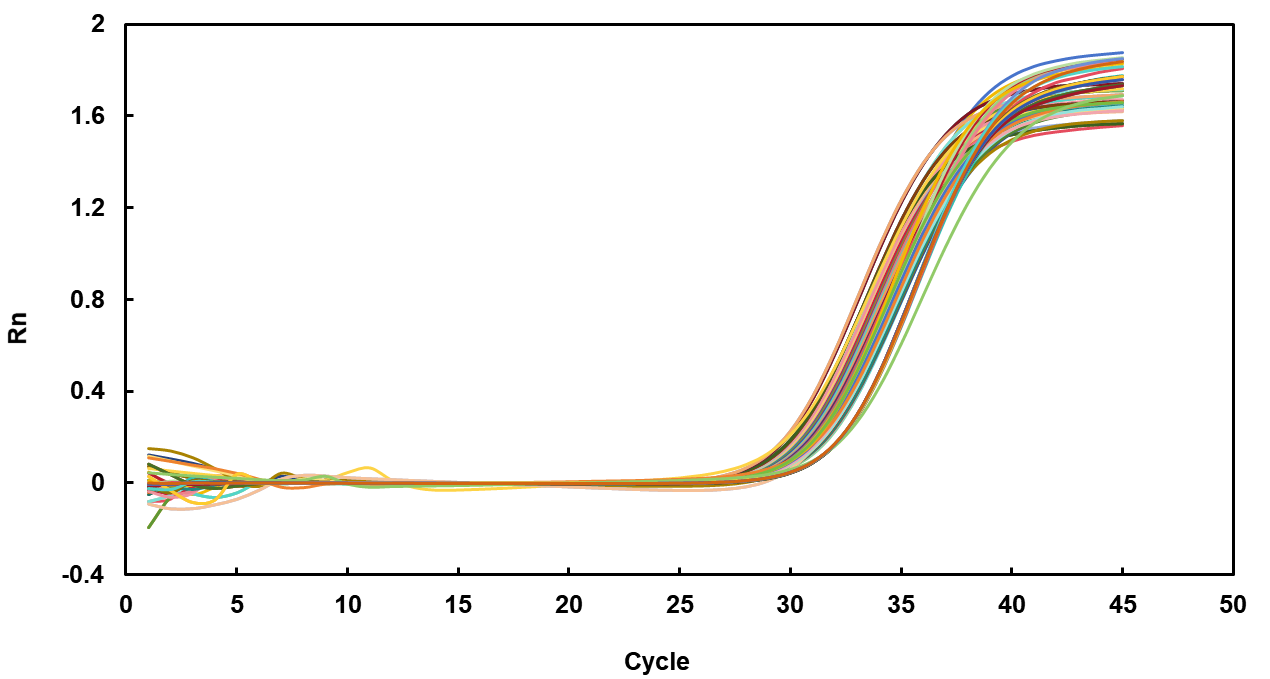


Figure S15. PCR curves from the fourth round of testing.

22.5. Fifth-round recommended schemes and autonomous PCR testing results

Table S21. Optimization schemes and PCR testing results from the fifth iteration (I)

| No. | Guanidine isothiocyanate (g) | Potassium chloride (g) | Ficoll 400 (g) | Triton X-100 (g) | Ethanol (g) | Methanol (wt%) | EGTA (wt%) | Tris-HCl (wt%) | Particle diameter (nm) | Carboxyl | Predicted Ct | Tested Ct |
| --- | --- | --- | --- | --- | --- | --- | --- | --- | --- | --- | --- | --- |
| 1 | 60.004 | 11.408 | 80.008 | 69.629 | 240.009 | 0.1955 | 0.0426 | 0.0125 | 900 | 1 | 27.511 | 29.06 |
| 2 | 169.799 | 10.499 | 30.402 | 65.196 | 92.802 | 0.3654 | 0.0291 | 0.0104 | 800 | 1 | 27.904 | 29.17 |
| 3 | 213.007 | 7.045 | 25.725 | 63.374 | 180.392 | 0.3453 | 0.0329 | 0.0116 | 800 | 1 | 29.327 | 28.16 |
| 4 | 60.006 | 7.374 | 24.148 | 57.206 | 195.452 | 0.4204 | 0.0246 | 0.0186 | 1000 | 1 | 29.527 | 29.35 |
| 5 | 60.002 | 5.239 | 46.544 | 30.002 | 240.008 | 0.1955 | 0.0374 | 0.0087 | 700 | 1 | 31.655 | 28.16 |
| 6 | 60.003 | 3.406 | 78.829 | 62.532 | 240.003 | 0.1955 | 0.0403 | 0.0139 | 700 | 1 | 31.694 | 29.27 |
| 7 | 137.678 | 5.197 | 67.096 | 120.007 | 240.007 | 0.2288 | 0.0271 | 0.0146 | 700 | 1 | 31.718 | 28.45 |
| 8 | 174.322 | 8.822 | 49.428 | 69.743 | 221.032 | 0.2491 | 0.0417 | 0.0156 | 600 | 1 | 31.735 | 27.84 |
| 9 | 87.757 | 3.501 | 21.388 | 34.549 | 225.781 | 0.3346 | 0.0292 | 0.0096 | 700 | 1 | 31.773 | 28.25 |
| 10 | 165.198 | 8.406 | 69.448 | 120.006 | 179.538 | 0.2482 | 0.0291 | 0.0104 | 600 | 1 | 31.844 | 29.21 |
| 11 | 60.008 | 12.004 | 80.009 | 120.002 | 240.003 | 0.1955 | 0.0286 | 0.0084 | 600 | 1 | 31.874 | 29.29 |
| 12 | 240.008 | 3.001 | 45.508 | 37.255 | 199.255 | 0.3108 | 0.0426 | 0.0167 | 600 | 1 | 31.877 | 29.24 |
| 13 | 124.286 | 7.044 | 71.129 | 89.204 | 176.479 | 0.2627 | 0.0283 | 0.0136 | 600 | 1 | 31.886 | 28.29 |
| 14 | 194.518 | 7.857 | 55.205 | 116.873 | 169.266 | 0.2576 | 0.0262 | 0.0156 | 600 | 1 | 31.891 | 28.14 |
| 15 | 102.831 | 5.938 | 42.793 | 74.334 | 221.166 | 0.3796 | 0.0426 | 0.0186 | 600 | 1 | 31.935 | 28.25 |
| 16 | 240.006 | 8.757 | 66.904 | 120.003 | 157.921 | 0.2368 | 0.0224 | 0.0085 | 600 | 1 | 31.932 | 28.11 |
| 17 | 96.359 | 11.107 | 80.002 | 82.106 | 195.304 | 0.1955 | 0.0426 | 0.0153 | 700 | 1 | 31.939 | 28.29 |
| 18 | 240.002 | 4.183 | 70.791 | 120.003 | 196.436 | 0.3056 | 0.0426 | 0.0147 | 700 | 1 | 31.946 | 28.98 |
| 19 | 117.977 | 10.745 | 80.005 | 100.606 | 240.009 | 0.1955 | 0.0425 | 0.0148 | 700 | 1 | 31.975 | 28.05 |
| 20 | 188.432 | 8.186 | 62.934 | 116.757 | 173.209 | 0.2524 | 0.0271 | 0.0099 | 600 | 1 | 31.991 | 29.18 |
| 21 | 206.442 | 3.672 | 78.948 | 120.002 | 229.604 | 0.3748 | 0.0253 | 0.0184 | 700 | 1 | 32.013 | 28.67 |
| 22 | 240.004 | 7.528 | 52.043 | 35.641 | 240.003 | 0.4204 | 0.0425 | 0.0171 | 600 | 1 | 32.021 | 28.23 |
| 23 | 240.001 | 8.304 | 80.008 | 80.034 | 186.251 | 0.4204 | 0.0269 | 0.0184 | 700 | 1 | 32.033 | 27.78 |
| 24 | 60.005 | 3.267 | 80.008 | 30.003 | 169.712 | 0.4204 | 0.0425 | 0.0184 | 600 | 1 | 32.043 | 29.18 |
| 25 | 208.025 | 7.637 | 77.561 | 81.402 | 240.006 | 0.2916 | 0.0425 | 0.0159 | 700 | 1 | 32.059 | 29.18 |
| 26 | 78.798 | 9.497 | 80.005 | 49.292 | 183.601 | 0.3956 | 0.0425 | 0.0184 | 700 | 1 | 32.065 | 29.29 |
| 27 | 60.001 | 11.213 | 77.911 | 30.002 | 124.978 | 0.1955 | 0.0425 | 0.0184 | 700 | 1 | 32.071 | 27.96 |
| 28 | 200.747 | 8.794 | 20.001 | 40.811 | 199.674 | 0.2346 | 0.0258 | 0.0155 | 600 | 1 | 32.077 | 28.04 |
| 29 | 60.002 | 9.064 | 50.311 | 111.947 | 106.104 | 0.2516 | 0.0231 | 0.0107 | 600 | 1 | 32.095 | 29.11 |
| 30 | 133.223 | 11.908 | 80.005 | 30.009 | 117.232 | 0.2337 | 0.0411 | 0.0084 | 600 | 1 | 32.104 | 29.18 |
| 31 | 240.001 | 3.009 | 20.006 | 120.001 | 240.009 | 0.1955 | 0.0196 | 0.0184 | 600 | 1 | 32.107 | 29.05 |
| 32 | 240.003 | 3.001 | 20.004 | 120.009 | 240.005 | 0.1955 | 0.0196 | 0.0176 | 600 | 1 | 32.107 | 29.36 |
| 33 | 137.768 | 10.812 | 80.005 | 30.007 | 145.322 | 0.4204 | 0.0424 | 0.0184 | 700 | 1 | 32.145 | 29.21 |
| 34 | 234.038 | 3.001 | 78.272 | 120.006 | 122.099 | 0.3951 | 0.0424 | 0.0102 | 700 | 1 | 32.209 | 29.08 |
| 35 | 196.039 | 11.616 | 74.175 | 120.008 | 195.796 | 0.2454 | 0.0395 | 0.0184 | 700 | 1 | 32.265 | 29.24 |
| 36 | 240.008 | 4.738 | 20.008 | 120.001 | 240.004 | 0.4204 | 0.0426 | 0.0156 | 700 | 1 | 32.298 | 28.73 |

Table S22. Optimization schemes and PCR testing results from the fifth iteration (II)

| No. | Guanidine isothiocyanate (g) | Potassium chloride (g) | PEG 2000 (g) | Triton X-100 (g) | Acetone (g) | Phenol (wt%) | EDTA (wt%) | HEPES (wt%) | Particle diameter (nm) | Carboxyl | Predicted Ct | Tested Ct |
| --- | --- | --- | --- | --- | --- | --- | --- | --- | --- | --- | --- | --- |
| 37 | 233.767 | 8.453 | 20.142 | 120.007 | 229.513 | 0.4081 | 0.0416 | 0.0113 | 600 | 1 | 32.306 | 29.07 |
| 38 | 64.384 | 8.756 | 46.344 | 120.009 | 88.563 | 0.4204 | 0.0425 | 0.0169 | 700 | 1 | 32.356 | 28.54 |
| 39 | 239.208 | 11.777 | 56.443 | 31.962 | 69.082 | 0.1955 | 0.0342 | 0.0119 | 700 | 1 | 32.408 | 28.27 |
| 40 | 240.005 | 3.688 | 20.004 | 120.007 | 169.415 | 0.3337 | 0.0196 | 0.0145 | 600 | 1 | 32.418 | 28.79 |
| 41 | 234.908 | 10.648 | 63.304 | 51.239 | 106.092 | 0.2046 | 0.0355 | 0.0094 | 800 | 1 | 32.465 | 29.07 |
| 42 | 195.458 | 3.008 | 20.007 | 120.005 | 78.771 | 0.3232 | 0.0208 | 0.0143 | 600 | 1 | 32.513 | 29.14 |
| 43 | 240.003 | 12.008 | 80.004 | 120.002 | 60.009 | 0.3711 | 0.0425 | 0.0185 | 700 | 1 | 32.672 | 29.15 |
| 44 | 131.079 | 5.641 | 20.845 | 47.896 | 188.042 | 0.3732 | 0.0332 | 0.0173 | 800 | 2 | 32.877 | 29.17 |
| 45 | 211.611 | 7.048 | 43.709 | 113.399 | 190.909 | 0.2695 | 0.0324 | 0.0134 | 1000 | 2 | 32.934 | 28.33 |
| 46 | 131.975 | 3.426 | 78.425 | 50.954 | 76.309 | 0.3346 | 0.0282 | 0.0179 | 800 | 2 | 32.973 | 28.33 |
| 47 | 70.154 | 9.498 | 76.313 | 30.072 | 238.598 | 0.3344 | 0.0333 | 0.0084 | 600 | 2 | 33.053 | 27.97 |
| 48 | 203.378 | 4.651 | 66.782 | 83.716 | 140.257 | 0.2185 | 0.0299 | 0.0116 | 600 | 2 | 33.134 | 27.84 |


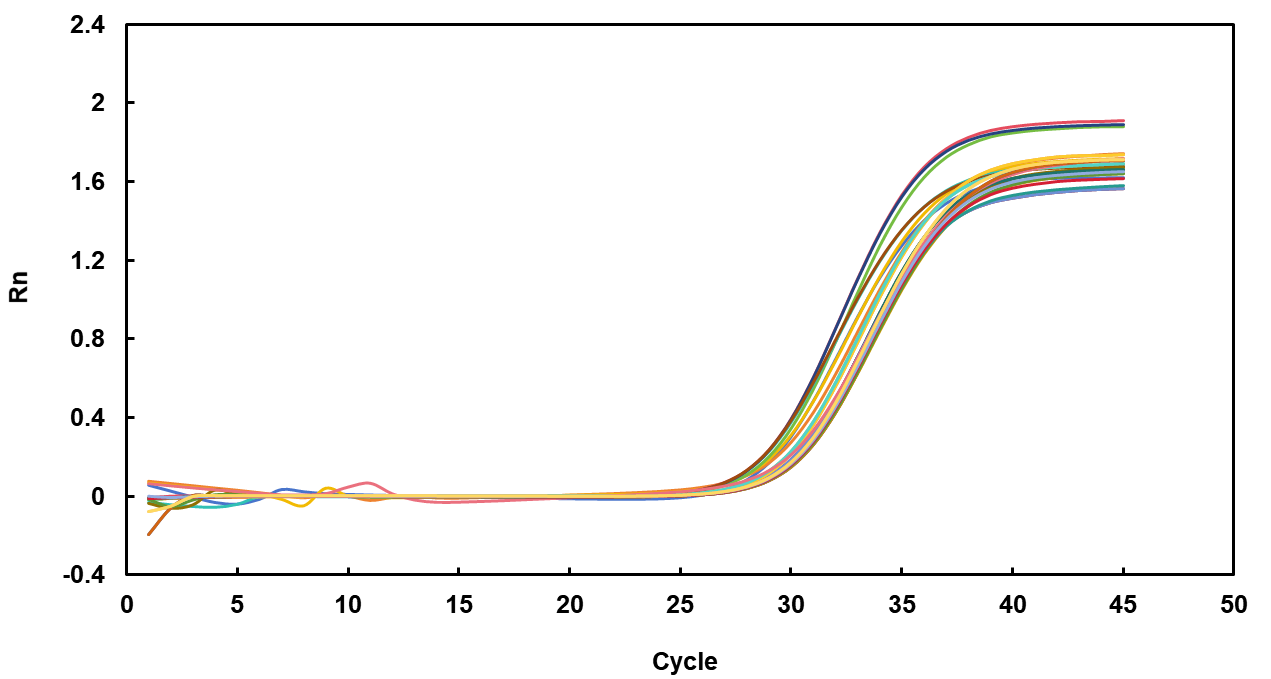


Figure S16. PCR curves from the fifth round of testing.

22.6. Sixth-round recommended schemes and autonomous PCR testing results

Table S23. Optimization schemes and PCR testing results from the sixth iteration (I)

| No. | Guanidine isothiocyanate (g) | Potassium chloride (g) | PEG 8000 (g) | SDS (g) | Ethanol (g) | Phenol (wt%) | EGTA (wt%) | MOPS (wt%) | | Particle diameter (nm) | Carboxyl | Predicted Ct | Tested Ct |
| --- | --- | --- | --- | --- | --- | --- | --- | --- | --- | --- | --- | --- | --- |
| 1 | 60.003 | 11.408 | 80.007 | 69.629 | 240.006 | 0.1955 | 0.0426 | | 0.0125 | 900 | 1 | 27.511 | 27.93 |
| 2 | 169.799 | 10.499 | 30.402 | 65.196 | 92.802 | 0.3654 | 0.0291 | | 0.0104 | 800 | 1 | 26.904 | 27.84 |
| 3 | 213.007 | 7.045 | 25.725 | 63.374 | 180.392 | 0.3453 | 0.0329 | | 0.0116 | 800 | 1 | 27.327 | 27.99 |
| 4 | 60.006 | 7.374 | 24.148 | 57.206 | 195.452 | 0.4204 | 0.0246 | | 0.0186 | 1000 | 1 | 27.527 | 27.89 |
| 5 | 60.006 | 5.239 | 46.544 | 30.005 | 240.006 | 0.1955 | 0.0374 | | 0.0087 | 700 | 1 | 27.655 | 27.64 |
| 6 | 60.007 | 3.406 | 78.829 | 62.532 | 240.003 | 0.1955 | 0.0403 | | 0.0139 | 700 | 1 | 27.694 | 27.86 |
| 7 | 137.678 | 5.197 | 67.096 | 120.009 | 240.007 | 0.2288 | 0.0271 | | 0.0146 | 700 | 1 | 27.718 | 28.17 |
| 8 | 174.322 | 8.822 | 49.428 | 69.743 | 221.032 | 0.2491 | 0.0417 | | 0.0157 | 600 | 1 | 27.735 | 27.55 |
| 9 | 87.757 | 3.501 | 21.388 | 34.549 | 225.781 | 0.3346 | 0.0292 | | 0.0097 | 700 | 1 | 27.773 | 28.03 |
| 10 | 165.198 | 8.406 | 69.448 | 120.009 | 179.538 | 0.2482 | 0.0291 | | 0.0104 | 600 | 1 | 27.844 | 27.57 |
| 11 | 60.006 | 12.002 | 80.009 | 120.003 | 240.006 | 0.1955 | 0.0286 | | 0.0084 | 600 | 1 | 27.874 | 27.71 |
| 12 | 240.007 | 3.004 | 45.508 | 37.255 | 199.255 | 0.3108 | 0.0426 | | 0.0167 | 600 | 1 | 27.877 | 27.66 |
| 13 | 124.286 | 7.044 | 71.129 | 89.204 | 176.479 | 0.2627 | 0.0283 | | 0.0136 | 600 | 1 | 27.886 | 27.43 |
| 14 | 194.518 | 7.857 | 55.205 | 116.873 | 169.266 | 0.2576 | 0.0262 | | 0.0167 | 600 | 1 | 27.891 | 28.25 |
| 15 | 102.831 | 5.938 | 42.793 | 74.334 | 221.166 | 0.3796 | 0.0426 | | 0.0187 | 600 | 1 | 27.934 | 28.03 |
| 16 | 240.002 | 8.757 | 66.904 | 120.001 | 157.921 | 0.2368 | 0.0224 | | 0.0085 | 600 | 1 | 27.932 | 27.93 |
| 17 | 96.359 | 11.104 | 80.001 | 82.106 | 195.304 | 0.1955 | 0.0426 | | 0.0153 | 700 | 1 | 27.939 | 28.16 |
| 18 | 240.001 | 4.182 | 70.794 | 120.008 | 196.436 | 0.3056 | 0.0426 | | 0.0147 | 700 | 1 | 27.946 | 27.63 |
| 19 | 117.977 | 10.745 | 80.005 | 100.606 | 240.009 | 0.1955 | 0.0427 | | 0.0148 | 700 | 1 | 27.975 | 27.44 |
| 20 | 188.432 | 8.186 | 62.934 | 116.757 | 173.209 | 0.2524 | 0.0271 | | 0.0099 | 600 | 1 | 26.991 | 27.59 |
| 21 | 206.442 | 3.672 | 78.948 | 120.008 | 229.604 | 0.3748 | 0.0253 | | 0.0186 | 700 | 1 | 27.013 | 27.81 |
| 22 | 240.009 | 7.528 | 52.043 | 35.641 | 240.008 | 0.4204 | 0.0427 | | 0.0171 | 600 | 1 | 27.021 | 27.47 |
| 23 | 240.009 | 8.304 | 80.005 | 80.034 | 186.255 | 0.4204 | 0.0269 | | 0.0186 | 700 | 1 | 27.033 | 27.86 |
| 24 | 60.007 | 3.267 | 80.004 | 30.001 | 169.712 | 0.4204 | 0.0427 | | 0.0186 | 600 | 1 | 27.043 | 27.95 |
| 25 | 208.025 | 7.637 | 77.561 | 81.402 | 240.008 | 0.2916 | 0.0426 | | 0.0159 | 700 | 1 | 27.059 | 27.72 |
| 26 | 78.798 | 9.497 | 80.006 | 49.292 | 183.601 | 0.3967 | 0.0426 | | 0.0186 | 700 | 1 | 27.065 | 27.75 |
| 27 | 60.007 | 11.213 | 77.911 | 30.008 | 124.978 | 0.1955 | 0.0426 | | 0.0186 | 700 | 1 | 27.071 | 28.11 |
| 28 | 200.747 | 8.794 | 20.003 | 40.818 | 199.674 | 0.2347 | 0.0258 | | 0.0156 | 600 | 1 | 27.077 | 27.97 |
| 29 | 60.007 | 9.064 | 50.317 | 111.947 | 106.104 | 0.2516 | 0.0231 | | 0.0107 | 600 | 1 | 27.095 | 27.95 |
| 30 | 133.226 | 11.908 | 80.006 | 30.001 | 117.232 | 0.2337 | 0.0411 | | 0.0084 | 600 | 1 | 27.104 | 27.54 |
| 31 | 240.006 | 3.005 | 20.009 | 120.003 | 240.001 | 0.1955 | 0.0196 | | 0.0186 | 600 | 1 | 27.107 | 28.19 |
| 32 | 240.006 | 3.008 | 20.004 | 120.001 | 240.009 | 0.1955 | 0.0196 | | 0.0176 | 600 | 1 | 27.107 | 27.76 |
| 33 | 137.768 | 10.812 | 80.001 | 30.007 | 145.322 | 0.4204 | 0.0426 | | 0.0186 | 700 | 1 | 27.145 | 27.65 |
| 34 | 234.038 | 3.001 | 78.272 | 120.009 | 122.099 | 0.3951 | 0.0426 | | 0.0102 | 700 | 1 | 27.209 | 27.89 |
| 35 | 196.032 | 11.616 | 74.177 | 120.004 | 195.796 | 0.2454 | 0.0397 | | 0.0186 | 700 | 1 | 27.265 | 28.05 |
| 36 | 240.003 | 4.738 | 20.008 | 120.003 | 240.008 | 0.4204 | 0.0427 | | 0.0156 | 700 | 1 | 27.298 | 27.93 |

Table S24. Optimization schemes and PCR testing results from the sixth iteration (II)

| No. | Guanidine isothiocyanate (g) | Ammonium sulphate (g) | Ficoll 400 (g) | SDS (g) | Ethanol (g) | Phenol (wt%) | DTPA (wt%) | Tris-HCl (wt%) | Particle diameter (nm) | Carboxyl | Predicted Ct | Tested Ct |
| --- | --- | --- | --- | --- | --- | --- | --- | --- | --- | --- | --- | --- |
| 37 | 233.767 | 8.453 | 20.142 | 120.009 | 229.513 | 0.4081 | 0.0416 | 0.0113 | 600 | 1 | 27.306 | 28.09 |
| 38 | 64.384 | 8.756 | 46.344 | 120.002 | 88.563 | 0.4204 | 0.0427 | 0.0169 | 700 | 1 | 27.356 | 27.58 |
| 39 | 239.208 | 11.777 | 56.443 | 31.962 | 69.087 | 0.1955 | 0.0342 | 0.0119 | 700 | 1 | 27.408 | 27.52 |
| 40 | 240.006 | 3.688 | 20.003 | 120.008 | 169.415 | 0.3337 | 0.0196 | 0.0177 | 600 | 1 | 27.418 | 28.07 |
| 41 | 234.908 | 10.649 | 63.304 | 51.239 | 106.092 | 0.2046 | 0.0355 | 0.0094 | 800 | 1 | 27.465 | 28.02 |
| 42 | 195.458 | 3.006 | 20.005 | 120.008 | 78.774 | 0.3232 | 0.0208 | 0.0143 | 600 | 1 | 27.513 | 27.72 |
| 43 | 240.001 | 12.009 | 80.009 | 120.007 | 60.004 | 0.3711 | 0.0427 | 0.0186 | 700 | 1 | 27.672 | 27.94 |
| 44 | 131.079 | 5.641 | 20.845 | 47.896 | 188.042 | 0.3732 | 0.0332 | 0.0173 | 800 | 2 | 26.877 | 28.11 |
| 45 | 211.611 | 7.048 | 43.709 | 113.399 | 190.909 | 0.2697 | 0.0324 | 0.0134 | 1000 | 2 | 26.934 | 27.77 |
| 46 | 131.975 | 3.423 | 78.425 | 50.952 | 76.309 | 0.3346 | 0.0282 | 0.0179 | 800 | 2 | 26.973 | 27.52 |
| 47 | 70.154 | 9.498 | 76.313 | 30.079 | 238.598 | 0.3344 | 0.0333 | 0.0084 | 600 | 2 | 27.053 | 28.01 |
| 48 | 203.378 | 4.651 | 66.782 | 83.716 | 140.253 | 0.2187 | 0.0299 | 0.0116 | 600 | 2 | 27.134 | 28.11 |


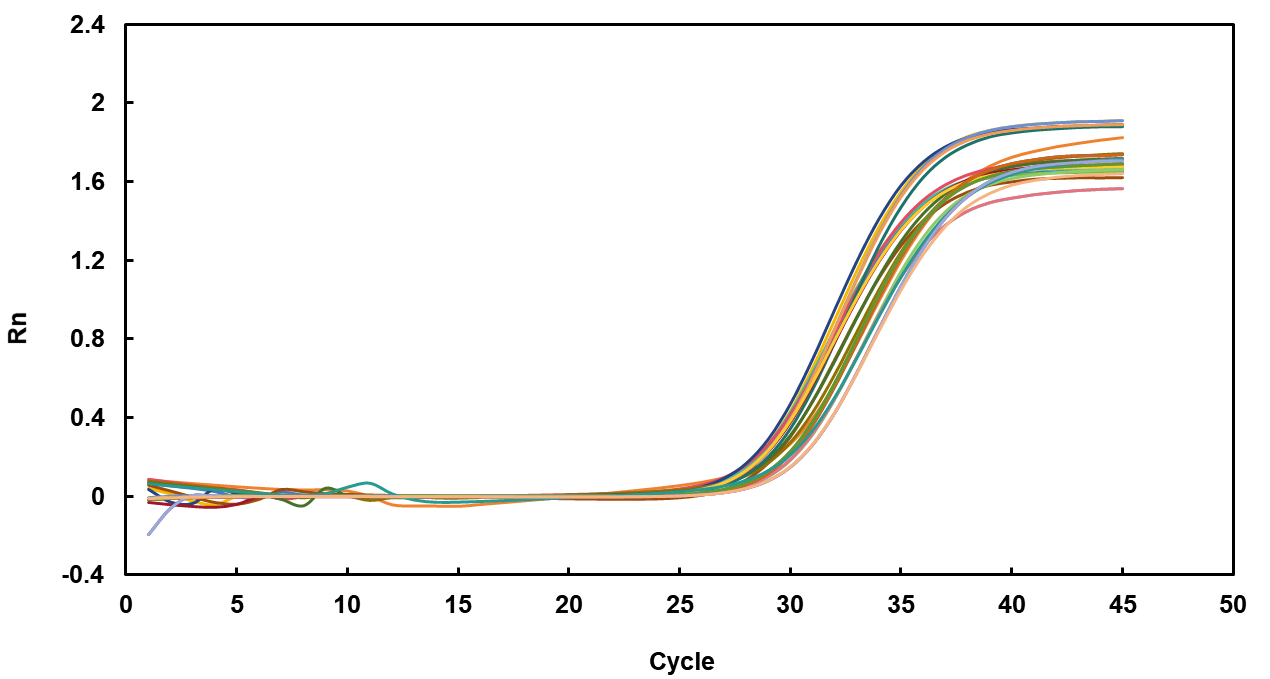


Figure S17. PCR curves from the sixth round of testing.

22.7. Seventh-round recommended schemes and autonomous PCR testing results

Table S25. Optimization schemes and PCR testing results from the seventh iteration (I)

| No. | Guanidine isothiocyanate (g) | Sodium acetate (g) | Ficoll 400 (g) | SDS (g) | Isopropanol (g) | Chloroform (wt%) | DTPA (wt%) | Tris-HCl (wt%) | Particle diameter (nm) | Carboxyl | Predicted Ct | Tested Ct |
| --- | --- | --- | --- | --- | --- | --- | --- | --- | --- | --- | --- | --- |
| 1 | 169.799 | 10.499 | 30.402 | 65.196 | 92.802 | 0.3654 | 0.0291 | 0.0104 | 800 | 1 | 26.904 | 27.83 |
| 2 | 60.006 | 11.408 | 80.001 | 69.629 | 240.003 | 0.1955 | 0.0427 | 0.0125 | 900 | 1 | 27.511 | 27.87 |
| 3 | 213.007 | 7.045 | 25.725 | 63.374 | 180.392 | 0.3453 | 0.0329 | 0.0117 | 800 | 1 | 27.327 | 27.65 |
| 4 | 60.003 | 7.374 | 24.148 | 57.206 | 195.452 | 0.4204 | 0.0246 | 0.0187 | 1000 | 1 | 27.527 | 27.35 |
| 5 | 60.004 | 5.239 | 46.544 | 30.003 | 240.006 | 0.1955 | 0.0374 | 0.0087 | 700 | 1 | 27.655 | 27.22 |
| 6 | 60.003 | 3.406 | 78.829 | 62.532 | 240.007 | 0.1955 | 0.0403 | 0.0139 | 700 | 1 | 27.694 | 27.35 |
| 7 | 137.678 | 5.197 | 67.096 | 120.006 | 240.004 | 0.2288 | 0.0271 | 0.0146 | 700 | 1 | 27.718 | 27.28 |
| 8 | 174.326 | 8.822 | 49.428 | 69.743 | 221.032 | 0.2491 | 0.0417 | 0.0158 | 600 | 1 | 27.735 | 27.32 |
| 9 | 87.757 | 3.501 | 21.388 | 34.549 | 225.781 | 0.3346 | 0.0292 | 0.0098 | 700 | 1 | 26.773 | 27.48 |
| 10 | 165.198 | 8.406 | 69.448 | 120.002 | 179.538 | 0.2482 | 0.0291 | 0.0104 | 600 | 1 | 26.844 | 27.22 |
| 11 | 60.004 | 12.004 | 80.007 | 120.002 | 240.004 | 0.1955 | 0.0286 | 0.0084 | 600 | 1 | 26.874 | 27.87 |
| 12 | 240.002 | 3.004 | 45.508 | 37.255 | 199.252 | 0.3108 | 0.0427 | 0.0167 | 600 | 1 | 26.877 | 27.51 |
| 13 | 124.286 | 7.044 | 71.129 | 89.204 | 176.479 | 0.2627 | 0.0283 | 0.0136 | 600 | 1 | 26.886 | 27.73 |
| 14 | 194.518 | 7.857 | 55.205 | 116.873 | 169.266 | 0.2576 | 0.0262 | 0.0187 | 600 | 1 | 26.891 | 27.69 |
| 15 | 102.831 | 5.938 | 42.793 | 74.334 | 221.166 | 0.3796 | 0.0427 | 0.0188 | 600 | 1 | 26.935 | 27.73 |
| 16 | 240.006 | 8.757 | 66.904 | 120.002 | 157.921 | 0.2368 | 0.0224 | 0.0085 | 600 | 1 | 26.932 | 27.22 |
| 17 | 96.359 | 11.108 | 80.001 | 82.106 | 195.304 | 0.1955 | 0.0427 | 0.0153 | 700 | 1 | 26.939 | 27.64 |
| 18 | 240.002 | 4.182 | 70.795 | 120.002 | 196.436 | 0.3056 | 0.0427 | 0.0147 | 700 | 1 | 26.946 | 27.58 |
| 19 | 117.977 | 10.745 | 80.005 | 100.606 | 240.008 | 0.1955 | 0.0428 | 0.0148 | 700 | 1 | 26.975 | 27.68 |
| 20 | 188.432 | 8.186 | 62.934 | 116.757 | 173.209 | 0.2524 | 0.0271 | 0.0099 | 600 | 1 | 26.991 | 27.72 |
| 21 | 206.442 | 3.672 | 78.948 | 120.005 | 229.604 | 0.3748 | 0.0253 | 0.0186 | 700 | 1 | 27.013 | 27.47 |
| 22 | 240.002 | 7.528 | 52.043 | 35.641 | 240.006 | 0.4204 | 0.0428 | 0.0171 | 600 | 1 | 27.021 | 27.74 |
| 23 | 240.004 | 8.304 | 80.007 | 80.034 | 186.258 | 0.4204 | 0.0269 | 0.0186 | 700 | 1 | 27.033 | 27.61 |
| 24 | 60.005 | 3.267 | 80.006 | 30.007 | 169.712 | 0.4204 | 0.0428 | 0.0186 | 600 | 1 | 27.043 | 27.24 |
| 25 | 208.025 | 7.637 | 77.561 | 81.402 | 240.006 | 0.2916 | 0.0428 | 0.0159 | 700 | 1 | 27.059 | 27.52 |
| 26 | 78.798 | 9.497 | 80.005 | 49.292 | 183.601 | 0.3967 | 0.0428 | 0.0186 | 700 | 1 | 27.065 | 27.49 |
| 27 | 60.003 | 11.213 | 77.911 | 30.002 | 124.978 | 0.1955 | 0.0428 | 0.0186 | 700 | 1 | 27.071 | 27.49 |
| 28 | 200.747 | 8.794 | 20.001 | 40.812 | 199.674 | 0.234 | 0.0258 | 0.0158 | 600 | 1 | 27.077 | 27.47 |
| 29 | 60.002 | 9.064 | 50.313 | 111.947 | 106.104 | 0.2516 | 0.0231 | 0.0107 | 600 | 1 | 27.095 | 27.62 |
| 30 | 133.225 | 11.908 | 80.003 | 30.002 | 117.232 | 0.2337 | 0.0411 | 0.0084 | 600 | 1 | 27.104 | 27.29 |
| 31 | 240.006 | 3.004 | 20.001 | 120.001 | 240.003 | 0.1955 | 0.0196 | 0.0186 | 600 | 1 | 27.107 | 27.29 |
| 32 | 240.002 | 3.008 | 20.007 | 120.006 | 240.008 | 0.1955 | 0.0196 | 0.0176 | 600 | 1 | 27.107 | 27.67 |
| 33 | 137.768 | 10.812 | 80.004 | 30.002 | 145.322 | 0.4204 | 0.0428 | 0.0186 | 700 | 1 | 27.145 | 27.69 |
| 34 | 234.038 | 3.001 | 78.272 | 120.002 | 122.099 | 0.3951 | 0.0428 | 0.0102 | 700 | 1 | 27.209 | 27.22 |
| 35 | 196.032 | 11.616 | 74.171 | 120.002 | 195.796 | 0.2454 | 0.0397 | 0.0186 | 700 | 1 | 27.265 | 27.47 |
| 36 | 240.006 | 4.738 | 20.004 | 120.008 | 240.008 | 0.4204 | 0.0428 | 0.0156 | 700 | 1 | 27.298 | 27.73 |
| 37 | 233.767 | 8.453 | 20.142 | 120.002 | 229.513 | 0.4081 | 0.0416 | 0.0113 | 600 | 1 | 27.306 | 27.43 |
| 38 | 64.384 | 8.756 | 46.344 | 120.005 | 88.563 | 0.4204 | 0.0428 | 0.0169 | 700 | 1 | 27.356 | 27.49 |
| 39 | 239.208 | 11.777 | 56.443 | 31.962 | 69.085 | 0.1955 | 0.0342 | 0.0119 | 800 | 1 | 27.408 | 27.49 |
| 40 | 240.001 | 3.688 | 20.001 | 120.008 | 169.415 | 0.3337 | 0.0196 | 0.0114 | 600 | 1 | 27.418 | 27.45 |

Table S26. Optimization schemes and PCR testing results from the seventh iteration (II)

| No. | Guanidine isothiocyanate (g) | Potassium chloride (g) | PEG 8000 (g) | Tween-20 (g) | Acetone (g) | Methanol (wt%) | EDTA (wt%) | Tris-HCl (wt%) | Particle diameter (nm) | Carboxyl | Predicted Ct | Tested Ct |
| --- | --- | --- | --- | --- | --- | --- | --- | --- | --- | --- | --- | --- |
| 41 | 234.908 | 10.646 | 63.304 | 51.239 | 106.092 | 0.2046 | 0.0355 | 0.0094 | 800 | 1 | 27.465 | 27.35 |
| 42 | 195.458 | 3.001 | 20.007 | 120.002 | 78.775 | 0.3232 | 0.0208 | 0.0143 | 600 | 1 | 27.513 | 27.58 |
| 43 | 240.006 | 12.009 | 80.005 | 120.006 | 60.007 | 0.3711 | 0.0422 | 0.0182 | 700 | 1 | 27.672 | 27.48 |
| 44 | 131.079 | 5.641 | 20.845 | 47.896 | 188.042 | 0.3732 | 0.0332 | 0.0173 | 900 | 2 | 27.877 | 27.24 |
| 45 | 211.611 | 7.048 | 43.709 | 113.399 | 190.909 | 0.2695 | 0.0324 | 0.0134 | 1000 | 2 | 27.934 | 27.53 |
| 46 | 131.975 | 3.421 | 78.425 | 50.956 | 76.309 | 0.3346 | 0.0282 | 0.0179 | 800 | 2 | 27.973 | 27.34 |
| 47 | 70.154 | 9.498 | 76.313 | 30.078 | 238.598 | 0.3344 | 0.0333 | 0.0084 | 600 | 2 | 27.053 | 27.84 |
| 48 | 203.378 | 4.651 | 66.782 | 83.716 | 140.253 | 0.2185 | 0.0299 | 0.0116 | 600 | 2 | 27.134 | 27.44 |


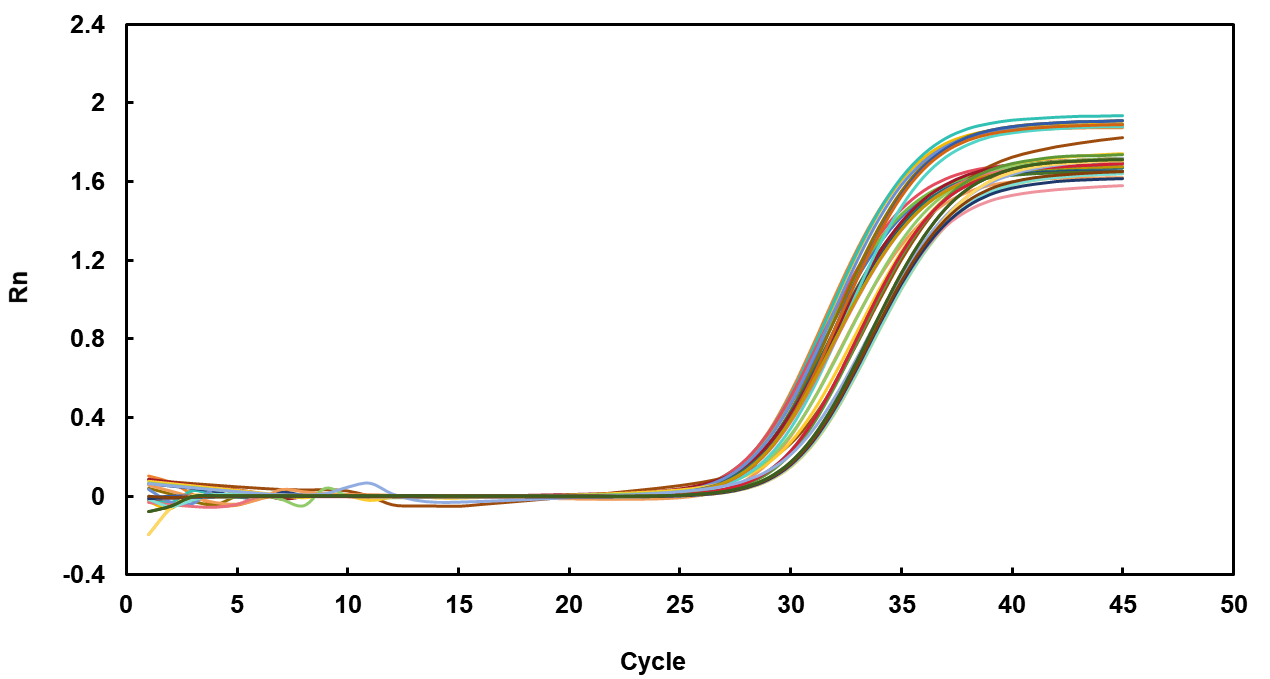


Figure S18. PCR curves from the seventh round of testing.

22.8. Eighth-round recommended schemes and autonomous PCR testing results

Table S27. Optimization schemes and PCR testing results from the eighth iteration (I)

| No. | Guanidine isothiocyanate (g) | Ammonium sulphate (g) | PEG 8000 (g) | Tween-20 (g) | Isopropanol (g) | Chloroform (wt%) | EDTA (wt%) | MOPS (wt%) | Particle diameter (nm) | Carboxyl | Predicted Ct | Tested Ct |
| --- | --- | --- | --- | --- | --- | --- | --- | --- | --- | --- | --- | --- |
| 1 | 169.799 | 10.498 | 30.402 | 65.195 | 92.802 | 0.3654 | 0.0291 | 0.0104 | 800 | 1 | 26.904 | 26.92 |
| 2 | 60.025 | 11.408 | 80.845 | 69.629 | 240.154 | 0.1955 | 0.0427 | 0.0125 | 900 | 1 | 27.511 | 26.97 |
| 3 | 213.006 | 7.045 | 25.724 | 63.373 | 180.391 | 0.3453 | 0.0329 | 0.0118 | 800 | 1 | 27.327 | 27.58 |
| 4 | 60.254 | 7.374 | 24.147 | 57.206 | 195.452 | 0.4204 | 0.0246 | 0.0188 | 1000 | 1 | 27.527 | 27.39 |
| 5 | 60.125 | 5.238 | 46.543 | 30.125 | 240.154 | 0.1955 | 0.0374 | 0.0087 | 700 | 1 | 26.655 | 27.45 |
| 6 | 60.126 | 3.406 | 78.829 | 62.532 | 240.136 | 0.1955 | 0.0403 | 0.0139 | 700 | 1 | 26.694 | 27.47 |
| 7 | 137.678 | 5.196 | 67.096 | 120.456 | 240.154 | 0.2288 | 0.0271 | 0.0146 | 700 | 1 | 26.718 | 27.17 |
| 8 | 174.325 | 8.822 | 49.428 | 69.742 | 221.031 | 0.2491 | 0.0417 | 0.0157 | 600 | 1 | 26.735 | 27.25 |
| 9 | 87.756 | 3.500 | 21.388 | 34.548 | 225.781 | 0.3346 | 0.0292 | 0.0098 | 700 | 1 | 26.773 | 27.23 |
| 10 | 165.197 | 8.405 | 69.447 | 120.568 | 179.537 | 0.2482 | 0.0291 | 0.0104 | 600 | 1 | 26.844 | 27.46 |
| 11 | 60.136 | 12.125 | 80.154 | 120.485 | 240.154 | 0.1955 | 0.0286 | 0.0084 | 600 | 1 | 26.874 | 26.98 |
| 12 | 240.456 | 3.364 | 45.508 | 37.254 | 199.254 | 0.3108 | 0.0427 | 0.0167 | 600 | 1 | 26.877 | 28.58 |
| 13 | 124.286 | 7.044 | 71.128 | 89.204 | 176.479 | 0.2627 | 0.0283 | 0.0136 | 600 | 1 | 26.886 | 27.28 |
| 14 | 194.517 | 7.857 | 55.205 | 116.872 | 169.266 | 0.2576 | 0.0262 | 0.0187 | 600 | 1 | 26.891 | 27.05 |
| 15 | 102.830 | 5.938 | 42.793 | 74.334 | 221.166 | 0.3796 | 0.0427 | 0.0185 | 600 | 1 | 26.935 | 27.03 |
| 16 | 240.154 | 8.756 | 66.903 | 120.456 | 157.921 | 0.2368 | 0.0224 | 0.0085 | 600 | 1 | 26.932 | 27.28 |
| 17 | 96.359 | 11.125 | 80.154 | 82.106 | 195.304 | 0.1955 | 0.0427 | 0.0153 | 700 | 1 | 26.935 | 27.59 |
| 18 | 240.124 | 4.179 | 70.790 | 120.125 | 196.436 | 0.3056 | 0.0427 | 0.0147 | 700 | 1 | 26.946 | 27.16 |
| 19 | 117.977 | 10.745 | 80.546 | 100.606 | 240.486 | 0.1955 | 0.0428 | 0.0148 | 700 | 1 | 26.975 | 27.09 |
| 20 | 188.432 | 8.186 | 62.934 | 116.756 | 173.208 | 0.2524 | 0.0271 | 0.0099 | 600 | 1 | 26.991 | 27.14 |
| 21 | 206.442 | 3.672 | 78.948 | 120.364 | 229.604 | 0.3748 | 0.0253 | 0.0185 | 700 | 1 | 27.013 | 27.25 |
| 22 | 240.125 | 7.528 | 52.042 | 35.641 | 240.596 | 0.4204 | 0.0428 | 0.0171 | 600 | 1 | 27.021 | 26.91 |
| 23 | 240.256 | 8.304 | 80.459 | 80.034 | 186.255 | 0.4204 | 0.0269 | 0.0188 | 700 | 1 | 27.033 | 27.55 |
| 24 | 60.456 | 3.267 | 80.865 | 30.136 | 169.711 | 0.4204 | 0.0425 | 0.0185 | 600 | 1 | 27.043 | 27.49 |
| 25 | 208.024 | 7.637 | 77.568 | 81.401 | 240.364 | 0.2916 | 0.0422 | 0.0159 | 700 | 1 | 27.059 | 26.96 |
| 26 | 78.798 | 9.497 | 80.158 | 49.292 | 183.601 | 0.3954 | 0.0423 | 0.0186 | 700 | 1 | 27.065 | 27.14 |
| 27 | 60.468 | 11.212 | 77.911 | 30.456 | 124.978 | 0.1955 | 0.0422 | 0.0186 | 700 | 1 | 27.071 | 27.57 |
| 28 | 200.746 | 8.794 | 20.548 | 40.814 | 199.674 | 0.2345 | 0.0258 | 0.0155 | 600 | 1 | 27.077 | 27.48 |
| 29 | 60.458 | 9.064 | 50.312 | 111.946 | 106.103 | 0.2516 | 0.0231 | 0.0107 | 600 | 1 | 27.095 | 27.48 |
| 30 | 133.219 | 11.907 | 80.594 | 30.364 | 117.232 | 0.2337 | 0.0411 | 0.0084 | 600 | 1 | 27.104 | 27.28 |
| 31 | 240.435 | 3.459 | 20.368 | 120.125 | 240.468 | 0.1955 | 0.0196 | 0.0185 | 600 | 1 | 27.107 | 27.33 |
| 32 | 240.965 | 3.154 | 20.264 | 120.157 | 240.486 | 0.1955 | 0.0196 | 0.0176 | 600 | 1 | 27.107 | 27.24 |
| 33 | 137.768 | 10.811 | 80.256 | 30.456 | 145.321 | 0.4204 | 0.0422 | 0.0185 | 700 | 1 | 27.145 | 27.58 |
| 34 | 234.038 | 3.136 | 78.272 | 120.458 | 122.098 | 0.3951 | 0.0422 | 0.0102 | 700 | 1 | 27.209 | 27.09 |
| 35 | 196.032 | 11.616 | 74.169 | 120.489 | 195.796 | 0.2454 | 0.0395 | 0.0188 | 700 | 1 | 27.265 | 27.41 |
| 36 | 240.159 | 4.738 | 20.264 | 120.456 | 240.546 | 0.4204 | 0.0422 | 0.0156 | 700 | 1 | 27.298 | 27.34 |
| 37 | 233.767 | 8.453 | 20.142 | 120.138 | 229.512 | 0.4081 | 0.0416 | 0.0113 | 600 | 1 | 27.306 | 27.41 |
| 38 | 64.384 | 8.756 | 46.343 | 120.369 | 88.563 | 0.4204 | 0.0422 | 0.0169 | 700 | 1 | 27.356 | 27.14 |
| 39 | 239.208 | 11.776 | 56.443 | 31.961 | 69.080 | 0.1955 | 0.0342 | 0.0119 | 700 | 1 | 27.408 | 27.32 |
| 40 | 60.563 | 3.922 | 51.235 | 30.436 | 42.353 | 0.3337 | 0.0196 | 0.0157 | 600 | 1 | 27.418 | 27.48 |

Table S28. Optimization schemes and PCR testing results from the eighth iteration (II)

| No. | Guanidine isothiocyanate (g) | Sodium acetate (g) | PEG 8000 (g) | Tween-20 (g) | Ethanol (g) | Chloroform (wt%) | EGTA (wt%) | HEPES (wt%) | Particle diameter (nm) | Carboxyl | Predicted Ct | Tested Ct |
| --- | --- | --- | --- | --- | --- | --- | --- | --- | --- | --- | --- | --- |
| 41 | 234.908 | 10.640 | 63.303 | 51.238 | 106.092 | 0.2046 | 0.0355 | 0.0094 | 800 | 1 | 27.465 | 27.56 |
| 42 | 195.457 | 3.235 | 20.465 | 120.268 | 78.770 | 0.3232 | 0.0208 | 0.0143 | 600 | 1 | 27.513 | 27.52 |
| 43 | 240.236 | 12.125 | 80.459 | 120.456 | 60.264 | 0.3711 | 0.0424 | 0.0185 | 700 | 1 | 27.672 | 27.52 |
| 44 | 131.078 | 5.641 | 20.844 | 47.896 | 188.041 | 0.3732 | 0.0332 | 0.0173 | 800 | 2 | 26.877 | 27.48 |
| 45 | 211.611 | 7.047 | 43.709 | 113.399 | 190.908 | 0.269 | 0.0324 | 0.0134 | 1000 | 2 | 26.934 | 26.92 |
| 46 | 131.974 | 3.424 | 78.425 | 50.949 | 76.309 | 0.3346 | 0.0282 | 0.0179 | 800 | 2 | 26.973 | 27.07 |
| 47 | 70.154 | 9.498 | 76.313 | 30.077 | 238.598 | 0.3344 | 0.0333 | 0.0084 | 600 | 2 | 27.053 | 27.09 |
| 48 | 203.377 | 4.650 | 66.781 | 83.716 | 140.254 | 0.2184 | 0.0299 | 0.0116 | 700 | 2 | 27.134 | 26.96 |


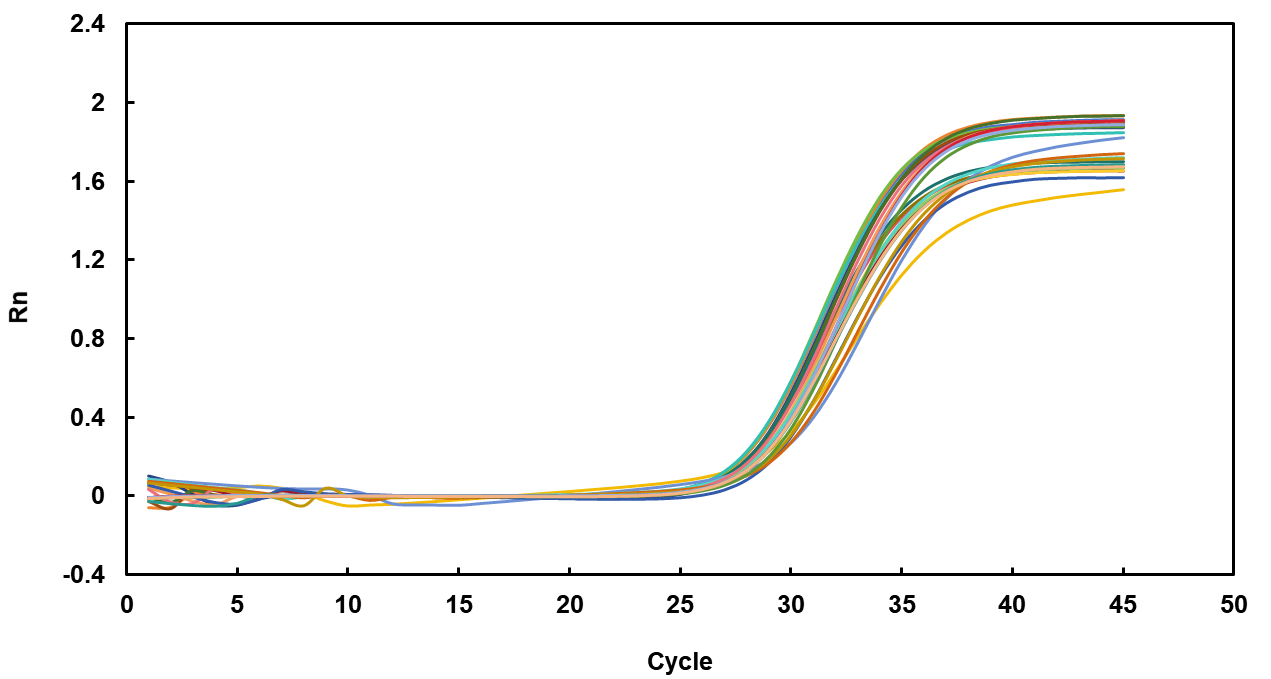


Figure S19. PCR curves from the eighth round of testing.

22.9. Ninth-round recommended schemes and autonomous PCR testing results

Table S29. Optimization schemes and PCR testing results from the ninth iteration (I)

| No. | Guanidine isothiocyanate (g) | Sodium acetate (g) | Ficoll 400 (g) | Tween-20 (g) | Ethanol (g) | Methanol (wt%) | EGTA (wt%) | HEPES (wt%) | Particle diameter (nm) | Carboxyl | Predicted Ct | Tested Ct |
| --- | --- | --- | --- | --- | --- | --- | --- | --- | --- | --- | --- | --- |
| 1 | 169.799 | 10.498 | 30.402 | 65.195 | 92.802 | 0.3654 | 0.0291 | 0.0104 | 800 | 1 | 26.904 | 26.53 |
| 2 | 160.252 | 11.408 | 80.267 | 69.629 | 240.124 | 0.1955 | 0.0426 | 0.0125 | 900 | 1 | 27.511 | 26.59 |
| 3 | 213.006 | 7.045 | 25.724 | 63.373 | 180.391 | 0.3453 | 0.0329 | 0.0112 | 800 | 1 | 27.327 | 26.61 |
| 4 | 160.598 | 7.374 | 24.147 | 57.206 | 195.452 | 0.4204 | 0.0246 | 0.0185 | 1000 | 1 | 26.527 | 26.22 |
| 5 | 150.985 | 5.238 | 46.543 | 30.396 | 240.486 | 0.1955 | 0.0374 | 0.0087 | 700 | 1 | 26.655 | 26.54 |
| 6 | 156.234 | 13.406 | 78.829 | 62.532 | 240.423 | 0.1955 | 0.0403 | 0.0139 | 700 | 1 | 26.694 | 26.58 |
| 7 | 137.678 | 5.196 | 67.096 | 120.759 | 240.486 | 0.2288 | 0.0271 | 0.0146 | 700 | 1 | 31.718 | 26.77 |
| 8 | 174.324 | 8.822 | 49.428 | 69.742 | 221.031 | 0.2491 | 0.0417 | 0.0154 | 600 | 1 | 27.735 | 26.56 |
| 9 | 87.756 | 3.502 | 21.388 | 34.548 | 225.781 | 0.3346 | 0.0292 | 0.0092 | 700 | 1 | 26.773 | 26.53 |
| 10 | 165.197 | 8.405 | 69.447 | 120.654 | 179.537 | 0.2482 | 0.0291 | 0.0104 | 600 | 1 | 26.844 | 26.31 |
| 11 | 182.265 | 12.212 | 80.268 | 120.268 | 240.265 | 0.1955 | 0.0282 | 0.0084 | 600 | 1 | 26.874 | 26.76 |
| 12 | 240.236 | 13.256 | 45.508 | 37.254 | 199.256 | 0.3108 | 0.0425 | 0.0167 | 600 | 1 | 27.877 | 26.48 |
| 13 | 124.286 | 7.044 | 71.128 | 89.204 | 176.479 | 0.2627 | 0.0283 | 0.0136 | 600 | 1 | 25.886 | 26.62 |
| 14 | 194.517 | 7.857 | 55.205 | 116.872 | 169.266 | 0.2576 | 0.0262 | 0.0123 | 600 | 1 | 25.891 | 26.54 |
| 15 | 102.831 | 5.938 | 42.793 | 74.334 | 221.166 | 0.3796 | 0.0426 | 0.0185 | 600 | 1 | 26.932 | 26.64 |
| 16 | 240.186 | 8.756 | 66.903 | 120.236 | 157.921 | 0.2368 | 0.0224 | 0.0085 | 600 | 1 | 25.932 | 26.55 |
| 17 | 96.359 | 11.136 | 80.369 | 82.106 | 195.304 | 0.1955 | 0.0425 | 0.0153 | 700 | 1 | 25.939 | 26.52 |
| 18 | 240.265 | 4.179 | 70.794 | 120.267 | 196.436 | 0.3056 | 0.0425 | 0.0147 | 700 | 1 | 25.946 | 26.24 |
| 19 | 117.977 | 10.745 | 80.265 | 100.606 | 240.548 | 0.1955 | 0.0426 | 0.0148 | 700 | 1 | 25.975 | 26.75 |
| 20 | 188.432 | 8.186 | 62.934 | 116.756 | 173.208 | 0.2524 | 0.0271 | 0.0099 | 600 | 1 | 25.991 | 26.68 |
| 21 | 206.442 | 3.672 | 78.948 | 120.158 | 229.604 | 0.3748 | 0.0253 | 0.0182 | 700 | 1 | 26.013 | 26.78 |
| 22 | 240.215 | 7.528 | 52.042 | 35.641 | 240.265 | 0.4204 | 0.0428 | 0.0171 | 600 | 1 | 27.021 | 26.61 |
| 23 | 240.867 | 8.304 | 80.369 | 80.034 | 186.255 | 0.4204 | 0.0269 | 0.0188 | 700 | 1 | 26.033 | 26.52 |
| 24 | 160.367 | 3.267 | 80.234 | 30.369 | 169.711 | 0.4204 | 0.0427 | 0.0185 | 600 | 1 | 26.043 | 26.13 |
| 25 | 208.024 | 7.637 | 77.567 | 81.401 | 240.285 | 0.2916 | 0.0425 | 0.0159 | 700 | 1 | 27.059 | 26.11 |
| 26 | 78.798 | 9.497 | 80.364 | 49.292 | 183.601 | 0.3943 | 0.0422 | 0.0182 | 700 | 1 | 26.065 | 26.53 |
| 27 | 160.596 | 11.212 | 77.911 | 30.268 | 124.978 | 0.1955 | 0.0423 | 0.0182 | 700 | 1 | 26.071 | 26.53 |
| 28 | 200.746 | 8.794 | 20.268 | 40.812 | 199.674 | 0.2344 | 0.0258 | 0.0156 | 600 | 1 | 26.077 | 26.82 |
| 29 | 160.354 | 9.064 | 50.313 | 111.946 | 106.103 | 0.2516 | 0.0231 | 0.0107 | 600 | 1 | 26.095 | 26.88 |
| 30 | 133.219 | 11.907 | 80.235 | 30.268 | 117.232 | 0.2337 | 0.0411 | 0.0084 | 600 | 1 | 26.104 | 26.02 |
| 31 | 240.235 | 3.267 | 20.568 | 120.453 | 240.286 | 0.1955 | 0.0196 | 0.0182 | 600 | 1 | 26.107 | 26.72 |
| 32 | 240.364 | 3.598 | 25.436 | 120.268 | 240.265 | 0.1955 | 0.0196 | 0.0176 | 600 | 1 | 26.107 | 26.75 |
| 33 | 137.768 | 10.811 | 80.268 | 30.358 | 145.321 | 0.4204 | 0.0423 | 0.0186 | 700 | 1 | 26.145 | 26.05 |
| 34 | 234.038 | 3.356 | 78.272 | 120.263 | 122.098 | 0.3951 | 0.0423 | 0.0102 | 700 | 1 | 26.209 | 26.65 |
| 35 | 196.034 | 11.616 | 74.169 | 120.258 | 195.796 | 0.2454 | 0.0392 | 0.0185 | 700 | 1 | 26.265 | 26.74 |
| 36 | 240.365 | 4.738 | 20.236 | 120.165 | 240.158 | 0.4204 | 0.0422 | 0.0156 | 700 | 1 | 26.298 | 26.69 |
| 37 | 233.767 | 8.453 | 20.142 | 120.145 | 229.512 | 0.4081 | 0.0416 | 0.0113 | 600 | 1 | 26.306 | 26.62 |
| 38 | 64.384 | 8.756 | 46.343 | 120.125 | 88.563 | 0.4204 | 0.0423 | 0.0169 | 700 | 1 | 26.356 | 26.14 |
| 39 | 239.208 | 11.776 | 56.443 | 31.961 | 69.084 | 0.1955 | 0.0342 | 0.0119 | 700 | 1 | 26.408 | 26.67 |
| 40 | 240.356 | 3.688 | 20.564 | 120.125 | 169.414 | 0.3337 | 0.0196 | 0.0157 | 600 | 1 | 26.418 | 26.67 |

Table S30. Optimization schemes and PCR testing results from the ninth iteration (II)

| No. | Guanidine isothiocyanate (g) | Sodium acetate (g) | PEG 2000 (g) | Tween-20 (g) | Acetone (g) | Methanol (wt%) | EGTA (wt%) | MOPS (wt%) | Particle diameter (nm) | Carboxyl | Predicted Ct | Tested Ct |
| --- | --- | --- | --- | --- | --- | --- | --- | --- | --- | --- | --- | --- |
| 41 | 234.908 | 10.640 | 63.303 | 91.238 | 106.092 | 0.2046 | 0.0355 | 0.0094 | 800 | 1 | 26.465 | 26.65 |
| 42 | 195.457 | 8.362 | 60.158 | 120.562 | 178.770 | 0.3232 | 0.0208 | 0.0143 | 600 | 1 | 26.513 | 26.88 |
| 43 | 240.026 | 12.285 | 68.356 | 120.235 | 160.528 | 0.3711 | 0.0424 | 0.0180 | 700 | 1 | 26.672 | 26.62 |
| 44 | 181.078 | 7.641 | 60.845 | 87.896 | 188.041 | 0.3732 | 0.0332 | 0.0173 | 900 | 2 | 26.877 | 26.82 |
| 45 | 211.611 | 7.047 | 43.709 | 113.399 | 190.908 | 0.2695 | 0.0324 | 0.0134 | 1000 | 2 | 26.934 | 26.55 |
| 46 | 181.974 | 8.423 | 78.425 | 50.949 | 176.309 | 0.3346 | 0.0282 | 0.0179 | 800 | 2 | 26.973 | 26.86 |
| 47 | 170.154 | 9.498 | 76.313 | 90.072 | 238.598 | 0.3344 | 0.0333 | 0.0084 | 600 | 2 | 26.053 | 26.54 |
| 48 | 203.377 | 8.651 | 66.782 | 83.716 | 140.252 | 0.2180 | 0.0299 | 0.0116 | 600 | 2 | 26.134 | 26.64 |


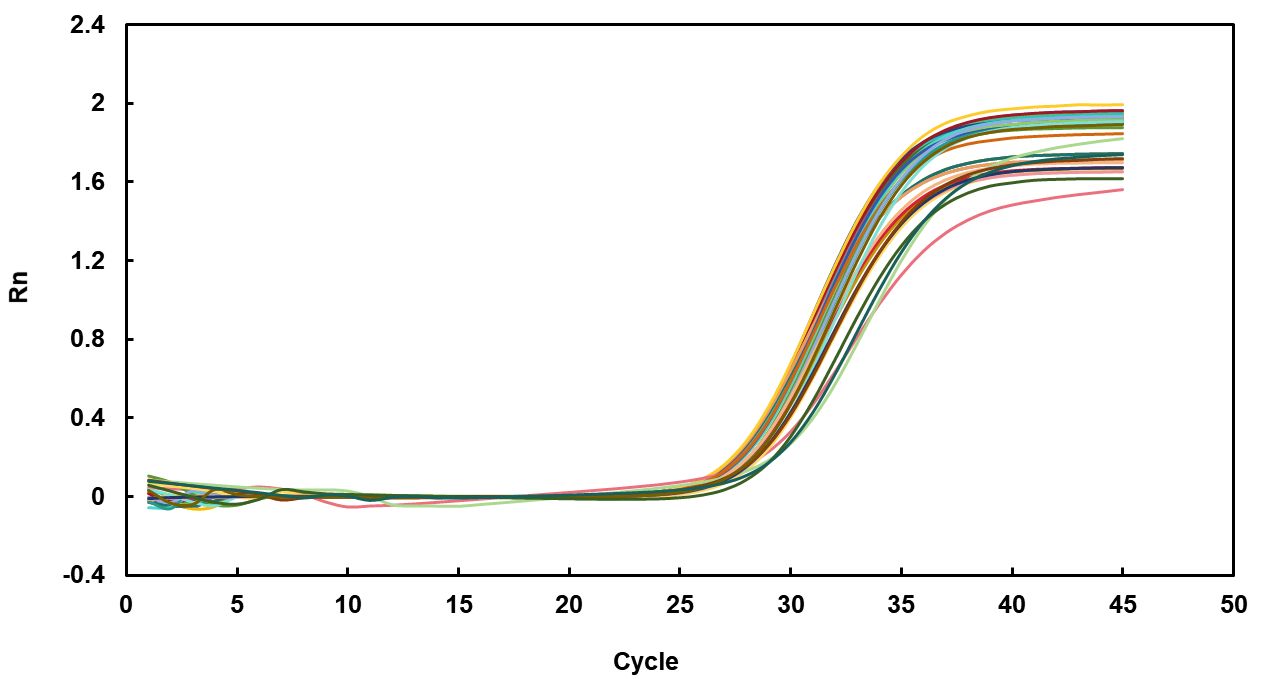


Figure S20. PCR curves from the ninth round of testing.

21. Supplementary Materials Figure S21: Quantifying coverage and sparsity of industrial vs literature datasets.

To complement the qualitative interpretation of Figure 3d, we quantify the distribution differences between industrial and literature datasets in the ten-dimensional feature space (particle size, surfactant, salting out reagent, buffer, functional group, chelation reagent, molecular crowding reagent, precipitation reagent, chaotropic agent, and solvent) in Figure S21. After z-score standardization, we compute (i) parameter-space coverage using the convex-hull area in the PC1-PC2 plane and the generalized variance (log-determinant of the 10D covariance matrix), (ii) sample density using the median 5-nearest-neighbor distance, and (iii) Pearson correlation coefficients between each feature and Ct. Consistent with Figure 3d, industrial datasets occupy a compact manufacturable manifold with higher local density, whereas literature datasets spread over a broader but sparser region. These quantitative descriptors explain why optimization trajectories initialized from industrial datasets converge faster and more stably toward low Ct solutions, while literature based trajectories more frequently explore parameter combinations that are scientifically plausible but less robust for pilot-scale performance.


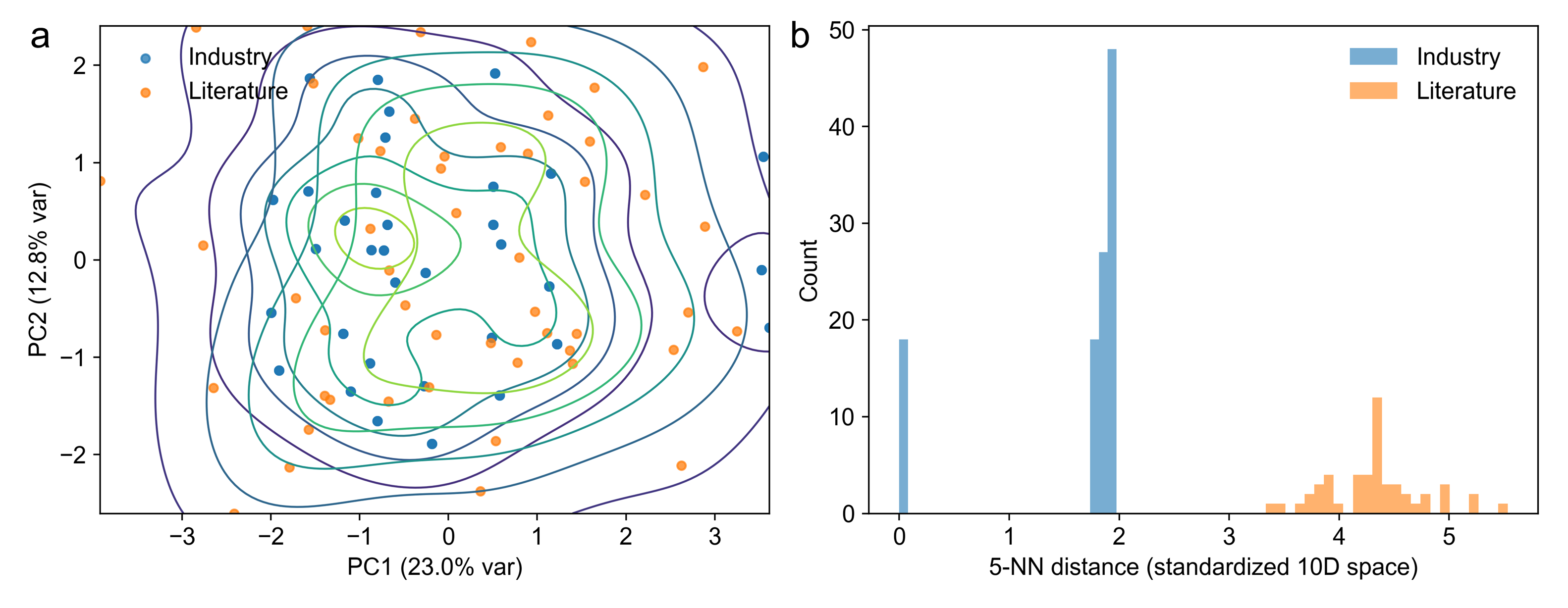


Figure S21. PCA distribution and density of industrial versus literature datasets. **a**, PC1–PC2 projection of the standardized ten-dimensional feature space, colored by data source, with density contours highlighting a compact “manufacturable” region for industrial datasets and a broader, sparser distribution for literature datasets. **b**, Sparsity comparison using the 5-nearest-neighbor distance distribution in standardized feature space, showing higher local density for industrial datasets.

22. Supplementary Materials M2: The detailed information of commercial products A (DAAN), NAs extraction and purification kit instruction manual

Product Name: MNPs Based NAs Isolation Kit

Packaging Specifications:
Large package: 500 reactions/kit
Medium package: 200 reactions/kit
Small package: 32 reactions/kit

Intended Use: This kit is suitable for extracting and purifying NAs (DNA/RNA) from a variety of samples. The extracted NAs can be directly used for subsequent clinical molecular detection (e.g., PCR or real-time PCR).

Storage Conditions and Expiry: Store at room temperature (15–25°C) in a dry, ventilated area, avoiding direct sunlight.
 Shelf life: 12 months.

Main Components

| Component Name | 500 rxns | 200 rxns | 32 rxns |
| --- | --- | --- | --- |
| MNPs | 30 mL | 12 mL | 2 mL |
| Lysis/binding buffer (contains guanidine salts) | 500 mL | 200 mL | 32 mL |
| Washing solution 1 (contains ethanol) | 500 mL | 200 mL | 32 mL |
| Washing solution 2 (contains ethanol) | 500 mL | 200 mL | 32 mL |
| Elution buffer | 30 mL | 12 mL | 2 mL |

Compatible Instruments

Smart32 Fully Automated NAs Extractor
Smart32 Plus Fully Automated NAs Extractor
Stream SP96 Fully Automated NAs Extractor
Ballet X3 Automated NAs Extractor
DA3200, DA3300, DA3500 Series Extractors
Kingfisher96 and other compatible systems

Sample Requirements

1. Applicable Sample Types

Whole blood, serum, plasma, throat swabs, nasal swabs, saliva, sputum, Broncho alveolar lavage fluid, etc.

2. Sample Handling Notes

- Fresh samples are recommended. If short-term storage is required, keep samples at 2-8°C for up to 24 hours.
- For long-term storage, keep at -20°C or -80°C. Avoid repeated freeze-thaw cycles.
- Throat swab and nasal swab samples should be collected into viral transport media (VTM) or normal saline.
- For whole blood: collect in anticoagulant tubes (EDTA or sodium citrate). Use within 24 hours.
- If the sample is viscous, dilute with normal saline before use.
- For samples with potential biosafety risks, inactivate at 56°C for 30 minutes before extraction.
- Samples must be well mixed before use. If precipitates are present, vortex and briefly centrifuge to collect at the bottom of the tube. Use 200–600 μL as sample input.
- Recommended: use 2 mL screw-cap microcentrifuge tubes. Do not use tubes with poor sealing, to avoid leakage during extraction.

Sample Pretreatment

1. Pretreatment of Swab Samples

After collecting swabs, immerse the swab head in 0.5-1.5 mL of transport medium (normal saline or VTM).

Vortex for 30 seconds, discard the swab stick, and use 0.5 mL for extraction.

2. Pretreatment of Sputum Samples

- Mix sputum with an equal volume of 0.1-0.5 M NaOH or digestive solution, vortex and incubate at room temperature for 30 minutes, then proceed with extraction.
- If high viscosity remains, centrifuge and take the supernatant.

Instructions for Use

1. Manual Extraction

1.1 Small Kit (20 Reactions/Kit)

- Add 3.8 mL of Lysis/Binding Buffer into each sample tube, then add 200 μL sample and 10 μL MNPs. Vortex to mix and incubate.
- Add 800 μL Wash Buffer 1, wash, discard supernatant.
- Add 800 μL Wash Buffer 2, wash again, discard supernatant.
- Add 100 μL Elution Buffer to elute NAs.

1.2 Large Kit (96 Reactions/Kit)

- Add 17.5 mL Lysis/Binding Buffer to each sample well, then add 1 mL sample and 50 μL MNPs. Mix well and proceed with extraction.
- Wash with 3 mL of each wash buffer, then elute with 100 μL Elution Buffer.

2. Automated Extraction

For **Smart32** Automated NAs Extractor:

- Use 2x 96-deep-well plates. Each 96-well plate can process up to 16 samples per run.
- Reagent loading instructions:
  - Plate 1, rows 1–3: 600 μL Wash Buffer 1
  - Plate 1, rows 4–6: 600 μL Wash Buffer 2
  - Plate 2, row 1: 400 μL Lysis/Binding Buffer + sample + MNPs
  - Plate 2, row 2: 100 μL Elution Buffer
- Use matching magnetic tip combs and heating blocks.
- Follow instrument software prompts for sample ID and program selection.

Program Settings (Smart32)

| Step | Position | Name | Waiting Time | Mixing Time | Magnetic Strength | Mixing Speed | Tip Row | Temp Zone | Temp (°C) |
| --- | --- | --- | --- | --- | --- | --- | --- | --- | --- |
| 1 | 1 | Lysis | 0 min | 5 min | 3 | Fast | Row 1 | T1 | 70 |
| 2 | 2 | Magnetic Separation | 0 min | 3 min | 3 | — | Row 1 | — | — |
| 3 | 3 | Wash 1 | 0 min | 2 min | 2 | Medium | Row 2 | T2 | 50 |
| 4 | 4 | Wash 2 | 0 min | 2 min | 2 | Medium | Row 3 | T3 | 50 |
| 5 | 5 | Elution | 0 min | 5 min | 2 | Medium | Row 4 | T4 |  |

**Deep-Well Plate Layout**

Use a 96-deep-well plate. Load the reagents as required, centrifuge briefly, and ensure MNPs are evenly distributed. Use 96-deep-well magnetic tip combs. If using a shaker plate module, set shaking speed to 500 rpm.

- Load 600 μL Wash Buffer 1 into each of the wells in row 1.
- Load 600 μL Wash Buffer 2 into each of the wells in row 2.
- Into row 3, add:
  - 400 μL lysis/binding buffer
  - 200 μL sample
  - 20 μL MNPs
- Row 4: 100 μL Elution Buffer

Program Table (20 reactions/box)

| Step | Position | Step Name | Wait Time (min) | Mixing Time (min) | Magnetic Strength | Mixing Speed | Tip Row | Temp Zone | Temp (°C) |
| --- | --- | --- | --- | --- | --- | --- | --- | --- | --- |
| 1 | 1 | Lysis | 0 | 5 | 3 | Medium | Row 1 | T1 | 70 |
| 2 | 2 | Magnetic Sep. | 0 | 3 | 3 | — | Row 1 | — | — |
| 3 | 3 | Wash 1 | 0 | 1.5 | 2 | Fast | Row 2 | T2 | 70 |
| 4 | 4 | Wash 2 | 0 | 1.5 | 2 | Medium | Row 3 | T3 | 70 |
| 5 | 5 | Magnetic Sep. | 0 | 1 | 3 | — | Row 3 | — | — |
| 6 | 6 | Elution | 0 | 5 | 2 | Medium | Row 4 | T4 | 70 |

After the run is complete, remove the 12-tip comb and discard it. Transfer the eluate to the final storage tube. For long-term storage, store at -20°C to -80°C.

Smart 32 Plus Fully Automated Extraction System

**Large Kit: 96 Reactions/Box**

**Plate Layout:** Same as Smart 32 standard, using 2 × 96 deep-well plates and 96-tip magnetic combs:

- Row 1: 600 μL Wash Buffer 1
- Row 2: 600 μL Wash Buffer 2
- Row 3: 400 μL Lysis/Binding Buffer + 200 μL Sample + 20 μL MNPs
- Row 4: 100 μL Elution Buffer

Eluate can be used immediately or stored at -20°C to -80°C.

IV. Manual Extraction: 32 Reactions/Box

**Deep-Well Plate Layout:** Same as above. Load reagents into each well, vortex briefly, and centrifuge if needed to evenly distribute MNPs.

- Add 400 μL Lysis/Binding Buffer, 200 μL sample, and 20 μL MNPs into each well.
- Add 600 μL of Wash Buffer 1 and Wash Buffer 2 into the respective wells.
- Add 100 μL Elution Buffer into elution wells.

Smart 32 Plus Program Table (for 96 Reactions/Box)

| Step | Action | Position | Tip Row | Action Parameters |
| --- | --- | --- | --- | --- |
| 1 | Lysis | Heating: 70°C | Magnetic mixing | On, Speed: Fast, 480 s |
| 2 | — | Tip Row: 1 | Magnetic mixing | Medium, Speed: 750 rpm |
| 3 | Wash 1 | Tip Row: 2 | Magnetic mixing | Medium, Speed: 600 rpm |
| 4 | Wash 2 | Tip Row: 3 | Magnetic mixing: Medium, Speed: 600 rpm | Volume (μL): 600, Repeats: 1 |
| 5 | — | Elution Heating | Heating: 70°C, Magnetic mixing: On | Heating Mode: On, Time (s): 0 |
| 6 | Mixing | — | Tip Row 6 | Time (s): 120 |
| 7 | Magnetic Sep. | Row 6 | Tip Row 4 | Volume (μL): 100, Repeats: 2 |
| 8 | Heating | — | Tip Row 3 | Time (s): 0 |

After program completion, collect the eluate from row 12. If immediate use is not possible, store the eluate at -20°C to -80°C.

II. Manual Extraction (20 Reactions/Box)

**Deep-Well Plate Layout**

Use a 96-deep-well plate. Load reagents according to layout, vortex briefly, and centrifuge to evenly distribute MNPs. Use 96-tip magnetic rods and plates; if using a shaker plate module, set to 500 rpm.

- Row A–H, Column 1: Add 400 μL Lysis/Binding Buffer + 200 μL Sample + 20 μL MNPs
- Row 2: Add 600 μL Wash Buffer 1
- Row 3: Add 600 μL Wash Buffer 2
- Row 4: Add 100 μL Elution Buffer

Program Table (Manual Extraction, 20 Reactions/Box)

| Step | Action | Tip Row | Operation Parameters |
| --- | --- | --- | --- |
| 1 | Magnetic Sep. | Row 2 | Mixing Speed: Medium, Volume: 600 μL, Repeats: 1 |
| 2 | Heating | Row 2 | Temp Mode: Heating, 70°C, Heating Mode: On, Time: 0 s |
| 3 | Lysis | Row 1 | Magnetic Mode: Medium, Mixing Speed: Fast, Time: 480 s |
| 4 | Wash 1 | Row 2 | Magnetic Mode: Medium, Volume: 750 μL, Repeats: 1 |
| 5 | Wash 2 | Row 6 | Heating Mode: 70°C, Mixing Speed: Fast, Time: 120 s |
| 6 | Magnetic Sep. | Row 6 | Magnetic Mode: Medium, Volume: 100 μL, Repeats: 2 |
| 7 | Elution | Row 4 | Heating Mode: Off, Time: 0 s, Magnetic Mode: Medium, Mixing Speed: Moderate |

After completion, collect eluate from row 12. If not used immediately, store at -20°C to -80°C.

IV. Manual Extraction: 32 Reactions/Box

**1. Deep-Well Plate Layout**

Same as above. Load reagents into 96-deep-well plates, vortex and centrifuge if needed, and ensure even bead dispersion. Use 96-tip magnetic rods; shaking plate speed: 500 rpm.

- Add 400 μL Lysis/Binding Buffer + 200 μL sample + 20 μL MNPs to row 1
- Row 2: Add 600 μL Wash Buffer 1
- Row 3: Add 600 μL Wash Buffer 2
- Row 4: Add 100 μL Elution Buffer

2. Stream SP96 Fully Automated NAs Extraction System

1. Turn on the power switch of the Stream SP96 instrument.
2. Launch the operating software of the Stream SP96 automated NAs extractor.
3. Place the 96-deep-well plate with samples into the instrument. Attach magnetic rods, tip combs, and heating blocks in place.
4. Confirm proper placement of consumables, then click “Start.” In the protocol selection window, select **"Protocol"**, enter the experiment number, and click “OK.”
5. The instrument will automatically execute the extraction procedure, including steps such as **sample lysis, NAs binding, washing, and elution**.
6. After the procedure, collect the eluate. For immediate use, proceed to detection. If storing, transfer eluate to sterile tubes and store at -20°C to -80°C.
7. For residual liquid in the plate, handle as biohazard waste. Dispose according to your institution’s biosafety guidelines.

Product Limitations

This product is intended only for the purposes stated in this manual. If used for other applications or outside the stated scope, the manufacturer assumes no responsibility for performance or safety.

Product Performance Indicators

1. This kit is compatible with a variety of sample types and fully automated extraction platforms.
2. It enables rapid, efficient extraction of high-purity NAs.
3. The MNPs recovery rate is ≥90%; NAs recovery rate is ≥85%; reproducibility (CV) is ≤5%.

Precautions

1. Please read the entire manual carefully before use.
2. This kit should be operated by trained laboratory personnel.
3. Avoid use in environments with bacterial or NAs contamination.
4. Do not reuse any disposable components.
5. Ensure pipette tips are replaced between samples to avoid cross-contamination.
6. Dispose of waste according to biosafety waste handling regulations.
7. Store reagents at 2-8°C for short term (≤24 h), or -20 to -80°C for long term. Avoid repeated freeze-thaw.
8. MNPs may settle over time. Before use, resuspend by gentle vertexing or inversion for 20-30 seconds.

23. Supplementary Materials M3: The detailed information of commercial products B (Thermo Fisher), NAs extraction and purification reagent manual

Product Name: General-purpose NAs extraction or purification reagent

Packaging Specification: 100 tests/kit

Intended Use: Used for the extraction and purification of NAs from human throat swabs, serum, plasma, and other clinical samples outside the body.

Target NAs: Total NAs (DNA and RNA)

Product Description: This product uses MNPs separation technology, enabling fast and efficient extraction of total NAs from a variety of sample types. The extracted NAs are of high purity and quality, suitable for downstream applications such as PCR amplification, reverse transcription PCR, and fluorescence-based quantitative PCR (qPCR). The kit is designed for automated high-throughput workflows, compatible with instruments such as KingFisher™ Flex.

Main Components

| No. | Component Name | Volume | Composition |
| --- | --- | --- | --- |
| 1 | Lysis Buffer | 26.5 mL × 1 bottle | Guanidine salt solution (<0.001%) |
| 2 | Binding Beads | 1.5 mL × 1 tube | Magnetic silica beads suspended in solution |
| 3 | Wash Buffer 1 | 35 mL × 1 bottle | Contains ethanol (40–70%) |
| 4 | Wash Buffer 2 | 15 mL × 1 bottle | Contains ethanol (<4%) |
| 5 | Elution Buffer | 1.5 mL × 1 tube | Low-salt buffer (<2.5%) |

Storage Conditions & Shelf Life: Store at 15°C-30°C, valid for 12 months.
Do not use after expiration date indicated on the label.

Applicable Instruments

For use with KingFisher™ Flex NAs extractors manufactured by Life Technologies (Thermo Fisher Scientific).
 Recommended model: KingFisher™ Flex (Shanghai Medical Device Registration No.: 20193073269, Registration Certificate No.: 2021012339)

Sample Requirements

Applicable sample types: throat swab, blood, serum, plasma, etc.
Samples should be collected, transported, and stored according to standard procedures.

Verification Method

Required Equipment & Materials

| Device Name | Quantity | Manufacturer | Product Code |
| --- | --- | --- | --- |
| KingFisher™ Flex | 1 | Thermo Fisher Scientific | A5040630 / C5400630 |
| KingFisher™ 96 Deep-Well Plate | ≥3 | Thermo Fisher Scientific | 95040450 |
| Magnetic Tip Comb | ≥3 | Thermo Fisher Scientific | 97002534 |
| Elution Plate (96-well) | ≥1 | Thermo Fisher Scientific | 4306311 |

1.1 Reagent Preparation

Prepare KingFisher Flex instrument and accessories.

Label 96-deep-well plates as: Sample Plate, Wash Plate 1, Wash Plate 2.

Add 100% ethanol and nuclease-free water to new containers to prepare 80% ethanol.

1.2 Reagent Plate Layout Example (per 96-well plate):

| Plate Position | Reagent | Volume/well |
| --- | --- | --- |
| 1 | Binding Beads (6 μL) + Lysis Buffer (200 μL) + Sample | 265 μL |
| 2 | Wash Buffer 1 | 1000 μL |
| 3 | Wash Buffer 2 (80% EtOH) | 1000 μL |
| 4 | Elution Buffer | 50 μL |

1.3 Binding Bead Preparation

Before use, vortex the bead suspension for at least 15 seconds to ensure proper mixing. If viscous or aggregated, place at 37°C for 5 minutes and re-vortex until evenly dispersed.

Prepare 6 μL of beads and 265 μL of lysis buffer per sample.

Product Performance Indicators:

This kit is verified on KingFisher Flex; average Ct value deviation from reference values is within ±2.

Precautions

For in vitro diagnostic use only. Follow the instructions strictly.

Use clean, RNase-free materials.

Avoid cross-contamination during liquid handling.

Ensure ethanol concentration is accurate when preparing Wash Buffer 2.

Store eluates at -20°C to -80°C if not used immediately.

Do not reuse any consumables.

24. Supplementary Materials M4: Testing methods

Products Comparison Experimental Protocols

1. Experimental Methods and Objectives

In NAs extraction using MNPs, the lysis buffer and MNPs are the two most critical reagents, while the wash buffer and elution buffer serve as complementary reagents. This experiment evaluates and compares the performance of MNPs products and reagent systems from Thermo Fisher, Daan Gene, and the AP-Lab.

2. Equipment and Materials

Equipment:

- Automated platform dispensing module
- Fully automated NAs extraction instrument
- SLAN fully automated medical PCR analyzer

Reagents:

**(1) Positive controls**:

- SARS-CoV-2 RNA (O-site, 105 copies/mL, -80°C)
- Influenza virus A virus positive control (105 copies/mL, -80°C)
- Influenza virus B virus positive control (105 copies/mL, -80°C)

**(2) Amplification reagents**:

- TransScript Ⅱ Probe One-Step qRT-PCR SuperMix (AQ321-01) and ORF1ab gene primers and probes (-20°C storage)
- Daan Gene Influenza virus A NAs detection kit (PCR fluorescence probe method, DA0230)
- Daan Gene Influenza virus B NAs detection kit (PCR fluorescence probe method, DA0240)

**(3) NAs extraction reagents**:

- Wash buffer
- Elution buffer

**(4) Chemical reagents**:

- Guanidine isothiocyanate
- Ammonium sulfate
- PEG 2000
- Triton
- Isopropanol
- 0.5M EDTA (pH 8.0)
- 1M Tris (pH 8.0)
- Ultrapure water

**(5) Consumables**:

- 96-well plates
- Magnetic rod sleeves
- Centrifuge tubes
- 8-strip PCR tubes with caps

3. Experimental Procedures

**(1) Solution Preparation:**

Prepare solutions according to the composition table given by the AP-Lab, Thermo Fisher and Daan Gene.

**(2) MNPs**:

Provided by the MNPs preparation unit: single-carboxyl 600 nm and multi-carboxyl 1000 nm beads.

(3) NAs Extraction:

1. Prepare four 96-well plates and label them 1-4.
2. Add the following volumes to specific wells:

- Plate 1: 200 µL MNPs storage solution (MB) in the first two wells of column 1.
- Plate 2: 500 µL lysis buffer in the first two wells of column 1.
- Plate 3: 400 µL wash buffer in the first two wells of column 1.
- Plate 4: 80 µL elution buffer in the first two wells of column 1.

1. Add 300 µL of diluted viral sample (10³ copies/mL) to the lysis wells in Plate 2, then proceed with NAs extraction using the automated instrument.
2. Set the extraction program with the following parameters:

| Step | Well | Description | Wait Time (min:sec) | Rod Sleeve | Mixing Time (min:sec) | Magnetic Separation (s) | Solvent (µL) | Mixing Speed | Heating Temp (°C) |
| --- | --- | --- | --- | --- | --- | --- | --- | --- | --- |
| 1 | 4 | Magnetic separation | 0:00 | √ | 0:10 | 50 | 200 | Fast | Off |
| 2 | 1 | Lysis | 0:00 | √ | 5:00 | 60 | 800 | Fast | 96 |
| 3 | 2 | Washing | 0:00 | × | 0:15 | 0 | 400 | Fast | Off |
| 4 | 3 | Elution | 2:00 | √ | 5:00 | 60 | 80 | Fast | 86 |
| 5 | 4 | Recovery | 0:00 | √ | 0:15 | 0 | 200 | Fast | Off |

**(4) PCR Amplification**

Details of the amplification steps for SARS-CoV-2, Influenza virus A, and Influenza virus B are outlined, including reagent compositions and thermal cycling parameters.
